# Investigation of Intramolecular Through-Space Charge-Transfer States in Donor–Acceptor Charge-Transfer Systems

**DOI:** 10.1021/acs.jpclett.1c00265

**Published:** 2021-03-15

**Authors:** Shiv Kumar, Larissa Gomes Franca, Kleitos Stavrou, Ettore Crovini, David B. Cordes, Alexandra M. Z. Slawin, Andrew P. Monkman, Eli Zysman-Colman

**Affiliations:** † Organic Semiconductor Centre, EaStCHEM School of Chemistry, University of St. Andrews, St. Andrews, Fife, U.K. KY16 9ST; ‡ Department of Physics, Durham University, Durham, U.K. DH1 3LE

## Abstract

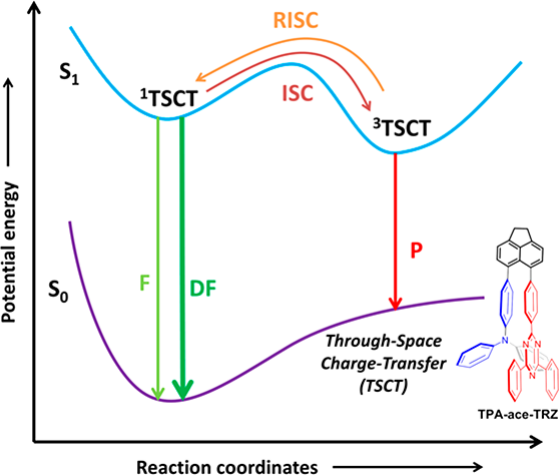

Commonly, thermally activated delayed fluorescence (TADF) emitters present a twisted donor–acceptor structure. Here, electronic communication is mediated through-bond via π-conjugation between donor and acceptor groups. A second class of TADF emitters are those where electronic communication between donor and acceptor moieties is mediated through-space. In these through-space charge-transfer (TSCT) architectures, the donor and acceptor groups are disposed in a pseudocofacial orientation and linked via a bridging group that is typically an arene (or heteroarene). In most of these systems, there is no direct evidence that the TSCT is the dominant contributor to the communication between the donor and acceptor. Herein we investigate the interplay between through-bond localized excited (LE) and charge-transfer (CT) states and the TSCT in a rationally designed emitter, **TPA-ace-TRZ**, and a family of model compounds. From our photophysical studies, TSCT TADF in **TPA-ace-TRZ** is unambiguously confirmed and supported by theoretical modeling.

The use of metal-free TADF emitters in organic light-emitting diodes (OLEDs) has attracted significant attention within the OLED community following the first reports from Goushi et al.^1^ and Uoyama et al.,^2^ which demonstrated the potential of E-type delayed emission exciplexes and donor–acceptor emitters to produce high-efficiency devices. The reason TADF emitters have become so attractive is due to their capacity to harvest up to 100% of triplet excitons.^3^ This is made possible by the small energy gaps (Δ*E*_ST_), generally less than 100 meV, among the lowest CT singlet, triplet, and local triplet excited states of these materials, enabling efficient upconversion of the electrically generated CT triplet states through a vibrationally coupled spin–orbit coupling mechanism where the local triplet excited state (T_1_) mediates the otherwise forbidden ^3^CT triplet to ^1^CT singlet transition via reverse intersystem crossing (RISC).^4,5^ TADF-OLEDs have achieved maximum external quantum efficiencies (EQE_max_) comparable with those of OLEDs based on phosphorescent emitters.^6^ The requisite small ^1^CT–^3^CT Δ*E*_ST_ is achieved within the emitter by spatially separating the HOMO and LUMO on donor (D) and acceptor (A) moieties, respectively, of D–A molecules, thereby minimizing the electron exchange integral of the frontier molecular orbitals.^7^ However, this strategy can lead to a compromise with the photoluminescence quantum yield (Φ_PL_) of the emitter due to a reduction of the oscillator strength (*f*) for this transition, which corresponds to a slower radiative decay rate. Therefore, a balance in modulating Δ*E*_ST_, *f*, and slow nonradiative decay is required to produce highly efficient TADF emitters^8^ and OLEDs.

While a typical intramolecular D–A TADF emitter is monomolecular, with its emissive singlet excited state having CT character, CT excited states can also be realized in intermolecular bimolecular exciplex systems, which likewise show TADF.^9,10^ A wide variety of intermolecular exciplex TADF emitters have been developed with appropriate combination of D and A molecules;^11−13^ however, several technical issues remain in converting exciplex emitters to high-efficiency OLEDs. These include low Φ_PL_ and very broad emission envelopes due to the inhomogeneity of the donor–acceptor distances in the exciplex.^14^

Designing TADF emitters based on TSCT is an alternative approach to overcoming many of the issues associated with exciplex-OLEDs.^15^ Compared to a traditional twisted intramolecular charge-transfer TADF emitter design, where D and A moieties are directly attached to each other and the CT transition takes place through-bond, in a TSCT-TADF emitter the D and A moieties are separated by a electronically benign spacer but remain in close proximity to each other such that electronic communication and electron transfer are mediated through-space in an analogous manner to exciplexes.^16^ This approach not only results in emitters with small Δ*E*_ST_ values but also overcomes the issues of inhomogeneous D–A distances in the exciplex emitters in thin films.

Herein we report a study of TSCT and model compounds using acenaphthene as a scaffold and triphenylamine (TPA) as the donor. TSCT TADF in **TPA-ace-TRZ** is unambiguously confirmed by comparison to the family of materials investigated. The structural, electrochemical, and photophysical properties of **TPA-ace-TRZ** were investigated and compared with those of model compounds **TPA-ace-CN**, **TPA-ace**, **TPA-ace-Br**, and **2TPA-ace**. From our photophysical studies, we observe changing local and CT emission, delayed fluorescence, and phosphorescence emission in these molecules, even at room temperature. With a focus on CT excited states, a mechanistic approach has been attempted to establish the structure–property relationship in these molecules.

The syntheses of **TPA-ace**, **2TPA-ace**, **TPA-ace-Br**, **TPA-ace-CN**, and **TPA-ace-TRZ** are outlined in Scheme 1. Each of the target materials has been characterized by a melting-point determination, ^1^H and ^13^C NMR spectroscopy, and mass spectrometry. Elemental analysis and HPLC analysis were used to evidence the purity of the emitters (Supporting Information).

**Scheme 1:**
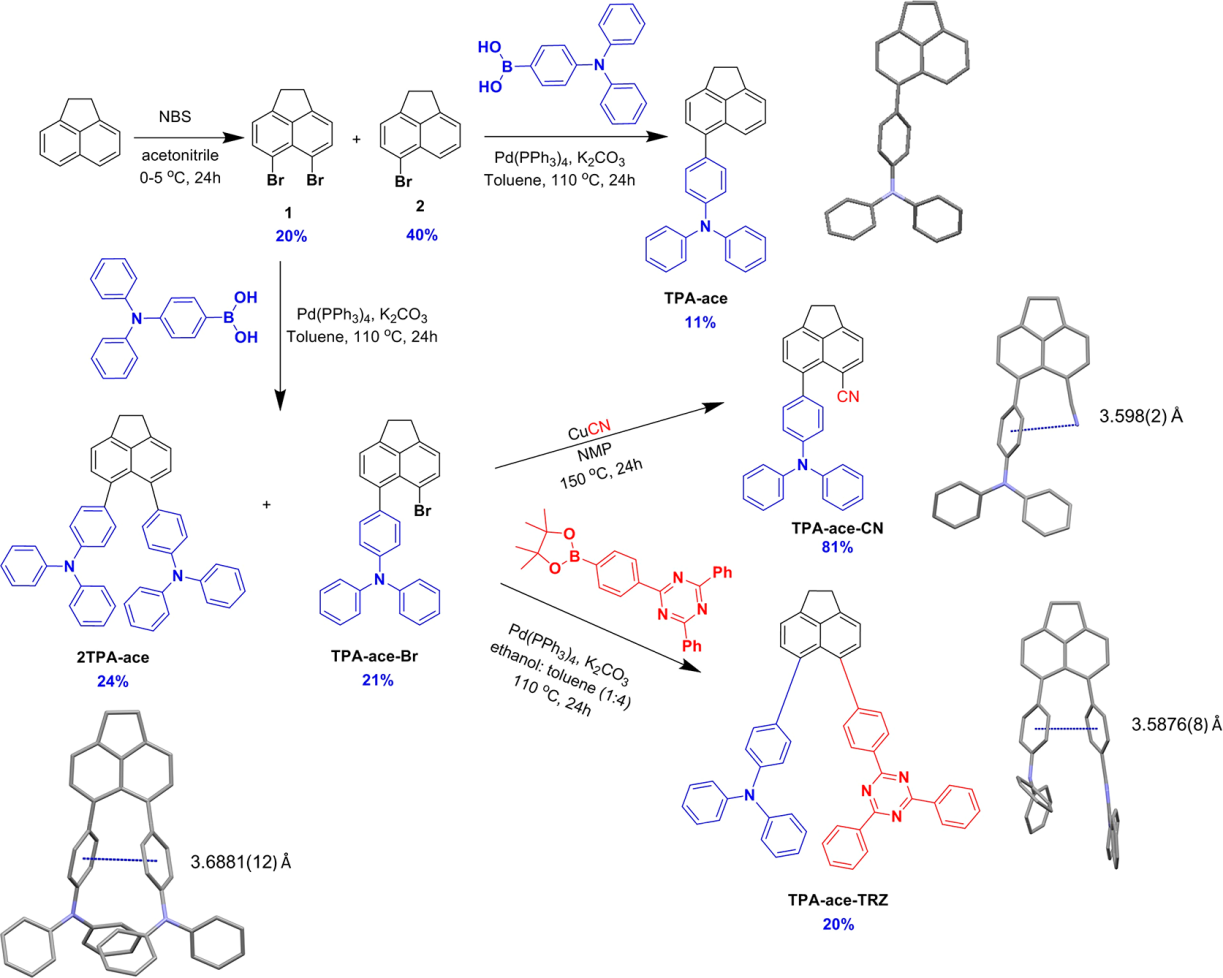
Synthesis Scheme for **TPA-ace**, **2TPA-ace**, **TPA-ace-Br**, **TPA-ace-CN**, and **TPA-ace-TRZ**, Including X-ray Structure Diagrams Showing Intramolecular Interactions The solvent molecule and hydrogen atom are omitted for clarity.

Single crystals of **TPA-ace**, **TPA-ace-CN**, **2TPA-ace**, and **TPA-ace-TRZ** were grown by the vapor diffusion of *n*-hexane into a saturated dichloromethane solution of the compound. The molecular structures were determined by single-crystal X-ray diffraction analysis and are shown in Scheme 1. Despite the design of enforced proximity between substituents of the acenaphthene, the flexibility of the ring system allowed sufficient splay to develop between substituents such that surprisingly few intramolecular interactions are observed. In **TPA-ace-CN**, there is the potential for a weak interaction between the cyano group and the bridging phenyl of the TPA [N···centroid distance of 3.598(2) Å], while in both **TPA-ace-TRZ** and **2TPA-ace** intramolecular π···π interactions occur between the proximal phenyl rings only of the substituents [centroid···centroid distances of 3.5876(8) and 3.6881(12) Å, respectively]. Three different patterns of intermolecular interactions are seen in the structures. In **TPA-ace**, head-to-head dimers are formed via pairs of weak CH···π interactions between two methylene hydrogens and the naphthalene (H···centroid distances of 2.87 and 2.94 Å). In both **2TPA-ace** and **TPA-ace-CN**, three-dimensional networks are formed from different combinations of weak interactions. In **2TPA-ace**, the network arises from four sets of CH···π interactions involving TPA-phenyl hydrogens and either naphthalene or TPA-phenyl π-systems (H···centroid distances of 2.69 to 2.90 Å). In **TPA-ace-CN**, the network is formed from two different CH···π interactions between phenyl hydrogens and the terminal phenyls of the TPA (H···centroid distances of 2.91 and 2.99 Å) as well as CH···N hydrogen bonds between one methylene hydrogen and the cyano nitrogen [H···N 2.54 Å, C···N 3.443(3) Å]. In **TPA-ace-TRZ**, there are three pairs of interactions: π···π interactions between the triazine and one of the TRZ-phenyl groups of an adjacent molecule [centroid···centroid distance of 3.361(8) Å] and two CH···π interactions involving both methylene and naphthalene hydrogens and either naphthalene or TRZ-phenyl π-systems (H···centroid distances of 2.83 and 2.91 Å). The combination of these leads to two-dimensional sheets in the *ac*-plane.

The ground-state geometries of each of the acenaphthene compounds were optimized in the gas phase at the PBE0/6-31G(d,p) level starting from the geometry obtained from the single-crystal X-ray diffraction analysis; that of **TPA-ace-Br** was optimized starting from an initial geometry drawn in *GaussView*. Time-dependent DFT calculations were performed within the Tamm–Dancoff approximation (TDA)^17^ using the ground-state optimized geometries. The energies and electron density distributions of the highest occupied and lowest unoccupied molecular orbitals (HOMO/LUMO) and the energies of the S_1_ and T_1_ states are shown in Figure 1. In **TPA-ace-TRZ**, **TPA-ace-CN**, and **TPA-ace-Br**, the HOMO is localized on the TPA moiety while the HOMO is delocalized over both the TPA and acenaphthene bridge in **TPA-ace** and **2TPA-ace**. In **TPA-ace-TRZ**, the LUMO is localized mainly on the TRZ unit while in **TPA-ace-CN** and **TPA-ace-Br** the LUMO is localized on the CN- or Br-substituted acenaphthene moiety. In the case of **TPA-ace** and **2TPA-ace**, the LUMO is delocalized across both the phenyl ring of the TPA and the acenaphthene. A large electron density overlap exists in **TPA-ace** and **2TPA-ace**, which is reflected in their large calculated Δ*E*_ST_ values; the Δ*E*_ST_ for **TPA-ace-Br** is also large due to a change in the nature of the T_1_ state, which is mainly described by a HOMO – 1 to LUMO transition that is of a localized excited (LE) character on the acenaphthene. Much smaller Δ*E*_ST_ values were observed for **TPA-ace-TRZ** and **TPA-ace-CN**, which is reflective of the significantly greater spatial separation of the electron density distributions of the HOMO and LUMO. Importantly, for **TPA-ace-TRZ** there is no predicted through-bond communication, and instead we hypothesize that there is a through-space electronic communication directly between the TPA and TRZ moieties. The predicted energy of the HOMO level for each of the emitters is around −5.00 eV, a reflection of a HOMO localized in all compounds on the TPA moiety, coupled with poor conjugation to the acenaphthene bridge, except for **TPA-ace-TRZ** (−4.87 eV) where there is significant stabilization. On the other hand, the energy of the LUMO level varies significantly depending upon the nature of the electron acceptor. There is a cluster of compounds, **TPA-ace**, **2TPA-ace**, and **TPA-ace-Br**, with associated LUMO values of between −0.85 and −0.99 eV, and then there are the two emitters, **TPA-ace-CN** and **TPA-ace-TRZ**, containing strong electron-withdrawing acceptors, that have LUMOs that are significantly stabilized at −1.56 and −1.68 eV, respectively. This same analysis is true for the S_1_ energy level as well where **TPA-ace**, **2TPA-ace**, and **TPA-ace-Br** possess high S_1_ energies ranging from 3.41 to 3.65 eV while **TPA-ace-CN** and **TPA-ace-TRZ** have significantly lower S_1_ energies of 2.87 and 2.51 eV, respectively. The triplet energies cluster over a narrow range between 2.65 and 2.82 eV while there is greater divergence in the singlet-state energies. This thus leads to two groups among these compounds where **TPA-ace**, **2TPA-ace**, and **TPA-ace-Br** have large Δ*E*_ST_ values and **TPA-ace-TRZ** and **TPA-ace-CN** possess significantly smaller Δ*E*_ST_ values.

**Figure 1:**
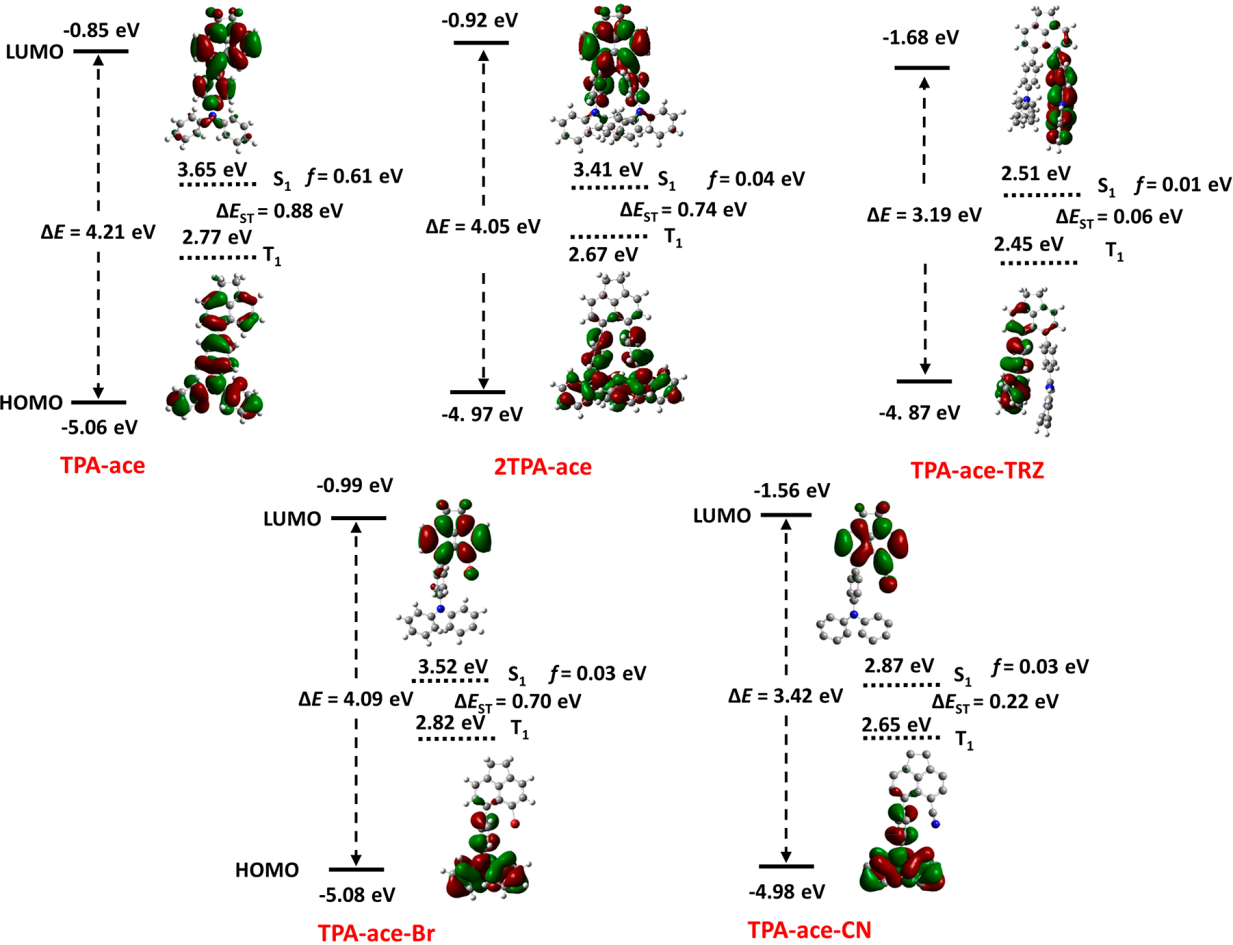
DFT-calculated ground-state (PBE0/6-31g(d,p)) and TDA-calculated excited-state energies, oscillator strengths, and electron density distributions (ISO value = 0.02) of the frontier molecular orbitals of the acenaphthene emitter derivatives.

The electrochemical properties of these compounds were investigated by cyclic voltammetry (CV) and differential pulse voltammetry (DPV). Anodic scans reveal a reversible oxidation that is centered on the TPA (Figure S35), whereas the reduction waves were irreversible and inconclusive for all and were therefore omitted. The oxidation potential of TPA has been previously estimated at 0.87 V vs SCE.^18^ After grafting the acenaphthene unit, the oxidation potentials obtained from the DPVs were found to be much lower than that of TPA and were calculated at 0.26, 0.37, 0.38, 0.44, and 0.47 V for **TPA-ace**, **2TPA-ace**, **TPA-ace-TRZ**, **TPA-ace-Br**, and **TPA-ace-CN**, respectively, which correspond to HOMO levels of −5.06, −5.17, −5.18, −5.24, and −5.27 eV, respectively. The addition of electron-withdrawing groups to the acenaphthene bridge stabilizes the oxidation potentials and reflects a certain degree of electronic coupling between the TPA and the acenaphthene bridge. The energies of the HOMO level determined from DPV are broadly in agreement with that predicted by DFT calculations.

Steady-state photophysical analysis was initially performed in dilute solution. Figure 2a shows the normalized optical absorption and emission spectra of **TPA-ace** in three different solvents. The **TPA-ace** absorption spectrum presents two peaks: a band centered at around 310 nm related to the acenaphthene scaffold^16,19^ and a second peak, at lower energy, associated with the π → π* transition of the delocalized **TPA-ace** system (Figure S36). With increasing solvent polarity, a minor red shift appears in the latter transition, whereas photoluminescence (PL) spectra exhibit a weak bathochromic shift and a change from a structured emission band to a Gaussian-shaped emission band. The small red shift could indicate that the bridge (**ace**) is not electronically decoupled and forms a weak through-bond charge-transfer (TBCT) state with the donor unit (**TPA)**. This we propose as a highly mixed ^1^LE/^1^CT state with low CT character because there is conjugation between D and A since they are not orthogonally disposed, as found by DFT. In Figure 2b, we see that the blue-edge absorption feature in **TPA-ace-Br** is blue-shifted compared to **TPA-ace**, potentially indicating weaker conjugation between the **TPA** and **ace-Br** units. The PL spectrum of **TPA-ace-Br** yields dual emission in higher-polarity DCM. The high-energy band red shifts slightly with increasing solvent polarity, a behavior that matches very well with the mixed ^1^LE/^1^CT state observed in **TPA-ace**. The second, more intense, emission band at around 540 nm observed in DCM is assigned to a state with stronger CT character (stabilized by the high-polarity solvent), in line with the stronger acceptor strength of the **ace-Br** unit. With the addition of a second **TPA** donor unit, **2TPA-ace**, changing from a donor–bridge (D–B) to a donor–bridge–donor (D–B–D) structure, the absorption and emission spectra remain similar to those of **TPA-ace** (Figure 2c). This similarity of the optical behavior is expected because the new **TPA** unit does not increase the strength of the donor, and the bridge effectively acts as a very weak acceptor unit and hence a similar ICT state forms. Careful inspection of the spectra, however, shows that the emission onset is red shifted by 822 cm^–1^ (in methylcyclohexane, MCH) and the band shape loses structure compared to the structured **TPA-ace** spectra. This we identify as the effect of an interaction between the two TPA units (i.e., a weak intramolecular dimer state), as supported by the X-ray structure. Concentration-dependent measurements (Figure S37) show that this is a purely monomolecular property, fully supporting the assumption that this is a cofacial intramolecular interaction between the two weakly overlapping TPA units.

**Figure 2:**
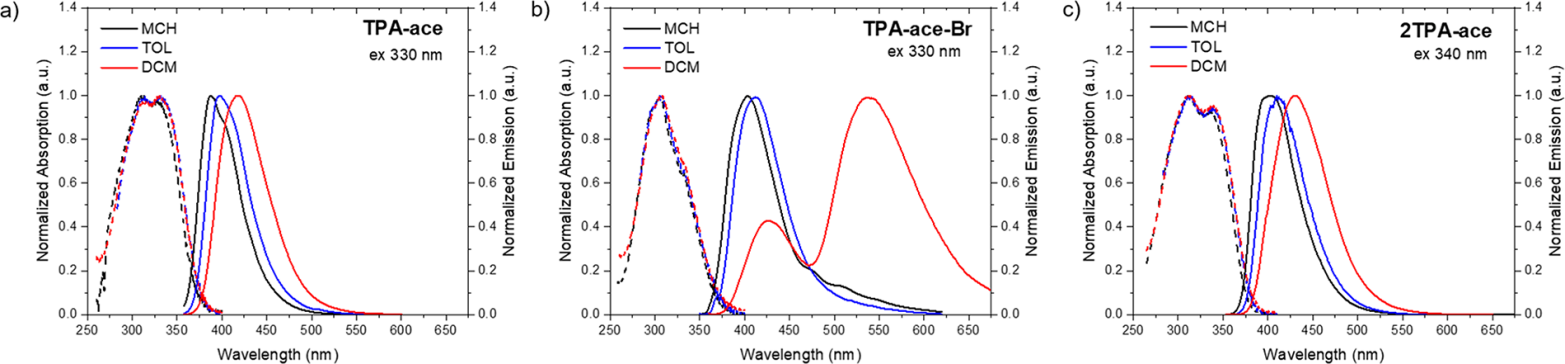
Normalized UV–vis absorption (dashed lines) and PL spectra (solid lines) of (a) **TPA-ace**, (b) **TPA-ace-Br**, and (c) **2TPA-ace** molecules in different solvents at a concentration of 20 μM (λ_exc_ = 330 nm for **TPA-ace** and **TPA-ace-Br**; λ_exc_ = 340 nm for **2TPA-ace**).

As with **TPA-ace-Br**, the absorption of **TPA-ace-CN** (Figure 3a) also has a weak lowest-energy transition, ascribed to the delocalized **TPA-ace** unit, that is much less intense than that observed in **TPA-ace** and **2TPA-ace**. The **TPA-ace-TRZ** absorption spectra (Figure 3b) reflect the introduction of the **TRZ** group with the appearance of a strong **TRZ** absorption band at 270 nm^20^ and, as with the other members of the series, a lower-energy band at 340 nm from the **TPA-ace** system. Furthermore, unlike the other compounds in this study, we observe a low-intensity tail/band stretching from 390 to 440 nm, characteristic of a direct CT transition^21^ and strongly indicative of a ground state through-space interaction between the **TRZ** and **TPA** units^16^ and fully in line with the large calculated permanent dipole moment compared to the other four materials. With the introduction of stronger acceptor groups, cyano and **TRZ**, a broader emission band is observed, even in nonpolar MCH (Figure 3a), and a larger red shift was obtained with increasing solvent polarity, which indicates that the presence of the electron-accepting **ace-CN** unit contributes to the formation of a stronger CT character excited state. DFT calculations reveal that this is indicative of a through-bond CT state between a stronger D–A pair. The emission spectra of **TPA-ace-TRZ** across the range of solvents show a red-shifted maximum and much stronger positive solvatochromism, even in MCH, which indicates that the **TRZ** unit has a much stronger acceptor character than the bridge (manifesto). This different behavior strongly suggests that this is a TSCT state between the **TRZ**···**TPA** units that has ground-state electronic coupling.

**Figure 3:**
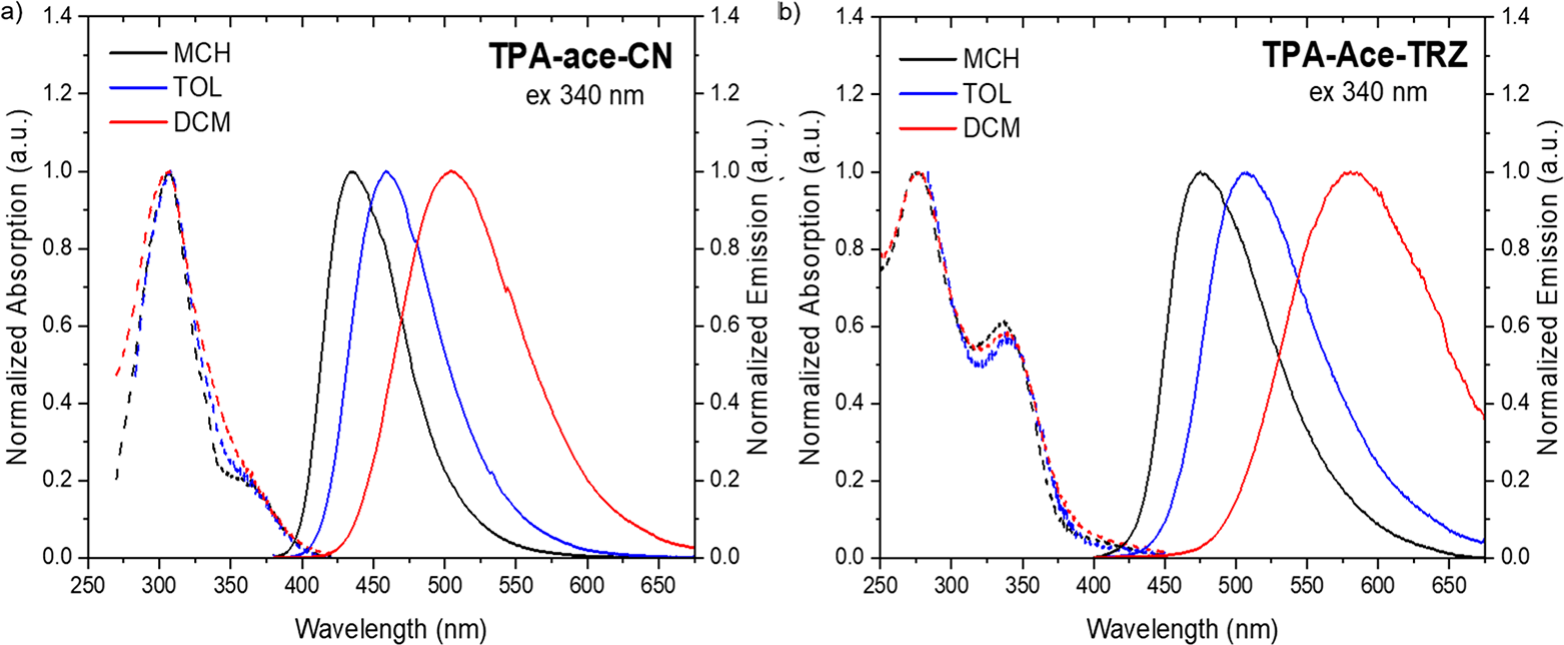
Normalized UV–vis absorption (dashed lines) and PL spectra (solid lines) of (a) **TPA-ace-CN** and (b) **TPA-ace-TRZ** molecules in different solvents (λ_exc_ = 340 nm) at a concentration of 20 μM.

Turning to time-resolved PL spectra, we investigated the dynamics of the excited states in this family of CT materials dissolved in toluene. Figure 4a shows the time-resolved normalized emission spectra of **TPA-ace** in toluene at room temperature. In the first few nanoseconds, an emission band centered at 425 nm related to a short-lived LE state of the **TPA** donor unit mixed with a small amount of CT character is observed. This local emission decays rapidly with a broader band at around 500 nm growing in, indicating a weak, prompt ICT state (Figure S38). This CT state is transient and rapidly decays within 30 ns. This behavior is characteristic of a TICT state^22^ between the **TPA** and **ace** units, where the phenyl ring inking the **TPA** and **ace** units rotates from 48° to nearly orthogonal, fully breaking the conjugation of the two to stabilize the CT state for a short time. Analogous to the steady-state PL measurement, **2TPA-ace** shows a time-resolved spectrum very similar to that of **TPA-ace** (Figure 4c), with a minor enhancement of the CT contribution potentially due to the through-space interaction of the two D units. Figure 4d shows the decay curve of these molecules in degassed toluene (λ_exc_ = 355 nm). In the nanosecond regime, **TPA-ace** and **2TPA-ace** present fast emission decay from the mixed LE/CT state contribution, with τ_PL_ = 1.57 and 2.34 ns, respectively, showing the predominant LE character of this state. For **TPA-ace-Br**, at early times, two emission bands are observed simultaneously: an LE/CT emission at 425 nm (observed only in the first time window) analogous to that observed for **TPA-ace** and **2TPA-ace** (Figure S38) as well as a prompt low-energy CT emission at 525 nm, with τ_PL_ = 9.4 ns (Table 1), but again it is a transient state. This CT band is much more intense than the transient species in **TPA-ace** and **2TPA-ace** and has a longer lifetime. This we interpret as **ace-Br** being a sufficiently strong acceptor to permit the formation of a stable and more red-shifted through-bond CT state; the steric hindrance of the Br atom introduces a large torsion angle of 81° between **TPA** and **ace-Br**, corroborated by our DFT calculations. However, because we observed this state at about the same energy in toluene and DCM for all of these materials, we surmise that it must have weak CT character (i.e., high LE character), so again we assign this band to a mixed LE/CT state, which is red-shifted via the additional charge decoupling coming through the increased orthogonality between effective D (**TPA**) and A (**ace-Br**) groups. After an interval without emission (below the noise floor of the iCCD), at times after 50 μs, a delayed fluorescence (DF) emission having the same onset energy as the prompt CT band can be observed. In addition, there is a second weaker band centered at around 590 nm that we ascribe to phosphorescence. Because the energy gap between the triplet and singlet states (Δ*E*_ST_) is large, ca. 260 meV, as described later, any RISC would be very weak and slow. This is why we observe dual emission in the form of TADF and room-temperature phosphorescence (RTP) in this case. We note that this dual emission behavior is in part observable because of the heavy atom effect of the Br, which increases the ISC, yielding a large triplet population, which in turn allows weak RISC to be observed. Again, through enhanced spin–orbit coupling, the phosphorescence radiative decay increases such that we can simultaneously observe RTP from the large triplet population.

**Figure 4:**
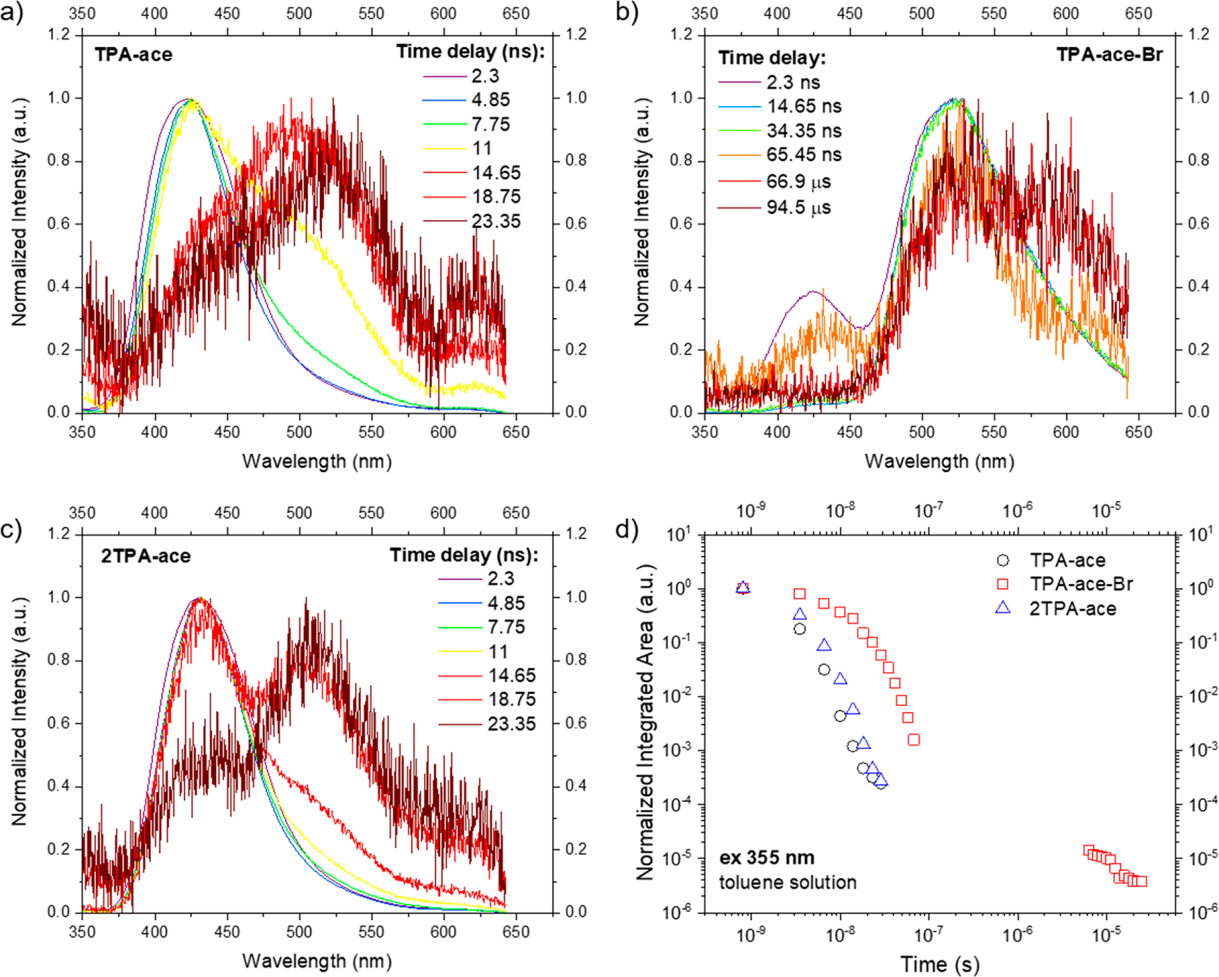
Time-resolved normalized PL spectra of (a) **TPA-ace**, (b) **TPA-ace-Br**, and (c) **2TPA-ace** in toluene solution at a concentration of 20 μM. (d) Time-resolved PL decay curves in the entire region of analysis. λ_exc_ = 355 nm. (b) The spectrum recorded for **TPA-ace-Br** at 66 ns is very weak, and the feature between 380 and 460 nm is the dark signal from the iCCD, not LE emission from **TPA-ace-Br**.)

**Table 1:** Photophysical Properties of the Molecules in Toluene Solution

		^1^CT			
emitter	*E*_g_/eV^a^	/nm	/eV	Φ_PL_^d^	τ_PL_/ns^e^
**TPA-ace**	3.29	495^b^	2.50	0.67	1.57
**TPA-ace-Br**	3.33	522^c^	2.37	0.02	9.49
**2TPA-ace**	3.26	513^b^	2.41	0.51	2.34
**TPA-ace-CN**	3.09	470^c^	2.63	0.67	5.96
**TPA-ace-TRZ**	3.22	518^c^	2.39	0.17	9.6 (72.6%) and 51.0 (27.4%)

a Optical band gap estimated from the absorption spectra onset of the first main (exciton) absorption band.

b Values obtained from the peak of the time-resolved PL spectra at a 11 ns delay, after subtracting the pure LE spectra (Figure S37).

c Values obtained from the peak of the time-resolved spectra at a 14 ns delay, after stabilization of the CT state (Figure S39).

d Photoluminescence quantum yield in degassed solution at room temperature. (Standard: quinine sulfate in 0.1 M H_2_SO_4_, Φ_PL_ = 0.54.)

e Lifetimes associated with the monoexponential or biexponential decay fitting (Figure S38).

Turning to the time-resolved PL spectra of **TPA-ace-CN**, there is only one emission band over the observed time window originating from the prompt CT state (Figures 5a and S36b). Despite this CT state having a higher energy than the transient CT state seen in **TPA-ace**, the introduction of the CN unit resulted in a stronger, longer-lived CT state with fast electron transfer so that we observe no LE emission within our time resolution. One possible reason for this could be that the CT state in **TPA-ace-CN** has a greater spatial charge separation resulting in a smaller Coulomb attraction energy and so a larger total amount of CT energy than the transient species in **TPA-ace**.^14^

**Figure 5:**
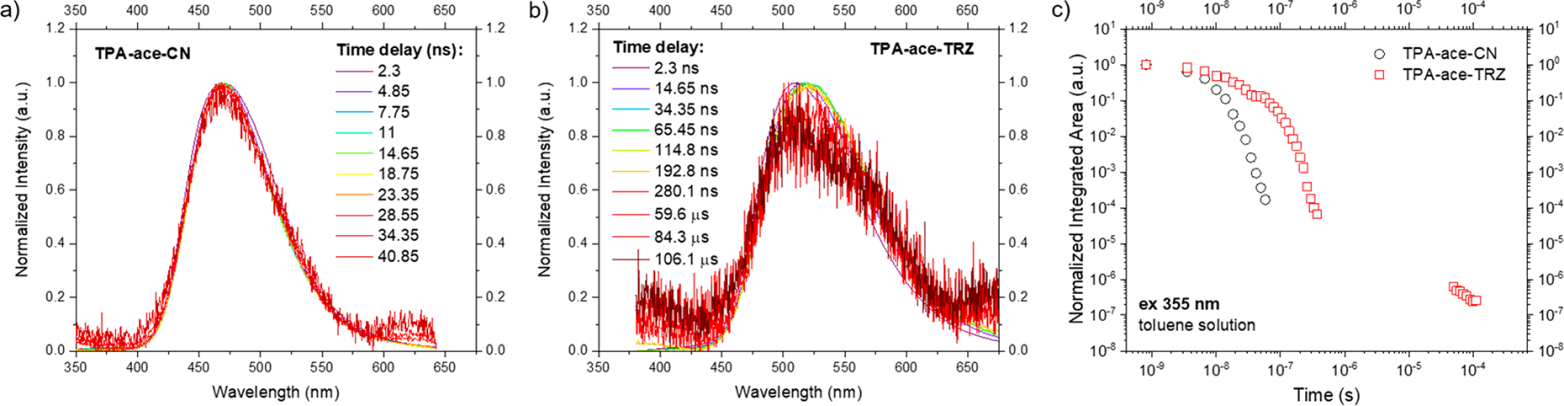
Time-resolved normalized emission spectra of (a) **TPA-ace-CN** and (b) **TPA-ace-TRZ** in toluene solution at a concentration of 20 μM. (c) Time-resolved PL decay curves in the entire region of analysis. λ_exc_ = 355 nm.

Figure 5b shows the time-resolved PL spectra of **TPA-ace-TRZ**. Initially, an emission band at around 500 nm is observed, associated with the same transient CT state as observed in **TPA-ace**. As the time delay increases, a small red shift is seen with an isoemissive point at 515 nm. This change in the CT states occurs over 35 ns. From the emission decay curve, we observe a clear biexponential decay with τ_PL_ = 9.6 and 51 ns (Figure 5c), indicating emission from two different CT states. We interpret this as the initial through-bond CT state (with moderate D–A dihedral angles of 48° between **TPA** and **ace** and 57° between **ace** and **TRZ**) decaying rapidly to leave a more stable TSCT state, as indicated by the observation of an isoemissive point at ca. 505 nm in the time-resolved spectra. Measurable delayed emission is seen over 100 μs, having the same emission spectrum as the prompt decay ascribed to DF. Because this emission is measured in dilute toluene solution, it is several orders of magnitude too fast and highly unlikely to be triplet-triplet annihilation (TTA). If TTA were efficient, then we would have expected to see it in all materials, which we clearly do not. Thus, we are convinced that the data provides compelling evidence that this is TADF coming from the TSCT observed only in **TPA-ace-TRZ**. Furthermore, the weak DF from very low RISC is attributed to the large Δ*E*_ST_ (*vide infra*). The decay curve of **TPA-ace-CN** (Figure 5c) is monoexponential, with a τ_PL_ of 6 ns, assigned to the single prompt CT emission.

Table 1 summarizes the photophysical properties of the five molecules in toluene solution. Optical band gaps remained almost constant regardless of structure, at around 3.3 eV, except for **TPA-ace-CN**, which presented a smaller gap of 3.09 eV, revealing the expected impact of the presence of the stronger electron-accepting cyano group in line with calculations. Despite this strong acceptor character, **TPA-ace-CN** showed weaker positive solvatochromism when compared to **TPA-ace-TRZ** due to a larger charge-separation distance and hence a smaller induced dipole moment. **TPA-ace-Br** and **TPA-ace-TRZ** presented smaller Φ_PL_ values, supporting the increases in ISC and the triplet population in these materials.

To further understand the properties of this family of materials, we also investigated the three members with the CT-character excited states in high-polarity solutions of degassed DCM. In the case of **TPA-ace-Br**, we observe that the spectral shift going from toluene to DCM for the low-energy emission band is only some 20 nm, far smaller than for the other materials. This clearly calls into question whether this low-energy state has CT character. From the phosphorescence data given below, the Δ*E*_ST_ in DCM should close substantially. Figure S41 shows the oxygen-dependent steady-state emission from **TPA-ace-CN**, **TPA-ace-Br**, and **TPA-ace-TRZ** in DCM. Both **TPA-ace-CN** and **TPA-ace-Br** show very strong emission quenching in oxygen, whereas **TPA-ace-TRZ** shows a much smaller quenching, consistent with singlet quenching by oxygen in the latter compound. Figure S42 shows a comparison of the emission spectra decay kinetics in DCM and toluene. In all cases, we observe only a prompt decay in DCM (except for a very weak hint of delayed emission in **TPA-ace-Br**, Figure S42), with lifetimes very similar to those found in toluene for **TPA-ace-Br** and **TPA-ace-TRZ** (the latter having two components, the longest with a lifetime of 40–50 ns, Figure S40), whereas the lifetime of **TPA-ace-CN** increases nearly 3-fold (Figure S43). The large oxygen quenching observed in **TPA-ace-CN** and **TPA-ace-Br** is not due to the quenching of triplet states that would give rise to DF but may simply reflect efficient singlet-state quenching in solution for excited states with very long lifetimes.

The solid-state photophysical properties of the emitters were analyzed in a ZEONEX matrix, a low-polarity neutral polymer host. Films were fabricated by drop-casting method at a concentration of 1 wt % of emitter to host. As observed in the solution measurements, **TPA-ace** had a short-lifetime ^1^LE/^1^CT emission at earlier times, which decays rapidly to leave a residual broad ^1^CT band centered at around 480 nm, which itself decays within 20 ns (Figure 6a). Despite **2TPA-ace** presenting results similar to those of **TPA-ace**, the time-resolved spectra (Figure 6c) showed a narrowed line width mixed LE/CT emission, which again decays rapidly to leave a ^1^CT band having higher intensity that is red-shifted by ca. 15 nm compared to that of **TPA-ace**. This corroborates our view that the interaction between the two **TPA** units enhances the probability of emission from the CT state. The time-resolved PL spectra of **TPA-ace-CN** (Figure 6b) shows an initial ^1^CT emission that red shifts with increasing time delay, indicating the apparent relaxation of ^1^CT emission due to a distribution of different molecular conformations with different decay times.^21,23^ This is typical behavior for a TADF molecule where the CT state D–A dihedral angle takes a range of values. Phosphorescence spectra (black lines) were recorded at a long time delay (80 ms), at 80 K, where the onsets of the spectra were used to obtain the ^3^LE energy of the molecules (Table 2). In all cases, large singlet–triplet energy gaps were found. The PL kinetic decay curves (Figure 6d) show that these molecules present a rapid decay, giving only prompt emission at room temperature, and no long-lived DF was found, analogous to the behavior in solution.

**Figure 6:**
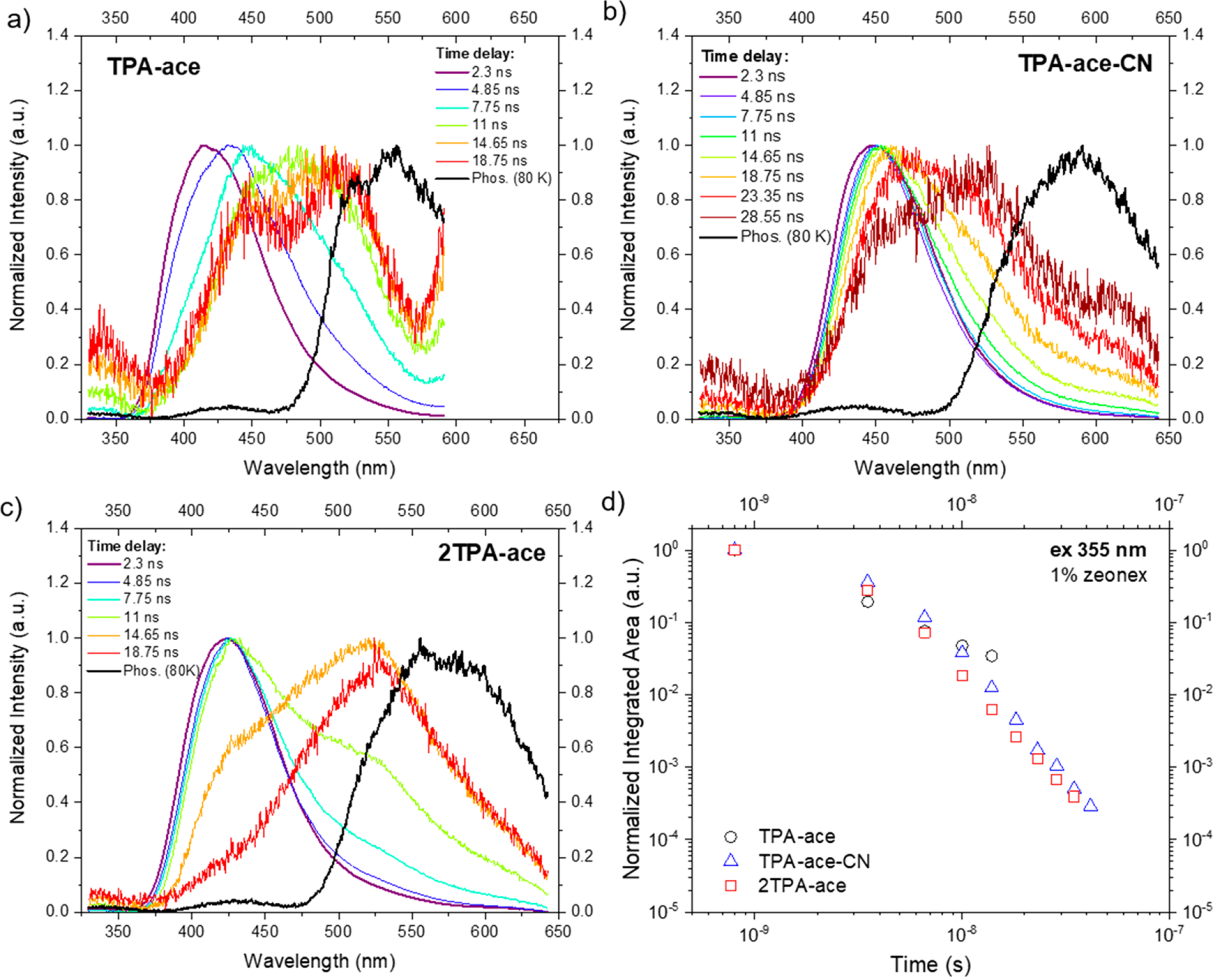
Time-resolved normalized emission spectra of (a) **TPA-ace**, (b) **TPA-ace-CN**, and (c) **2TPA-ace** in 1 wt % ZEONEX films. λ_exc_ = 355 nm. (d) Time-resolved PL kinetic decay curves in the entire region of analysis. The curves were obtained with 355 nm excitation. Note that the **TPA-ace** and **TPA-ace-CN** spectra after 10 and 20 ns, respectively, are very weak, and clear oscillatory dark noise from the iCCD can be seen. This is not intrinsic structure of the CT emission bands.

**Table 2:** Photophysical Properties of the Molecules in 1 wt % ZEONEX Films

emitter	^1^CT/eV^a^	^3^LE/eV^b^	Δ*E*_ST_/eV^c^	Φ_PL_^d^	τ_p_/ns^e^	^e^τ_d_/ms^e^
**TPA-ace**	2.99	2.53	0.46	0.37		
**TPA-ace-Br**	2.77	2.51	0.26		8.05	0.33 (90.6%)
						2.05 (9.4%)
**2TPA-ace**	2.95	2.55	0.40	0.36	1.93 (92.1%)	
					4.39 (7.9%)	
**TPA-ace-CN**	2.99	2.47	0.52	0.20	2.55 (97.0%)	
					7.50 (3.0%)	
**TPA-ace-TRZ**	2.94	2.46	0.48	0.12	6.9 (73%)	
					24.7 (27%)	

a Values estimated from the onset of time-resolved PL spectra after the stabilization of ^1^CT or deconvoluted in order to obtain only the ^1^CT onset (Figure S42).

b Values estimated from the onset of phosphorescence spectra. Spectra were collected with an 80 ms time delay and measured at 80 K.

c Δ*E*_ST_ = ^1^CT – ^3^LE.

d Φ_PL_ measured for spin-coated films with a concentration of 1 wt % in the ZEONEX matrix using an integrating sphere connected to a Fluorolog-3.

e Lifetimes estimated from monoexponential and biexponential decay fitting of the prompt and delayed regimes, respectively (Figure S46).

The time-resolved PL spectrum of **TPA-ace-Br** in the ZEONEX matrix is presented in Figure 7a, and its behavior was found to be very similar to that observed in solution. At early times, there is a very short-lived band at around 400 nm, and a highly red-shifted band at 500 nm emits until 65 ns. In the first time window, where clear dual emission is observed, the low-energy band has a pronounced blue-edge component and is structured. This suggests that it has mixed LE/CT character and a pure CT state, which we observe within our time resolution even at 80 K. The (relaxed) CT band possesses the same onset energy in ZEONEX as in toluene and is also very similar to that in DCM, indicating that it has a very small transition dipole moment, which aligns with the calculated TDM of 1.63 D. Moreover, after 100 μs, room-temperature phosphorescence grows in, contrary to solution measurement where mixed DF and phosphorescence emissions are seen at these times. This would point to greater stabilization of the CT state in toluene through the solvatochromic stabilization effect. Figure 7b shows the time-resolved PL spectra of **TPA-ace-TRZ**. Initially, during the prompt fluorescence regime, a relaxation of the ^1^CT band is observed, which decays after a few hundred nanoseconds. The onset of the CT emission is blue-shifted by 20 meV in ZEONEX compared to in toluene solution (Figure S44); we again observe an isoemissive point at 505 nm (Figure S45e). Thus, the CT emission observed in ZEONEX is predominantly from the through-bond state, with the formation of the (lower-energy) TSCT state hindered by the host matrix. This would indicate that reorganization of the TPA and TRZ moieties of the **TPA-ace-TRZ** must occur to enable the TSCT state to be accessed, which is hindered in ZEONEX. At later times, a very weak emission appears with an energy onset similar to that of phosphorescence, observed at late delay times at 80 K, indicative of inefficient RTP and not DF.

**Figure 7:**
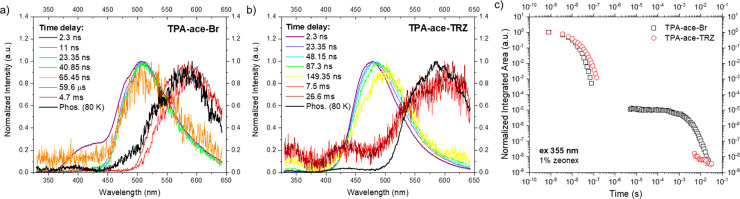
Time-resolved normalized emission spectra of (a) **TPA-ace-Br** and (b) **TPA-ace-TRZ** in 1 wt % ZEONEX films. (c) Time-resolved PL kinetic decay curves. λ_exc_ = 355 nm.

Different regimes were observed in the decay curves (Figure 7c), assigned to prompt ^1^CT emission and a delayed emission component. While the delayed emission component of **TPA-ace-TRZ** is observable only at very long times (tens of milliseconds) and has a very weak contribution ascribed to phosphorescence, **TPA-ace-Br** makes a strong RTP contribution, confirming that attaching a heavy atom such as Br to the molecule significantly enhances the intersystem crossing (ISC) in this system.

Thin-film photophysical data are summarized in Table 2. Here, the energies of the S_1_ and T_1_ states were determined using the onset of the time-resolved spectra at room temperature (S_1_) and low temperature (80 K) (T_1_). The ^3^LE values of all molecules are slightly different but within error are around 2.53 eV for **TPA-ace**, **TPA-ace-TPA**, and **TPA-ace-Br**, implying that the structural modifications have a minimal influence on the energy and location of the local triplet state. For **TPA-ace-CN** and **TPA-ace-TRZ**, the triplet energy is lower by some 5–10 meV. At 80 K, the phosphorescence is structured for **TPA-ace** and **2TPA-ace** and is assigned to the lowest-energy ^3^LE state of the **ace** unit;^24^ however, when acenaphthene is further substituted, the structure is lost. This identification is fully consistent with the large heavy-atom effects we observe in **TPA-ace-Br** enhancing this **ace** phosphorescence. The Δ*E*_ST_ values are higher than 0.4 eV in all cases except for **TPA-ace-Br**, where Δ*E*_ST_ is 0.26 eV; thus, it is not surprising that little or no DF is found in these compounds apart from **TPA-ace-Br**, where it is weak. The thin-film Φ_PL_ values, measured under a N_2_ atmosphere, are higher for **TPA-ace** and **TPA-ace-TPA** than for the other compounds under study, which have emission mainly coming from a mixed ^1^LE/^1^CT state. For the molecules with stronger CT character, **TPA-ace-CN** and **TPA-ace-TRZ**, the Φ_PL_ is significantly lower, indicating that the more stable CT states result in higher ^3^CT triplet state formation, which cannot be harvested by RISC. The transient PL decay curves were fitted by single or double exponentials; the very rapid decay of the emission of **TPA-ace** precluded the estimation of the prompt lifetime. **2TPA-ace** presented two different lifetimes: a faster one with a larger contribution of around 2 ns from the mixed LE/CT state and a slower, smaller contribution term from the CT state, with a lifetime of around 4 ns. The prompt emission observed in **TPA-ace-CN** also has two components ascribed to the mixed LE/CT state and the relaxed ^1^CT. **TPA-ace-TRZ** has two characteristic lifetimes corresponding to the fast-decaying through-bond CT state and longer-lived TSCT state. On the other hand, **TPA-ace-Br** exhibited a single CT state lifetime in the prompt regime, while in the delayed regime it had two exponential lifetimes on the order of milliseconds and assigned to RTP.

Time-resolved PL decays of the ZEONEX films were also measured at 80 K (Figure S47). For **TPA-ace**, **2TPA-ace**, and **TPA-ace-CN**, as at room temperature, the 490 nm mixed ^1^LE/^1^CT state decay is very rapid, leaving a transient CT state at 520 nm which itself has totally decayed within 100 ns. In particularly, **TPA-ace-CN** presented a weak, shorter-lifetime prompt CT state emission at 80 K, when compared with room-temperature measurements. This would indicate that a twisting motion is necessary, possible in the open ZEONEX polymer network, in order to stabilize the CT state in **TPA-ace-CN**, and as a result of steric hindrance caused by the CN group, this is thermally activated. Phosphorescence is observed in the millisecond time range. The CT emission from **TPA-ace-Br** at 80 K was found to be highly structured but at the same onset energy as at RT. This surprising observation suggests that this low-energy (weak) CT state is of mixed LE/CT character with a high LE contribution at 80 K.^25^ Finally, similar to the observed RT behavior, **TPA-ace-TRZ** showed initial CT emission, changing to a second low-energy CT state over tens of nanoseconds. An isoemissive point was observed, but the processes occur on a slower time scale, ca. 200 ns, indicative of a conformational reorganization of the TPA and TRZ moieties to stabilize the TSCT state, which is itself observed to live longer at low temperature. In all cases, well-resolved phosphorescence is observed at long times from which accurate ^3^LE energies are calculated.

To understand why we observe little or no TADF from the TSCT state in **TPA-ace-TRZ**, even in DCM where the Δ*E*_ST_ becomes rather small, we consider where the lowest-energy triplet state of the molecule resides. This is found to be the local triplet state of the **ace** bridge (thus a Br attached to the **ace** gives a heavy-atom enhancement to the **ace** phosphorescence), and in **TPA-ace-TRZ**, the **ace** unit is orthogonal and electronically decoupled from D and A, especially in the TSCT conformation. Hence, in this conformation ^1^CT and ^3^CT will be degenerate^26^ and triplet harvesting must occur through the vibronic-coupling spin–orbit coupling (SOC) mechanism^4,5^ as in D–A and exciplex^27^ TADF systems. However, in the case of **TPA-ace-TRZ**, the potentially mediating isoenergetic local triplet state is the **ace** bridge triplet, which is orthogonal to both D and A and thus cannot efficiently couple to the TSCT states. Therefore, even though **TPA-ace-TRZ** has a strong TSCT, it cannot produce TADF because of the lack of coupling to a mediating triplet state. From this we see that TSCT D–A pairs give rise to TADF through the vibronic coupling SOC mechanism, as do through-bond D–A systems and exciplex molecules.^28^

In summary, we report a **TPA-ace-TRZ** compound which we unambiguously demonstrate emits via a TSCT state. We compared the photophysical properties of this compound with several model systems: **TPA-ace**, **TPA-ace-Br**, **2TPA-ace**, and **TPA-ace-CN**. In all five compounds, there is an emissive mixed through-bond LE/CT state with differing levels of LE and CT mixing: **TPA-ace** shows the strongest degree of LE character while **TPA-ace-TRZ** exhibits the strongest CT character. Time-resolved measurements showed that the introduction of a second TPA donor onto the ace unit in **2TPA-ace** resulted in intramolecular dimer formation causing a relative enhancement of the overall ICT contribution, while the addition of the electron-accepting CN unit in **TPA-ace-CN** resulted in a pure-blue ICT emission. **TPA-ace-Br** also was shown to have a strong ICT state in the prompt region, the result of the nearly orthogonal conformation between the TPA and the **ace-Br** groups in the ground state. However, because of the relatively large Δ*E*_ST_ along with the existence of the heavy bromine atom, a dual TADF/RTP emission appeared weakly in solution, and with dominant RTP character in the solid state. The TSCT state, uniquely observed in **TPA-ace-TRZ**, exhibited a small delayed contribution in toluene solution, the result of a Δ*E*_ST_ being above 200 meV. Our study provides one of the few clear experimental demonstrations of the existence of a TSCT state, one that is corroborated by extensive DFT calculations. Our study also reveals the intimate interplay that the bridging ace group has on mediating both the through-bond ICT state and the TSCT state.

## Supporting Information Available

General experimental procedures, synthesis, melting point, NMR spectra, HRMS spectra, elemental analysis report, HPLC analysis report, crystallographic data (CCDC 2041945-2041948), molecular orbitals, and Cartesian coordinates of optimized ground states and additional photophysical data (PDF)

## Notes

The research data supporting this publication can be accessed at https://doi.org/10.17630/0ae146df-b0f9-4590-9e7e-c30191cc4461.
