# Supplementary material for: Investigation of Intramolecular Through-Space Charge-Transfer States in Donor–Acceptor Charge-Transfer Systems
Source: J Phys Chem Lett. 2021 Mar 15;12(11):2820–30. doi: 10.1021/acs.jpclett.1c00265 (PMC8041366; doi:10.1021/acs.jpclett.1c00265)
Supplement: Supplementary file 1 — jz1c00265_si_001.pdf [file jz1c00265_si_001.pdf]

Supporting Information

Investigation of Intramolecular Through-Space Charge-Transfer States in Donor-Acceptor Charge-Transfer Systems

Shiv Kumar,[a] Larissa Gomes Franca,[b] Kleitos Stavrou,[b] Ettore Crovini,[a] David B. Cordes,[a] Alexandra M. Z. Slawin,[a] Andrew P Monkman[b] and Eli Zysman-Colman*[a]*

[a] Organic Semiconductor Centre, EaStCHEM School of Chemistry, University of St Andrews, St Andrews, Fife, UK, KY16 9ST, Fax: +44-1334 463808; Tel: +44-1334 463826; E-mail: eli.zysman-colman@st-andrews.ac.uk; URL: http://www.zysman-colman.com

[b] Department of Physics, Durham University, Durham, UK, DH1 3LE; Tel: +44-191 33 43616; E-mail: a.p.monkman@durham.ac.uk

Table of Contents

Experimental section **S3**

General synthetic procedures: *S3*

Theoretical calculations: *S3*

Electrochemical characterization: *S4*

Sample preparation *S4*

Photophysical characterization *S4*

Synthesis **S5**

5-Bromoacenaphthene ***(Br-ace)*** *S5*

5,6-Dibromoacenaphthene ***(DBr-ace)*** *S5*

4-(1,2-dihydroacenaphthylen-5-yl)-N,N-diphenylaniline ***(TPA-ace)*** *S6*

4,4'-(1,2-dihydroacenaphthylene-5,6-diyl)bis(N,N-diphenylaniline) ***(2TPA-ace)*** *S7*

4-(6-bromo-1,2-dihydroacenaphthylen-5-yl)-N,N-diphenylaniline ***(TPA-ace-Br)*** *S8*

6-(4-(diphenylamino)phenyl)-1,2-dihydroacenaphthylene-5-carbonitrile ***(TPA-ace-CN)*** *S8*

4,4'-(1,2-dihydroacenaphthylene-5,6-diyl)bis(N,N-diphenylaniline) ***(TPA-ace-TRZ)*** *S9*

Spectra **S11**

Single crystal X-ray diffraction analysis **S32**

Cartesian coordinates of the ground-state optimized geometries **S39**

TPA-ace *S39*

2TPA-ace *S40*

TPA-ace-Br *S41*

TPA-ace-CN *S43*

TPA-ace-TRZ *S44*

Electrochemical properties **S46**

Photophysical properties **S46**

References **S56**

Experimental section

General synthetic procedures: All commercially available chemicals and reagent grade solvents were used as received. Air-sensitive reactions were performed using standard Schlenk techniques under a nitrogen atmosphere. Dry toluene was obtained from a solvent purification system. Flash column chromatography was carried out using silica gel (60 Å, 40-63 μm). Analytical thin-layer-chromatography (TLC) was performed with silica plates with plastic backings. TLC visualization was accomplished by 254/365 nm UV lamp. ^{1}H, and ^{13}C NMR spectra were recorded on a Bruker Avance NMR spectrometer in $CDCl_3$. Chemical shifts δ are expressed in parts per million (ppm) and referenced to chloroform (400 MHz for ^{1}H: $\delta = 7.26$ ppm, and 101 MHz for ^{13}C: $\delta = 77.16$ ppm). The signal structure is described as follows: s = singlet, d = doublet, m = multiplet, dd = doublet of doublets, dt = doublet of triplets, ddd = doublet of doublets of doublets. Melting points were measured using open-ended capillaries on an Electrothermal Mel-Temp® melting point apparatus and are uncorrected. High-resolution mass spectrometry (HRMS) was performed at the BSRC Mass Spectrometry Facility, University of St Andrews. Elemental analyses were performed by Mr. Stephen Boyer, London Metropolitan University. High performance liquid chromatography (HPLC) analysis was conducted on a Shimadzu Nexera prep HPLC system, using a Shimadzu Shim-pack GIST 3μm C18 analytical (3 X 150 mm) column.

Theoretical calculations: The DFT calculations, including geometry optimization of the emitters, were performed by the Gaussian 09 Revision D.01 software[1] in the gas phase using Density Functional Theory (DFT) level using PBE0 functional[2] and a 6-31G(d,p) basis set[3] starting with the molecular geometry obtained from single crystal X-ray diffraction analysis; that of **TPA-ace-Br** was optimized starting from an initial geometry drawn in GaussView v.5. Excited singlet and

triplet states were calculated by performing time dependent DFT (TD-DFT) calculations within the Tamm-Dancoff approximation[4] based on the same functional and basis set.

Electrochemical characterization: An electrochemical Analyzer potentiostat model 620E from CH Instruments was used for Cyclic Voltammetry (CV) analysis. All samples were prepared as dichloromethane (DCM) solutions, and bubbled with DCM saturated nitrogen gas for 15 minutes before measurements. 0.1 M DCM solution of tetra-*n*-butylammonium hexafluorophosphate was used as supporting electrolyte solution and Ag/Ag^+ electrode was used as the reference electrode while a platinum electrode and a platinum wire were used as the working electrode and counter electrode, respectively. The redox potentials are reported relative to a saturated calomel electrode (SCE) with a ferrocene/ferrocenium (Fc/Fc^+) redox couple as the internal standard (0.40 V vs SCE in DCM).[5]

Sample preparation: Steady state measurements in solution were performed in three different solvents: MCH, toluene and DCM. All solutions were prepared at concentration of 20 μM and degassed by five freeze−pump−thaw cycles. Solid state samples were fabricated by drop casting and spin coating method at 1% w/w of molecules in ZEONEX matrix (organic material 0.1mg/mL: ZEONEX 100 mg/mL – both dissolved in toluene). While drop-cast films were deposited onto quartz substrates, spin-coated films were deposited onto transparent sapphire substrates at 1200 rpm over a period of 60 seconds.

Photophysical characterization: Steady state measurements in solution were measured using a double beam Shimadzu UV-3600 UV/VIS/NIR spectrophotometer and a Horiba Jobin Yvon Fluorolog-3 spectrofluorometer. Time resolved measurements were detected by spectrograph and a gated iCCD camera (Stanford Computer Optics), where samples were excited with a Nd:YAG

laser (EKSPLA), 10 Hz, 355 nm. Photoluminescence quantum yields (PLQY) of spin coated films were measured using a calibrated integration sphere (Horiba Quanta-ϕ) coupled to a Horiba Fluorolog-3 spectrofluorometer. The integration sphere was purged for 30 minutes with dry nitrogen gas. PLQY values were obtained by exciting at 320 nm.

Synthesis

*5-Bromoacenaphthene (**Br-ace**)*

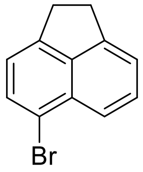

Br-ace was prepared according to the literature.[6] **Yield:** 90%. **Mp:** 54-56 °C (lit.[6] 50-52 °C). **^{1}H-NMR (ppm) δ:** 7.80 (dd, J = 8.4, 0.7 Hz, 1H), 7.68 (d, J = 7.3 Hz, 1H), 7.58 (dd, J = 8.3, 6.9 Hz, 1H), 7.35 (d, J = 6.9 Hz, 9H), 7.16 (dt, J = 7.3, 1.4 Hz, 1H), 3.47 – 3.41 (m, 2H), 3.36 (ddd, J = 5.5, 4.5, 2.0 Hz, 2H). **^{13}C NMR (ppm) δ:** 146.26, 145.97, 140.28, 130.89, 129.07, 121.76, 120.19, 120.07, 116.78, 30.65, 29.93. **HRMS (ESI) [M+H]$^{+}$ for ($C_{12}H_9Br$): Calculated:** 231.9888 [^{79}Br], 233.9868 [^{81}Br]; **Found:** 231.9885 [^{79}Br], 233.9868 [^{81}Br]. The ^{1}H NMR spectrum in $CDCl_3$ matches broadly with that previously reported in C_6D_6.[6]

*5,6-Dibromoacenaphthene (**DBr-ace**)*

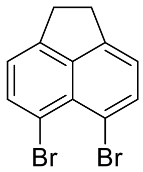

DBr-ace was prepared according to the literature.[7] **Yield:** 20-25%. **Mp:** 169-171 °C (lit.[7] 169-171 °C). **^{1}H NMR (ppm) δ:** 7.78 (d, J = 7.6 Hz, 2H), 7.07 (d, J = 7.6 Hz, 2H), 3.30 (s, 4H). **^{13}C NMR (ppm) δ:** 147.3, 142.16, 136.0, 128.0, 121.1, 114.6, 30.3. **HRMS (ESI) [M+H]$^{+}$ for ($C_{12}H_8Br_2$)**

Calculated: 310.9066 [^{79}Br, ^{79}Br], 312.9045 [^{79}Br, ^{81}Br], 314.9025 [^{81}Br, ^{81}Br]; **Found:** 310.9061 [^{79}Br, ^{79}Br], 312.9038 [^{79}Br, ^{81}Br], 314.9017 [^{81}Br, ^{81}Br]. The compound characterization is in agreement with that previously reported.[7]

General procedure for Suzuki-Miyaura cross-coupling reactions

A mixture of 4-(*N,N*-diphenylamino)phenyl boronic acid (1 equiv.), the bromo derivative of acenaphthene (1-2 equiv.), and $Pd(PPh_3)_4$ (5 mol%) in toluene (10 mL) was heated to 90 ^{o}C and stirred for 30 min under a N_2 atmosphere. A de-aerated aqueous solution of K_2CO_3 (2M, 0.1 mL) was injected into the reaction and the mixture was refluxed for 16 h. After cooling, the reaction mixture was diluted with ethyl acetate and filtered through Celite®. The filtrate was washed with brine, dried over anhydrous sodium sulfate and the solvent removed under reduced pressure. The residue was purified by flash column chromatography (silica gel, eluent: 5% EtOAc/hexane or 20% DCM/hexane).

*4-(1,2-dihydroacenaphthylen-5-yl)-N,N-diphenylaniline (**TPA-ace**)*

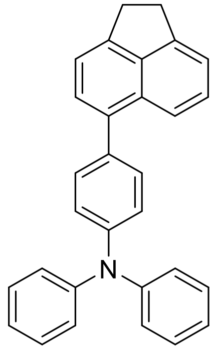

TPA-ace: Yellow solid. **R*f*:** 0.4 (5% EA/Hexanes). **Yield:** 0.10 g, 11%. **Mp:** 108-110 ^{o}C. **^{1}H NMR (ppm) δ:** 7.82 (d, *J* = 8.2 Hz, 1H), 7.47 (ddd, *J* = 6.9, 4.0, 1.3 Hz, 4H), 7.38 – 7.26 (m, 8H), 7.24 – 7.19 (m, 6H), 7.19 – 7.12 (m, 2H), 7.11 – 7.00 (m, 3H), 3.59 – 3.34 (m, 4H). **^{13}C NMR (ppm) δ:** 147.84, 146.78, 146.22, 145.28, 139.64, 135.26, 134.54, 130.49, 129.77, 129.28, 128.36,

127.90, 124.42, 123.62, 122.84, 120.96, 119.31, 119.17, 30.57, 30.01. **HRMS (ESI) $[M+H]^+$ for ($C_{30}H_{23}N$) Calculated:** 398.1909; **Found:** 398.1903. **Elemental analysis for ($C_{30}H_{23}N$) Calculated:** C, 90.64; H, 5.83; N, 3.52. **Found:** C, 90.60; H, 5.66; N, 3.67. **HPLC** (RT): 2.451 min (100% methanol).

4,4'-(1,2-dihydroacenaphthylene-5,6-diyl)bis(N,N-diphenylaniline) ***(2TPA-ace)***

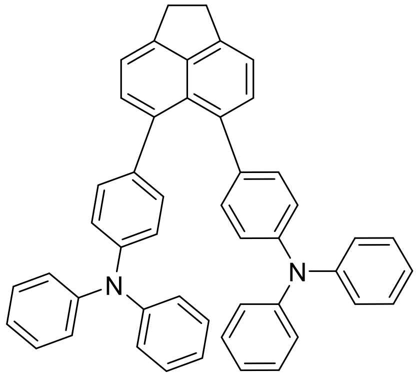

2TPA-ace: Orange solid. **R_f:** 0.3 (20% DCM/Hexanes). **Yield:** 0.25 g, 24%. **Mp:** 242-244 °C. **^{1}H NMR (ppm) δ:** 7.42 (dd, J = 17.1, 7.1 Hz, 4H), 7.26 – 7.18 (m, 16H), 7.06 – 6.99 (m, 4H), 6.92 – 6.80 (m, 4H), 3.51 (s, 4H). **^{13}C NMR (ppm) δ:** 147.88, 145.73, 145.66, 140.73, 136.49, 136.04, 131.78, 130.47, 129.26, 127.50, 124.99, 122.76, 121.33, 119.24, 30.22. **HRMS (ESI) $[M+H]^+$ for ($C_{48}H_{36}N_2$) Calculated:** 641.2957; **Found:** 641.2961. **Elemental Analysis for ($C_{48}H_{36}N_2$) Calculated:** C, 89.97; H, 5.66; N, 4.37. **Found:** C, 90.19; H, 5.77; N, 4.48. **HPLC** (RT): 7.850 min (95% methanol).

*4-(6-bromo-1,2-dihydroacenaphthylen-5-yl)-N,N-diphenylaniline **(TPA-ace-Br)***

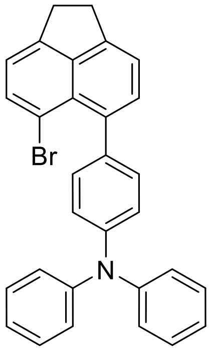

TPA-ace-Br: Bright yellow solid. **R_f:** 0.6 (20% DCM/Hexanes). **Yield:** 0.2 g, 21%. **Mp:** 154-156 °C. **^{1}H NMR (ppm) δ:** 7.72 (d, *J* = 7.3 Hz, 1H), 7.45 (d, *J* = 7.1 Hz, 1H), 7.38 – 7.27 (m, 5H), 7.27 – 7.18 (m, 6H), 7.18 – 7.11 (m, 3H), 7.08 – 7.01 (m, 2H), 3.49 – 3.44 (m, 2H), 3.43 – 3.38 (m, 2H). **^{13}C NMR (ppm) δ:** 147.94, 146.69, 146.47, 146.20, 141.11, 136.11, 135.91, 134.38, 132.21, 131.50, 129.22, 128.40, 124.20, 122.93, 122.58, 120.33, 119.45, 114.90, 30.29, 30.03. **HRMS (ESI) $[M+H]^+$ for ($C_{30}H_{22}BrN$) Calculated:** 476.1008 [^{79}Br], 478. 0988 [^{81}Br]. **Found:** 476.1003 [^{79}Br], 478. 0983 [^{81}Br]. **Elemental analysis for ($C_{30}H_{22}BrN$) Calculated:** C, 75.63; H, 4.65; N, 2.94. **Found:** C, 75.87; H, 4.85; N, 2.88. **HPLC** (RT): 7.440 min (95% methanol).

*6-(4-(diphenylamino)phenyl)-1,2-dihydroacenaphthylene-5-carbonitrile **(TPA-ace-CN)***

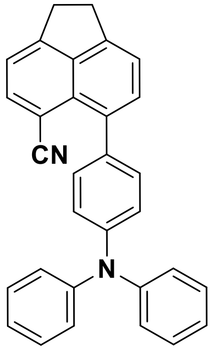

A mixture of **TPA-ace-Br** (0.9 g, 1.9 mmol, 1 equiv.) and CuCN (0.5 g, 5.6 mmol, 3 equiv.) in NMP (10 mL) was heated to 160 °C for 6 h. After cooling to room temperature, the reaction mixture was diluted with water and extracted with DCM (3 × 50 mL). The aqueous phase was treated with saturated NaOCl solution to destroy unreacted CuCN. The organic phase was washed with brine, dried over anhydrous sodium sulfate and the solvent was removed under reduced pressure. The crude product was purified by flash column chromatography with 30% DCM/hexane as eluent to afford the title compound as an orange solid. **R_f:** 0.5 (30% DCM/hexanes). **Yield:** 0.65 g, 81%. **Mp:** 155-157 °C. **^{1}H NMR (ppm) δ:** 7.89 (d, *J* = 7.2 Hz, 1H), 7.54 (d, *J* = 7.1 Hz, 1H), 7.46 (d, *J* = 7.1 Hz, 1H), 7.36 (d, *J* = 7.2 Hz, 1H), 7.34 – 7.15 (m, 14H), 7.03 (s, 2H), 3.51 (s, 4H). **^{13}C NMR (ppm) δ:** 152.81, 146.16, 139.33, 137.95, 131.27, 129.20, 124.44, 123.43, 122.68, 120.53, 119.10, 118.61, 104.69, 31.00, 29.99. **HRMS (ESI) Calculated (m/z):** 422.1783**; Found:** 422.1769**. Elemental analysis (calculated for $C_{31}H_{22}N_2$):** C, 88.12; H, 5.25; N, 6.63; **Found:** C, 88.00; H, 5.35; N, 6.52. **HPLC** (RT): 1.584 min (100% methanol).

4,4'-(1,2-dihydroacenaphthylene-5,6-diyl)bis(N,N-diphenylaniline) ***(TPA-ace-TRZ)***

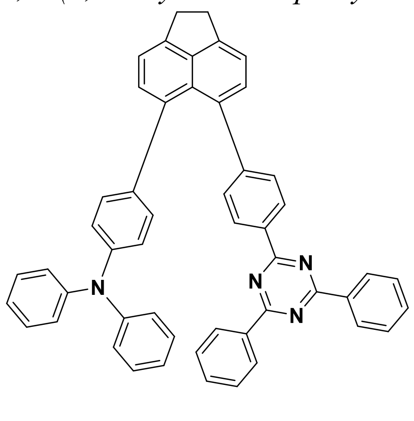

A reaction mixture of **TPA-ace-Br** (0.20 g, 1 equiv., 0.42 mmol) and $Pd(PPh_3)_4$ (24 mg, 0.05 equiv., 21 μmol) in toluene (6 mL) was heated to 90 °C for 15 min under a N_2 atmosphere before

addition of 2,4-diphenyl-6-(4-(4,4,5,5-tetramethyl-1,3,2-dioxaborolan-2-yl)phenyl)-1,3,5-triazine (0.18 g, 1 equiv., 0.42 mmol). A solution of potassium carbonate (2 M, 4 mL) in water : ethanol (1:1) was deoxygenated by bubbling nitrogen gas for 15 min and added to the reaction mixture, which was then heated to 100 °C for 24 h. After cooling to room temperature, the reaction mixture was diluted with DCM (50 mL) and washed with brine (1 × 30 mL). The organic layer was dried over anhydrous sodium sulphate and the solvent was removed under reduced pressure. The crude product was purified by flash column chromatography with 20% DCM/hexanes to afford the title compound as an orange solid. **R_f:** 0.5 (20% DCM/hexane). **Yield:** 0.1 g, 34%. **Mp:** 249-250 °C. **^{1}H NMR (ppm) δ:** 8.87 – 8.79 (m, 4H), 8.60 – 8.54 (m, 2H), 7.71 – 7.58 (m, 6H), 7.52 – 7.42 (m, 4H), 7.34 – 7.26 (m, 4H), 7.01 – 6.90 (m, 6H), 6.85 – 6.78 (m, 4H), 6.78 – 6.70 (m, 2H), 6.69 – 6.60 (m, 2H), 3.55 (s, 4H). **^{13}C NMR (ppm) δ:** 171.59, 147.62, 147.40, 146.76, 145.71, 140.78, 136.39, 135.94, 135.84, 135.57, 133.40, 132.47, 132.36, 132.07, 130.18, 130.09, 129.06, 128.99, 128.65, 127.84, 127.44, 124.69, 122.69, 121.23, 119.58, 119.25, 30.31, 30.23. **HRMS (ESI) $[M+H]^+$ calculated:** 705.3013; **Found:** 705.3006**. Elemental analysis (calculated for $C_{51}H_{36}N_4$):** C, 86.90; H, 5.15; N, 7.95; **Found:** C, 86.76; H, 5.06; N, 8.10. **HPLC** (RT): 8.623 min (100% methanol).

Spectra

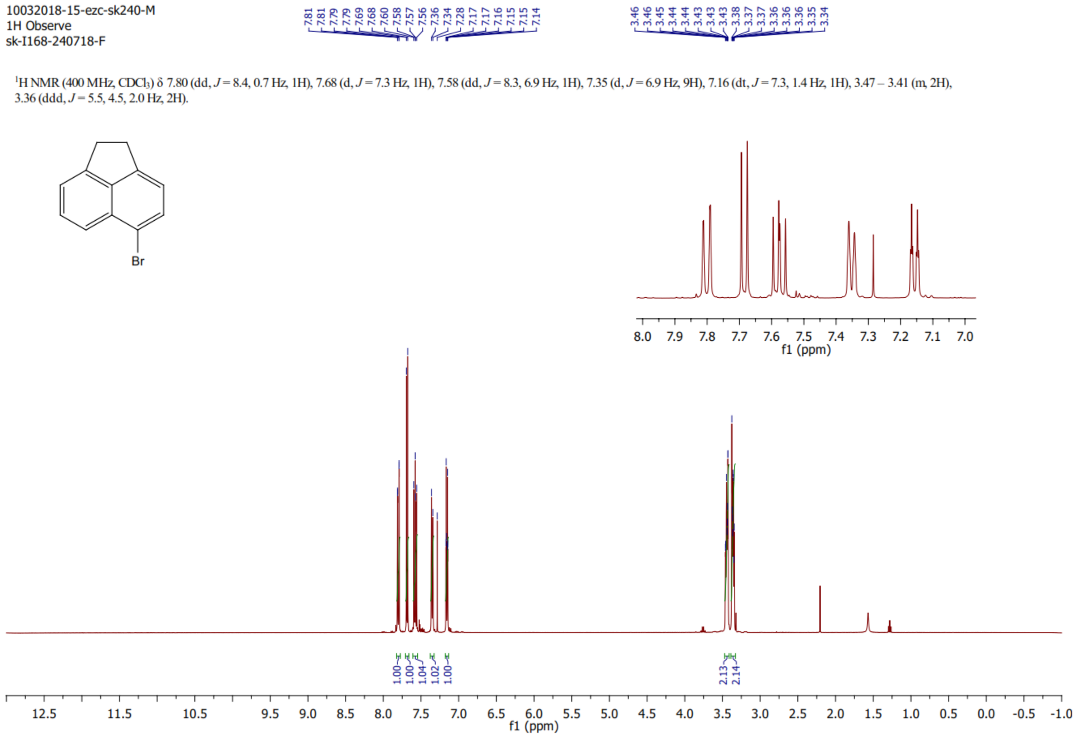

Figure **S1**. ^{1}H NMR spectra of **Br-ace.**

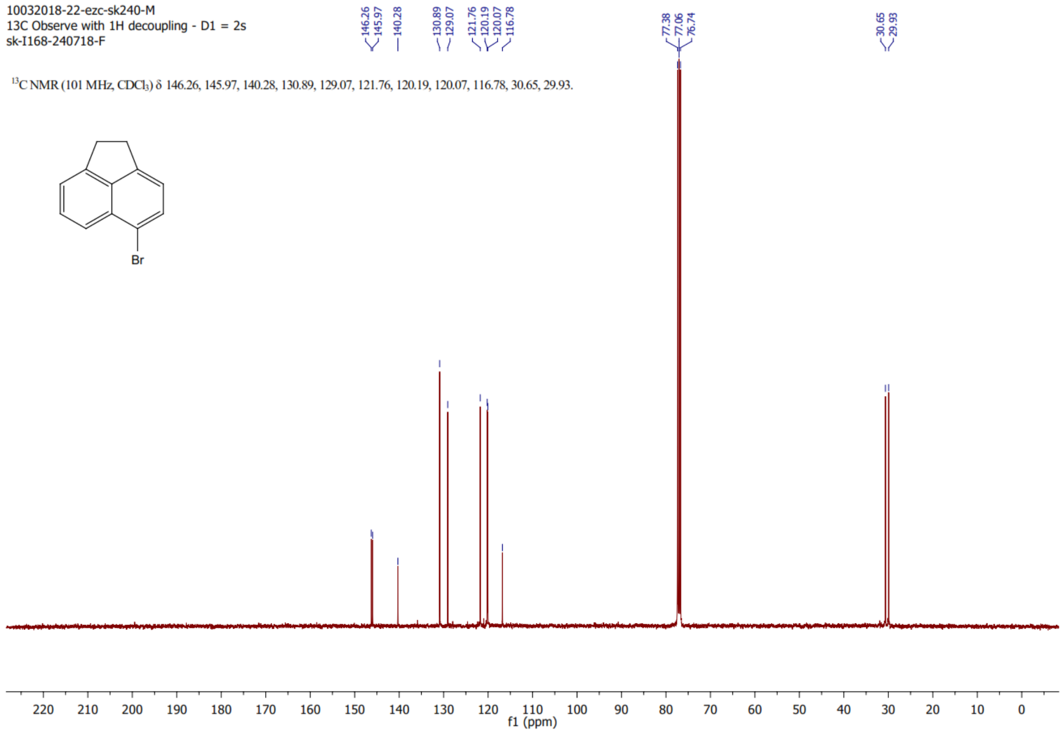

Figure **S2**. ^{13}C NMR spectra of **Br-ace.**

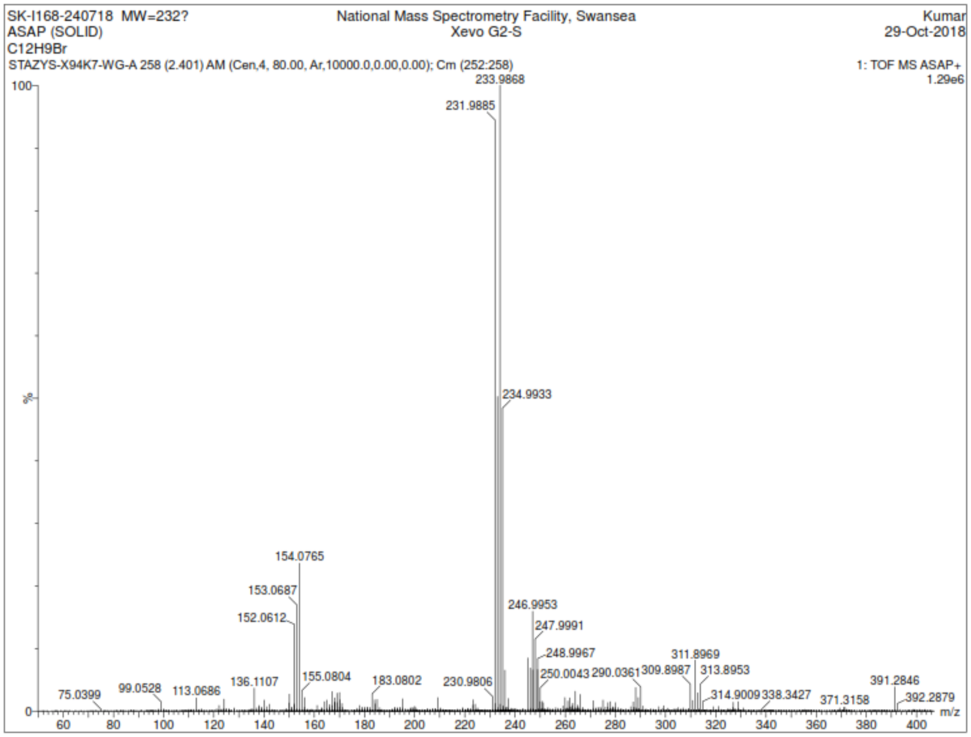

Figure **S3**. HRMS spectra of **Br-ace.**

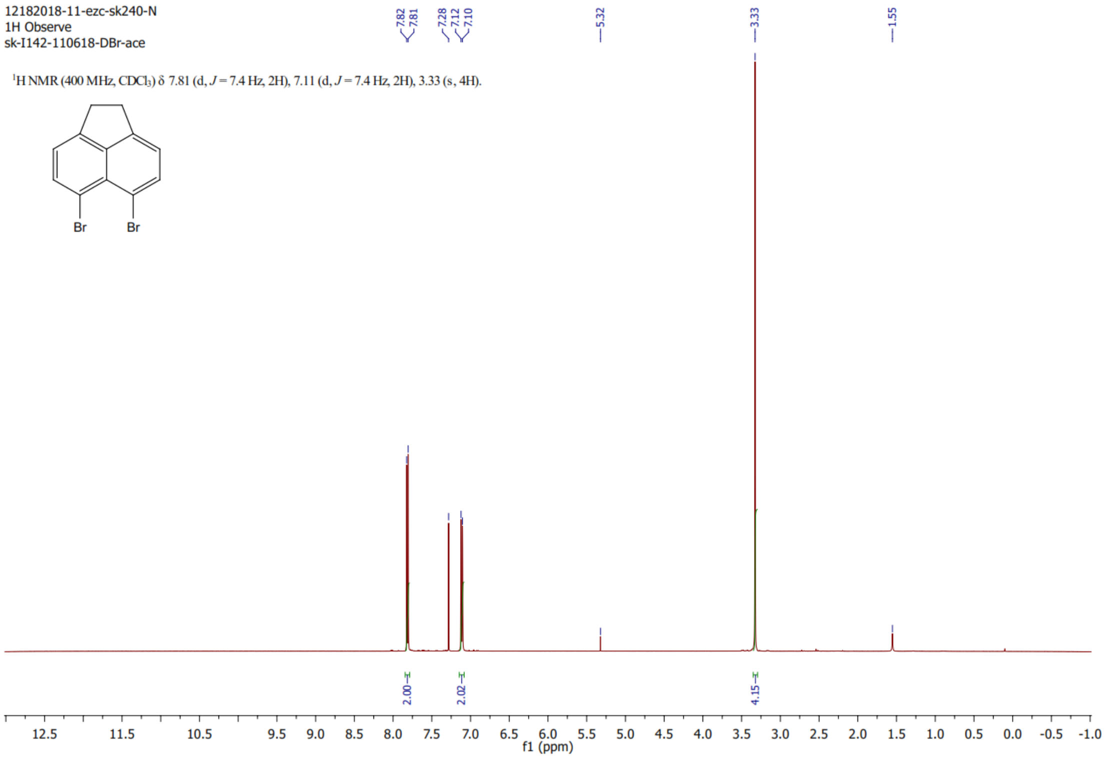

Figure **S4**. ^{1}H NMR spectra of **DBr-ace.**

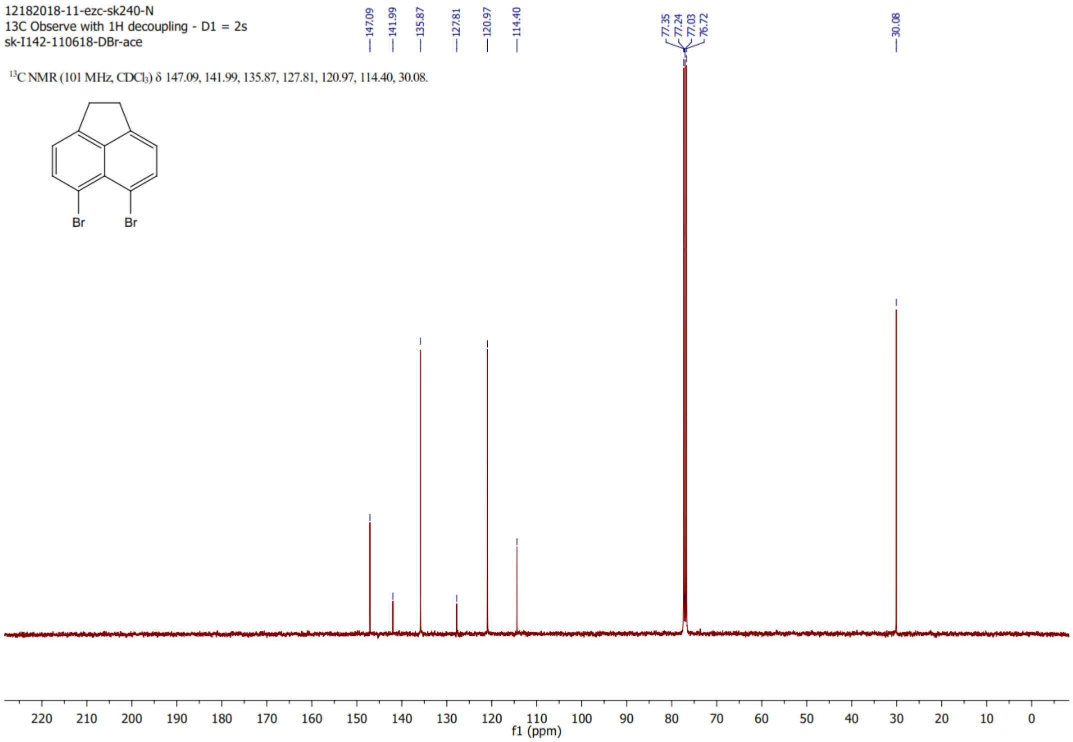

Figure **S5**. ^{13}C NMR spectra of **DBr-ace.**

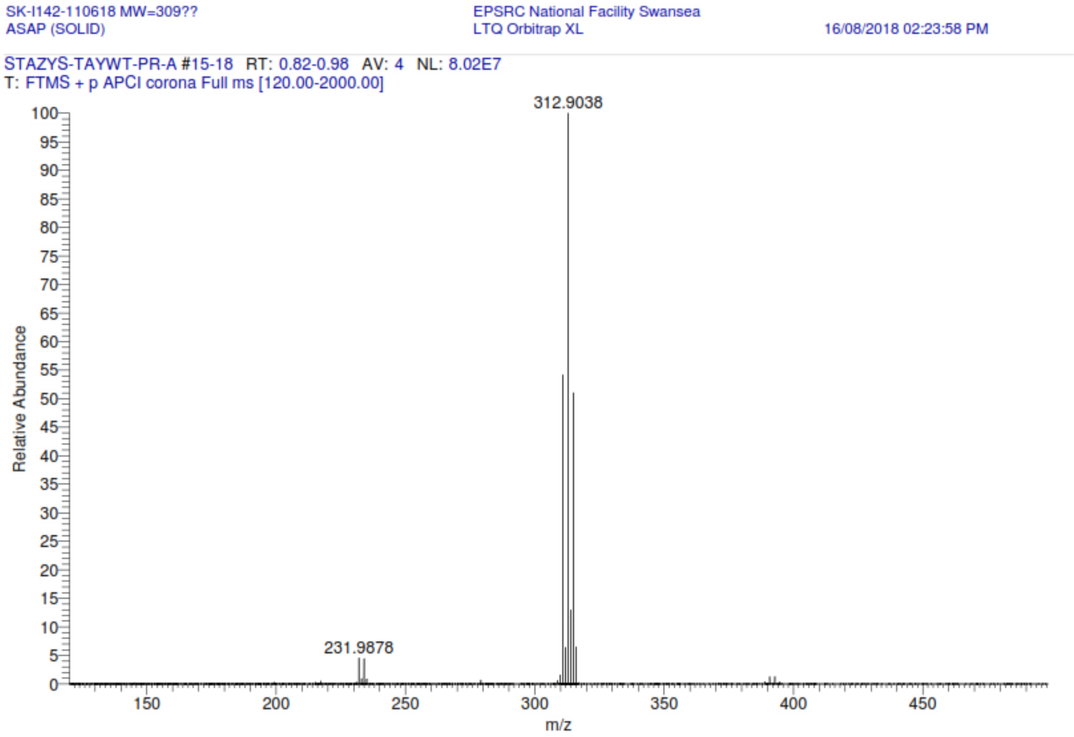

Figure **S6**. HRMS spectra of **DBr-ace.**

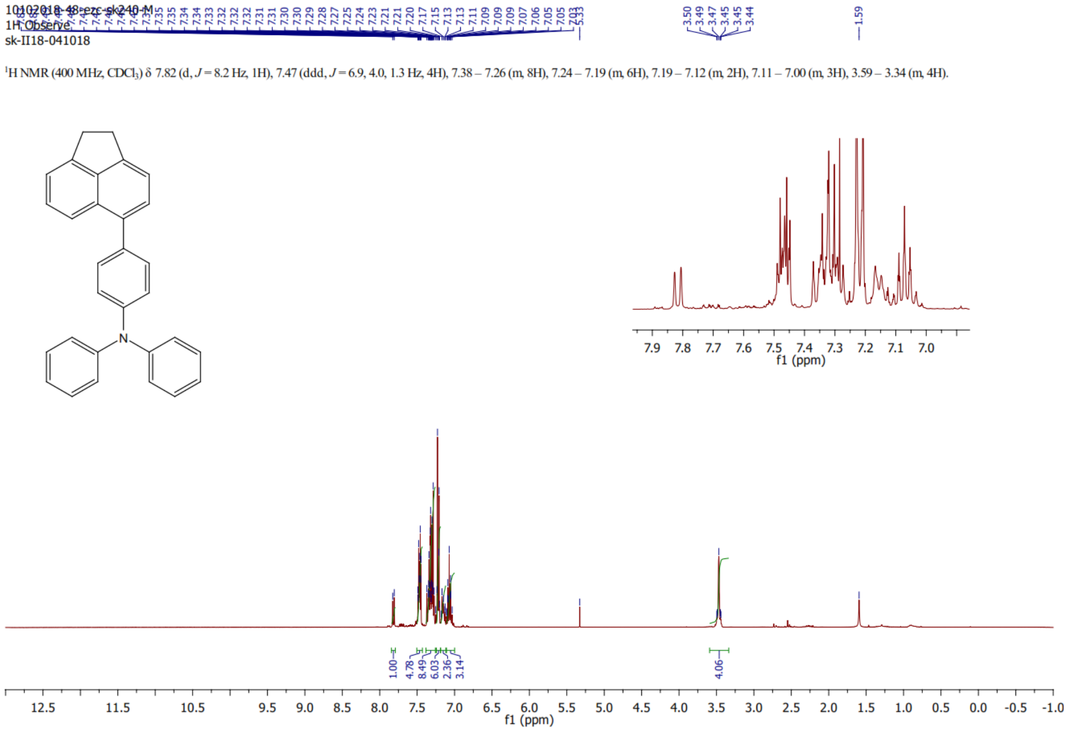

Figure **S7**. ^{1}H NMR spectra of **TPA-ace.**

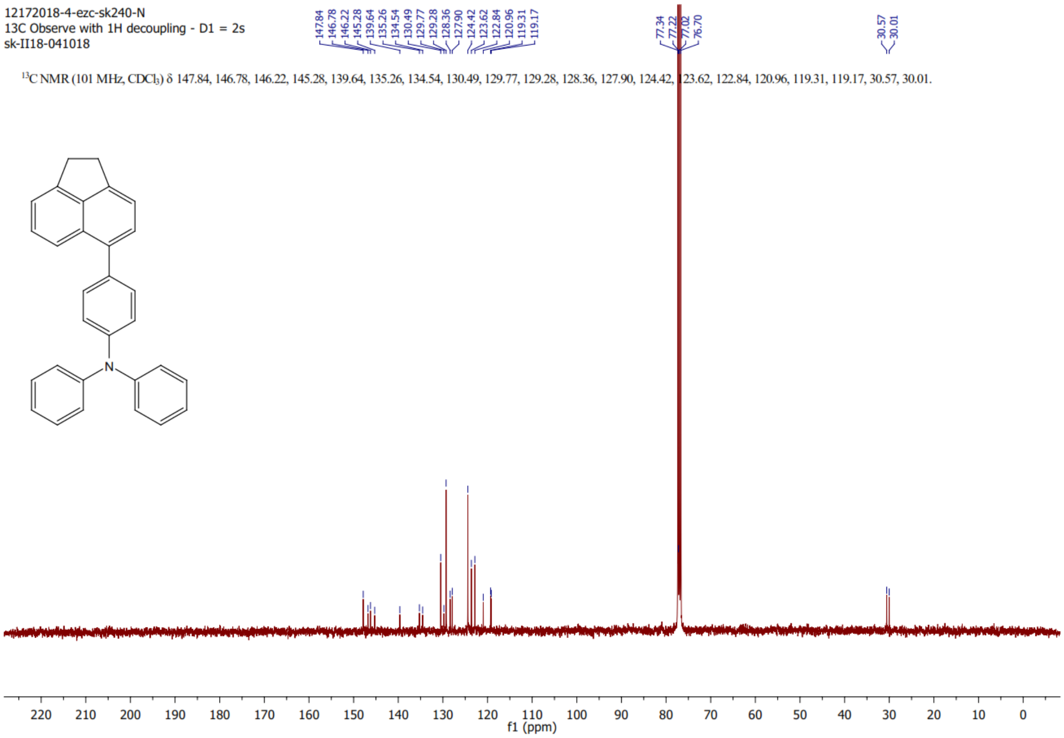

Figure **S8**. ^{13}C NMR spectra of **TPA-ace.**

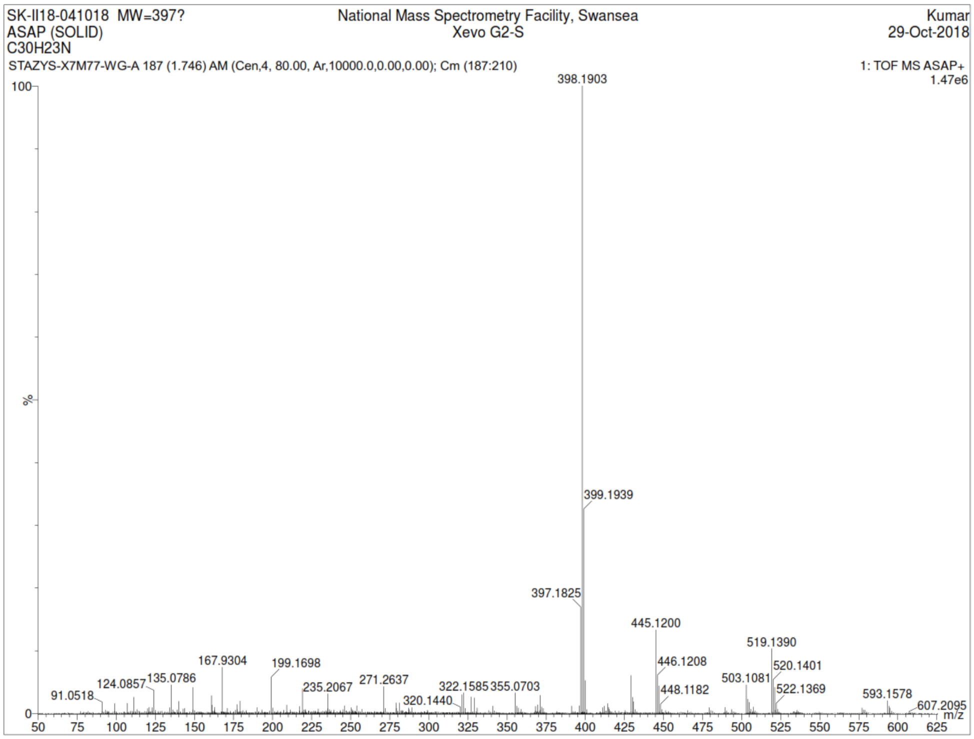

Figure **S9**. HRMS spectra of **TPA-ace.**

LONDON metropolitan university

Elemental Analysis Service

Please send completed form and samples to:

Stephen Boyer
School of Human Sciences
Science Centre
London Metropolitan University
29 Hornsey Road
London N7 7DD

Telephone: 020 7133 3605
Fax: 020 7133 2577
Email: s.boyer@londonmet.ac.uk

Sample submitted by:	Dr. Shiv Kumar
Address:	EZC Group, School of Chemistry, University of St Andrews.
Telephone: 01334 467243	Email: sk240@st-andrews.ac.uk
Date Submitted:	16-01-2019

Please submit ca. 5 mg of sample.

Sample Reference No.:	SK-II 18-041018
Name of Compound:	TPA-Ace
Molecular Formula:	$C_{30}H_{23}N$
Stability:	Stable at rt & pressure
Hazards:	Unknown
Other Remarks:	

Element	Expected %	Found (1)	Found (2)	
Carbon	90.64	90.68	90.60	
Hydrogen	5.83	5.59	5.66	
Nitrogen	3.52	3.68	3.67	

Authorising Signature:

Date Completed: 2301[illegible]	Signature:
Comments:	

Figure **S10**. Elemental analysis report for **TPA-ace**.

HPLC Trace Report25Nov2020

<Sample Information>

Sample Name	: TPA-ace		
Sample ID	: SK-II18-041018		
Method Filename	: 100% Methanol B 10 mins.lcm		
Batch Filename	: acenaphthene.lcb		
Vial #	: 1-21	Sample Type	: Unknown
Injection Volume	: 10 uL		
Date Acquired	: 10/03/2020 18:49:43	Acquired by	: System Administrator
Date Processed	: 10/03/2020 19:09:53	Processed by	: System Administrator

<Chromatogram>

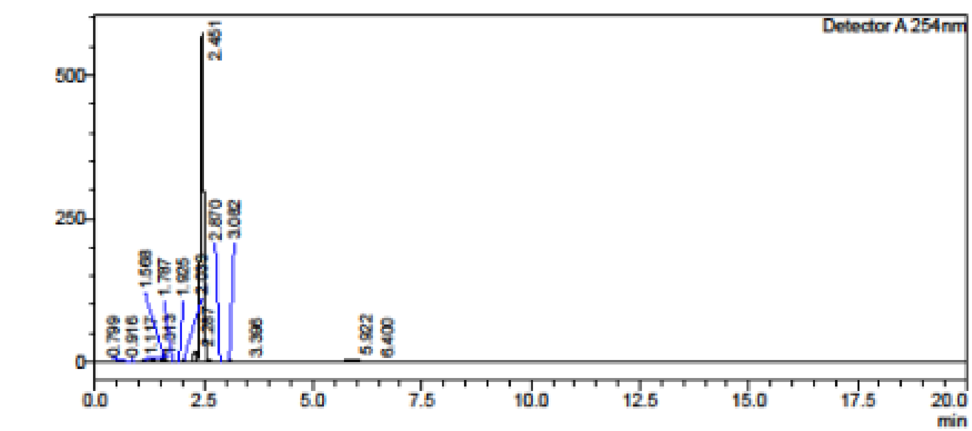

<Peak Table>

Detector A 254nm

Peak#	Ret. Time	Area	Height	Area%	Area/Height	Width at 5% Height
1	0.799	9751	1219	0.294	7.999	--
2	0.916	7245	1435	0.219	5.049	--
3	1.117	28850	2970	0.871	9.714	--
4	1.313	33759	5657	1.019	5.968	--
5	1.568	64157	13810	1.936	4.646	0.162
6	1.787	3811	544	0.115	7.005	--
7	1.925	3381	491	0.102	6.888	--
8	2.033	20497	4068	0.619	5.038	--
9	2.287	93233	19439	2.814	4.796	--
10	2.451	2949721	567292	89.032	5.200	0.169
11	2.870	2239	479	0.068	4.677	0.134
12	3.082	14049	2375	0.424	5.916	0.192
13	3.396	1572	266	0.047	5.916	0.186
14	5.922	79231	5010	2.391	15.815	0.537
15	6.400	1614	125	0.049	12.914	--
Total		3313109	625179	100.000		

Figure **S11**. HPLC analysis report for **TPA-ace**.

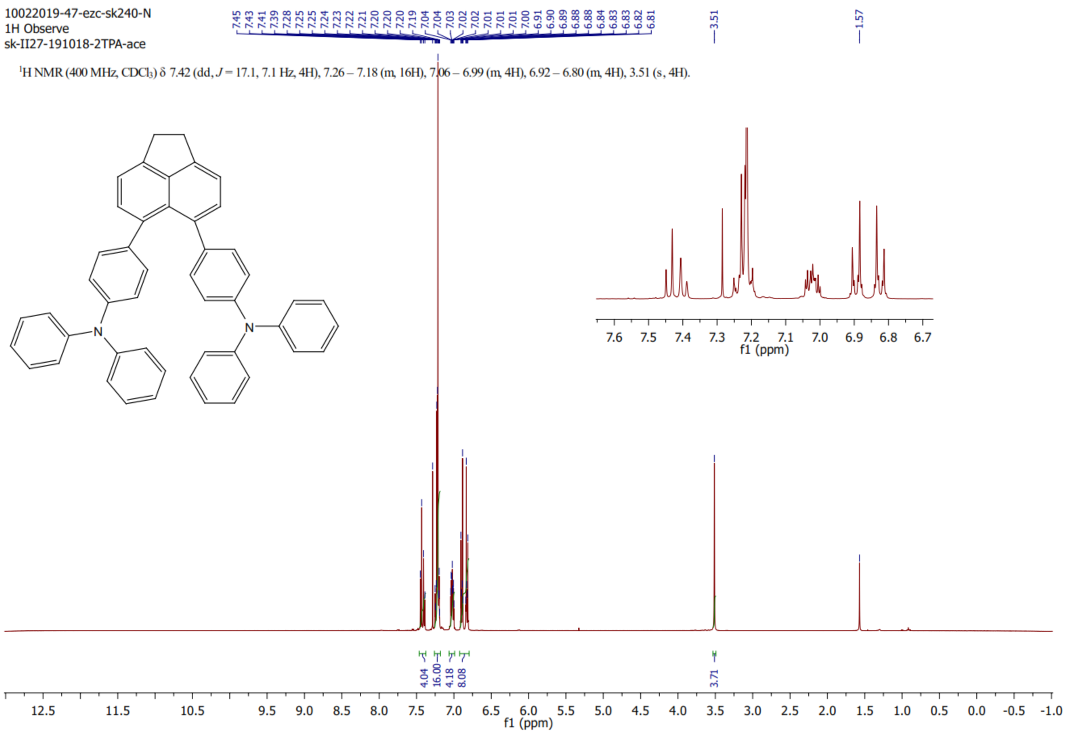

Figure **S12**. ^{1}H NMR spectra of **2TPA-ace.**

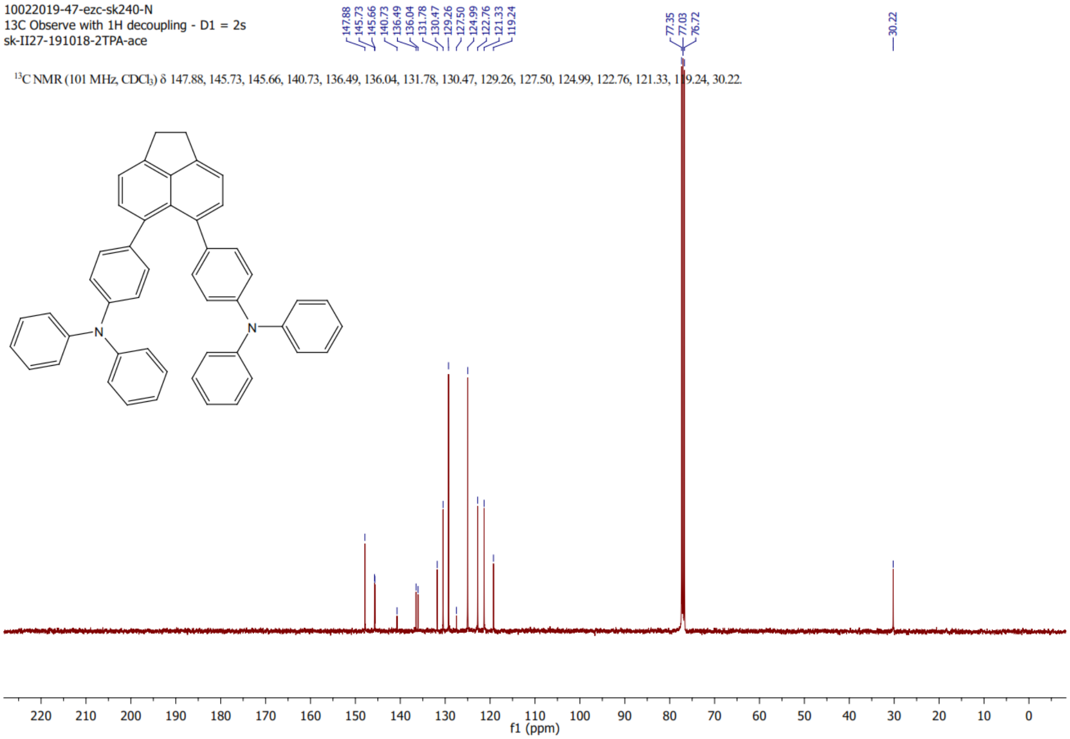

Figure **S13**. ^{13}C NMR spectra of **2TPA-ace.**

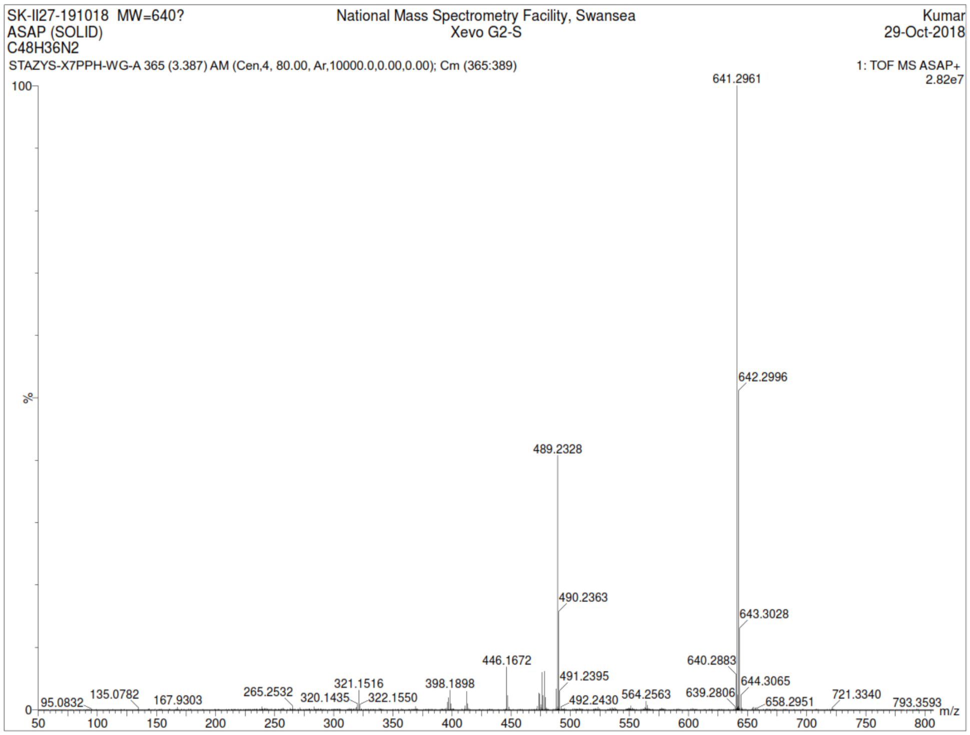

Figure **S14**. HRMS spectra of **2TPA-ace.**

LONDON metropolitan university

Elemental Analysis Service

Please send completed form and samples to:

Stephen Boyer
School of Human Sciences
Science Centre
London Metropolitan University
29 Hornsey Road
London N7 7DD

Telephone: 020 7133 3605
Fax: 020 7133 2577
Email: s.boyer@londonmet.ac.uk

Sample submitted by:	Dr Shiv Kumar
Address:	EZC Group, School of Chemistry, University of St Andrews
Telephone:	01334 467243 Email: sk240@st-andrews.ac.uk
Date Submitted:	16-01-2019

Please submit ca. 5 mg of sample.

Sample Reference No.:	SK-II27-191018
Name of Compound:	2TPA-Ace
Molecular Formula:	$C_{48}H_{36}N_2$
Stability:	Stable at rt and pressure
Hazards:	N/A (Unknown)
Other Remarks:	

Element	Expected %	Found (1)	Found (2)	
Carbon	89.97	90.19	90.11	
Hydrogen	5.66	5.77	5.78	
Nitrogen	4.37	4.45	4.45	

Authorising Signature:

Date Completed: 220119	Signature: [illegible]
Comments:	

Figure **S15**. Elemental analysis report for **2TPA-ace**.

HPLC Trace Report25Nov2020

<Sample Information>

Sample Name	: 2TPA-ace		
Sample ID	: SK-II27-191018		
Method Filename	: 95% Methanol 5 Water 20 mins.lcm		
Batch Filename	: acenaphthene_5.lcb		
Vial #	: 1-36	Sample Type	: Unknown
Injection Volume	: 10 uL		
Date Acquired	: 13/03/2020 18:35:58	Acquired by	: System Administrator
Date Processed	: 13/03/2020 19:01:08	Processed by	: System Administrator

<Chromatogram>

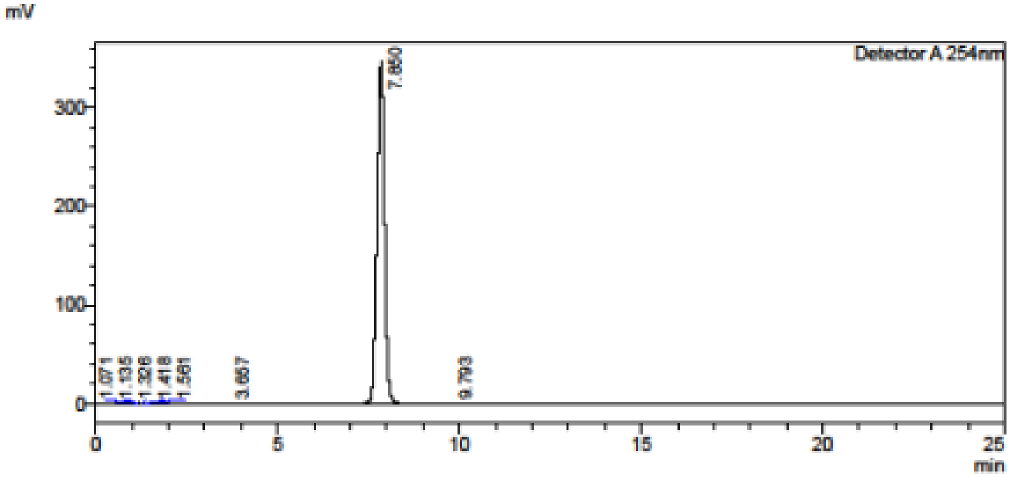

<Peak Table>

Detector A 254nm

Peak#	Ret. Time	Area	Height	Area%	Area/Height	Width at 5% Height
1	1.071	5914	924	0.119	6.401	--
2	1.135	7708	1064	0.155	7.247	--
3	1.326	4374	816	0.088	5.363	--
4	1.418	3308	636	0.066	5.201	--
5	1.561	11264	1356	0.226	8.306	--
6	3.657	1407	151	0.028	9.327	0.286
7	7.850	4936633	345409	99.232	14.292	0.477
8	9.793	4255	221	0.086	19.261	0.553
Total		4974863	350576	100.000		

Figure **S16**. HPLC analysis of **2TPA-ace**.

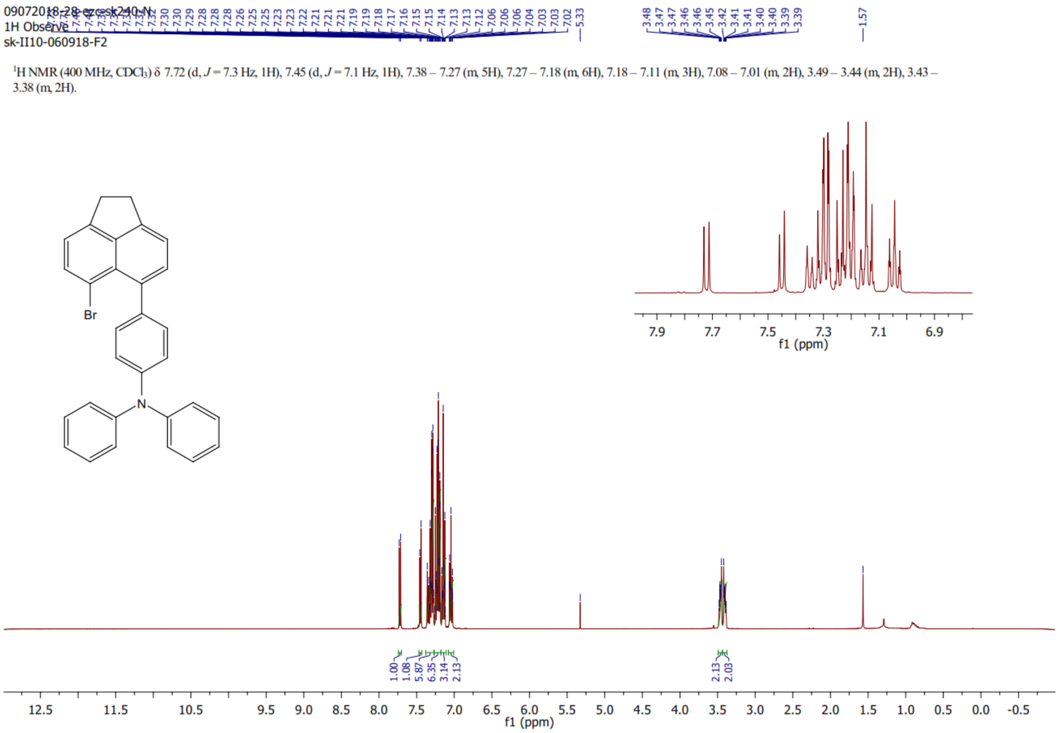

Figure **S17**. ^{1}H NMR spectra of **TPA-ace-Br.**

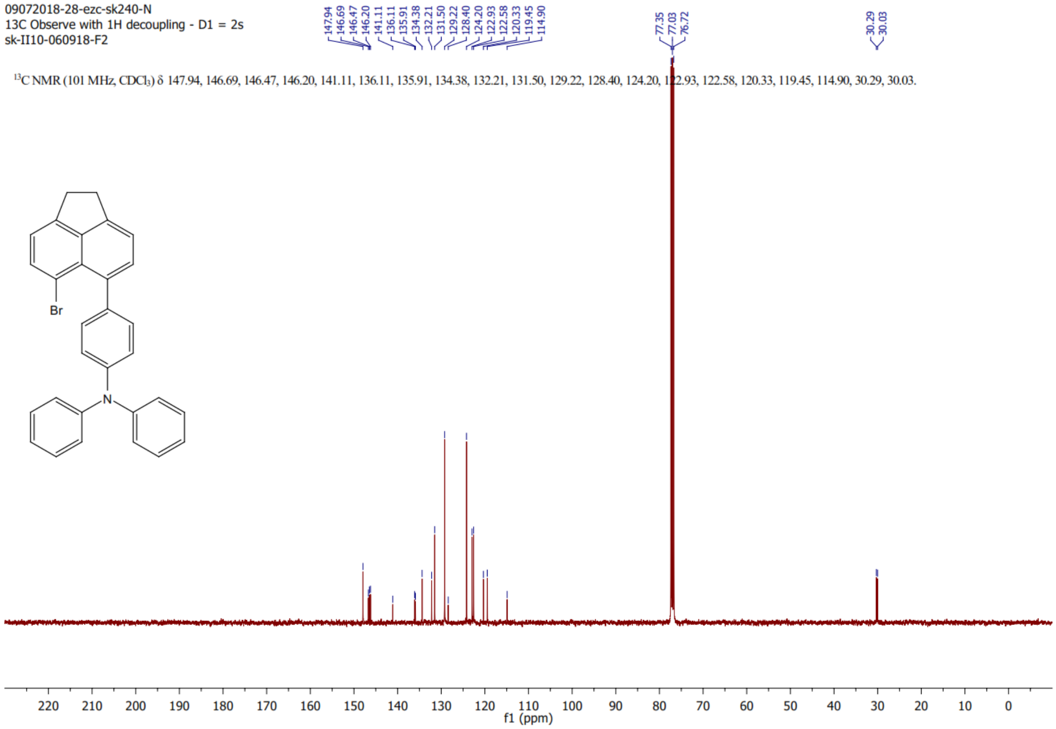

Figure **S18**. ^{13}C NMR spectra of **TPA-ace-Br.**

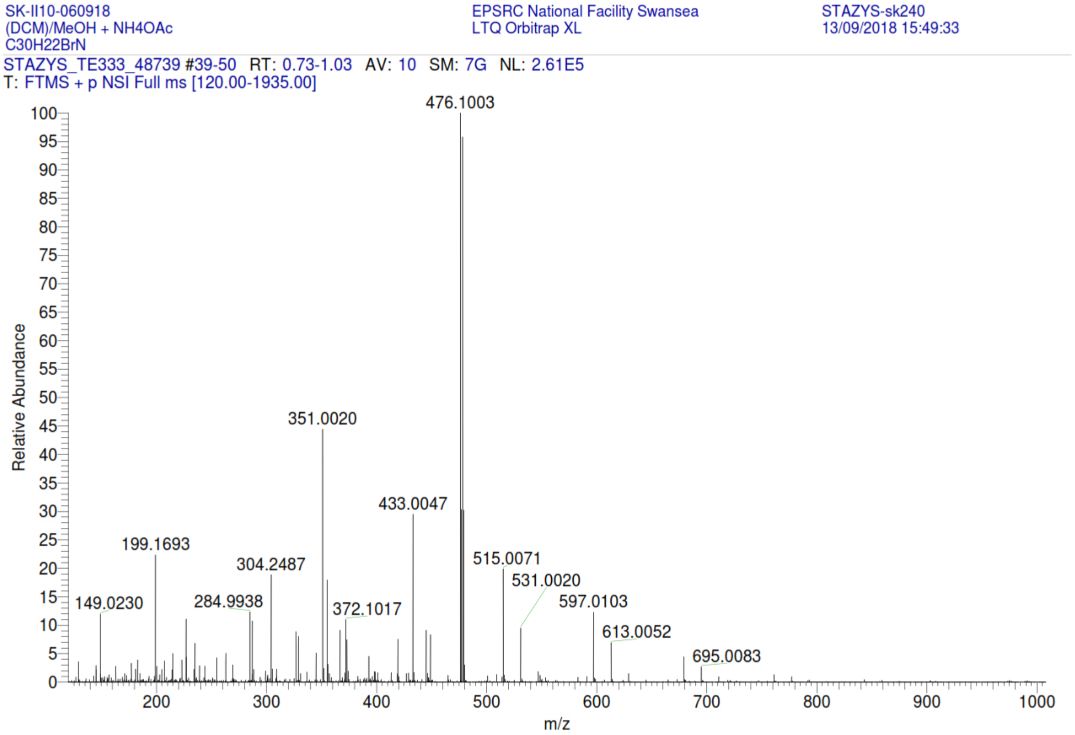

Figure **S19**. HRMS spectra of **TPA-ace-Br.**

LONDON metropolitan university

Elemental Analysis Service

Please send completed form and samples to:

Stephen Boyer
School of Human Sciences
Science Centre
London Metropolitan University
29 Hornsey Road
London N7 7DD

Telephone: 020 7133 3605
Fax: 020 7133 2577
Email: s.boyer@londonmet.ac.uk

Sample submitted by: Dr. Shiv Kumar	
Address: EZC Group, School of Chemistry, Univ. of St Andrews	
Telephone: 01334 467243	Email: sk240@st-andrews.ac.uk
Date Submitted: 11-09-2018	

Please submit ca. 5 mg of sample.

Sample Reference No.: SK-II 10 -060918
Name of Compound: 4-(6-bromo-1,2-dihydroacenaphthylen-5-yl)-N,N-diphenylaniline
Molecular Formula: $C_{30}H_{22}BrN$
Stability: Stable at r.t. & pressure
Hazards: None
Other Remarks:

Element	Expected %	Found (1)	Found (2)	
Carbon	75.63	75.44	75.52	
Hydrogen	4.65	4.66	4.68	
Nitrogen	2.94	3.05	3.08	

Authorising Signature:

Date Completed: 170918	Signature: [illegible]
Comments:	

Figure **S20**. Elemental analysis for **TPA-ace-Br**.

HPLC Trace Report24Nov2020

<Sample Information>

Sample Name	: TPA-ace-Br		
Sample ID	:		
Method Filename	: 87% Acetonitrile 13 Water 20 mins.lcm		
Batch Filename	: Shiv_TPA-ace_series-MeCN-87_TPA-ace-Br-97_TPA-ace-TRZ.lcb		
Vial #	: 2-47	Sample Type	: Unknown
Injection Volume	: 2 uL		
Date Acquired	: 20/11/2020 09:02:25	Acquired by	: System Administrator
Date Processed	: 20/11/2020 09:22:28	Processed by	: System Administrator

<Chromatogram>

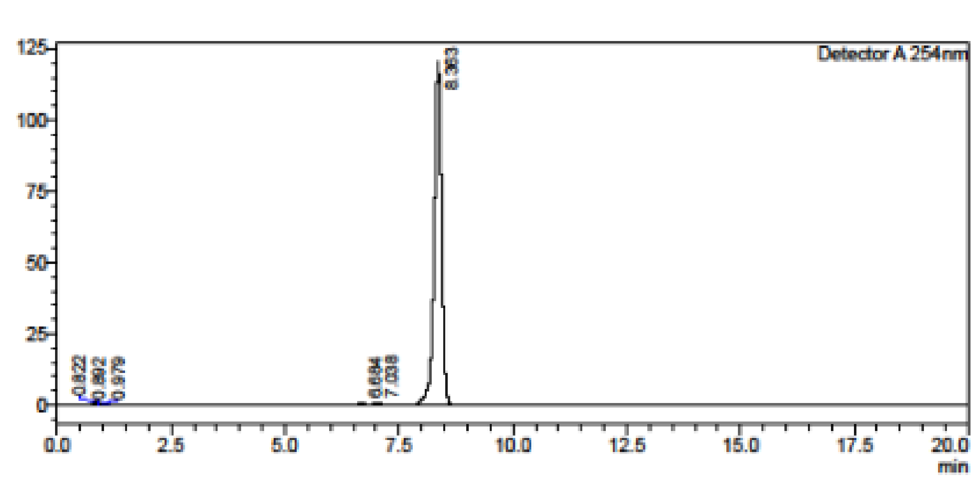

<Peak Table>

Detector A 254nm

Peak#	Ret. Time	Area	Height	Area%	Area/Height	Width at 5% Height
1	0.822	4698	1448	0.332	3.245	--
2	0.892	1051	397	0.074	2.646	--
3	0.979	2742	569	0.194	4.823	--
4	6.684	5863	665	0.414	8.816	--
5	7.038	9859	1059	0.696	9.307	0.309
6	8.363	1391954	120124	98.290	11.588	0.411
Total		1416167	124262	100.000		

Figure **S21**. HPLC analysis report for **TPA-ace-Br**.

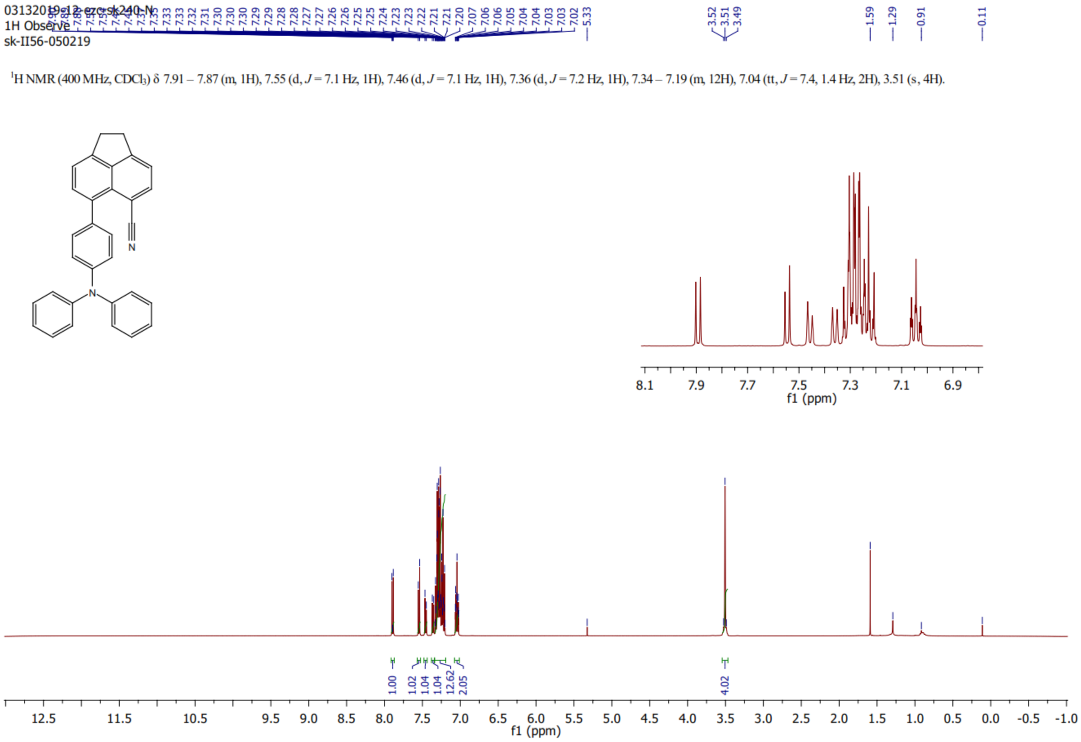

Figure **S22**. ^{1}H NMR spectra of **TPA-ace-CN.**

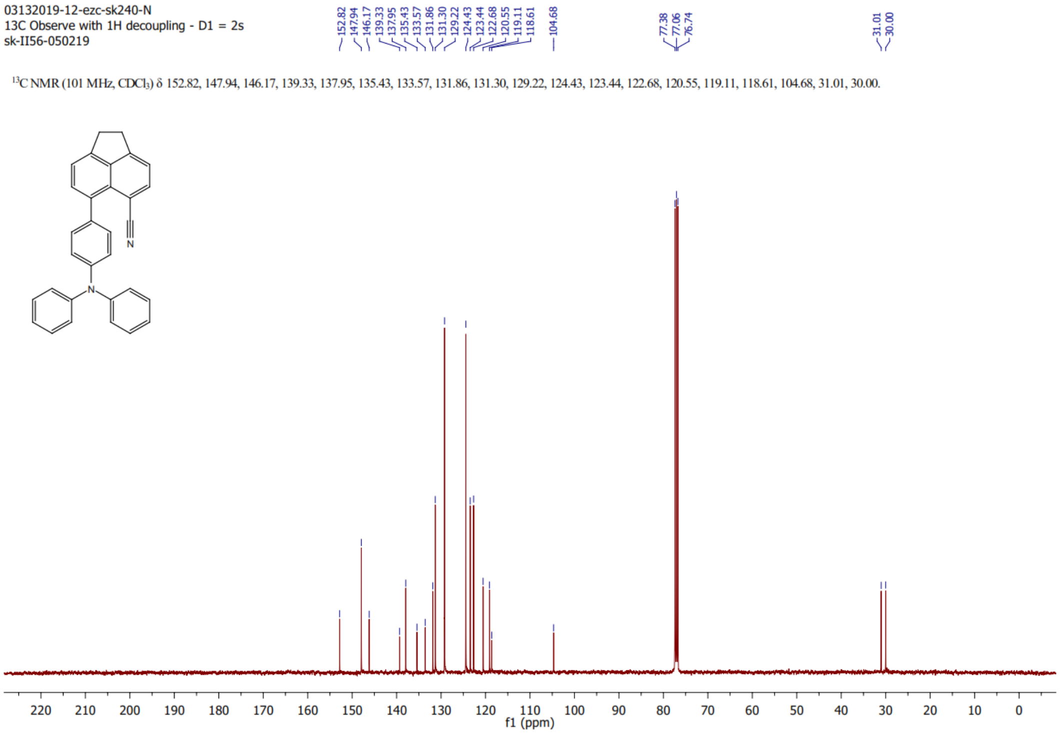

Figure **S23**. ^{13}C NMR spectra of **TPA-ace-CN.**

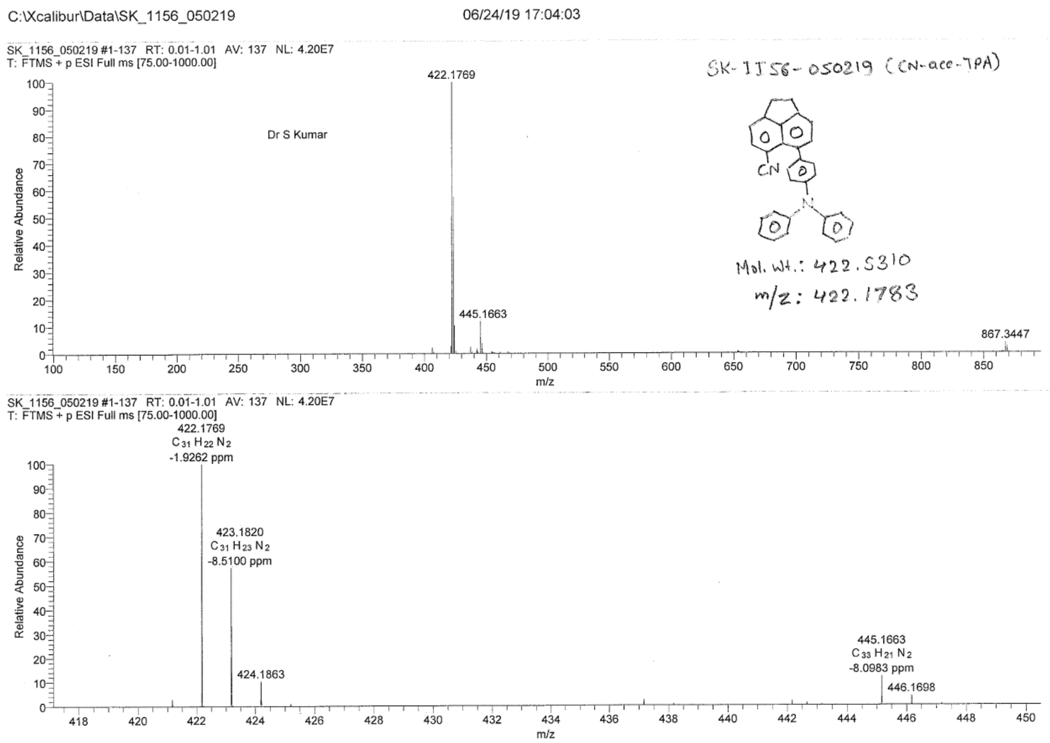

Figure **S24**. HRMS of **TPA-ace-CN**.

HPLC Trace Report24Nov2020

<Sample Information>

Sample Name	: TPA-ace-CN		
Sample ID	:		
Method Filename	: 80% Acetonitrile 20 Water 20 mins.lcm		
Batch Filename	: Shiv_TPA-ace_series-MeCN-80.lcb		
Vial #	: 2-48	Sample Type	: Unknown
Injection Volume	: 2 uL		
Date Acquired	: 18/11/2020 13:08:55	Acquired by	: System Administrator
Date Processed	: 18/11/2020 13:28:58	Processed by	: System Administrator

<Chromatogram>

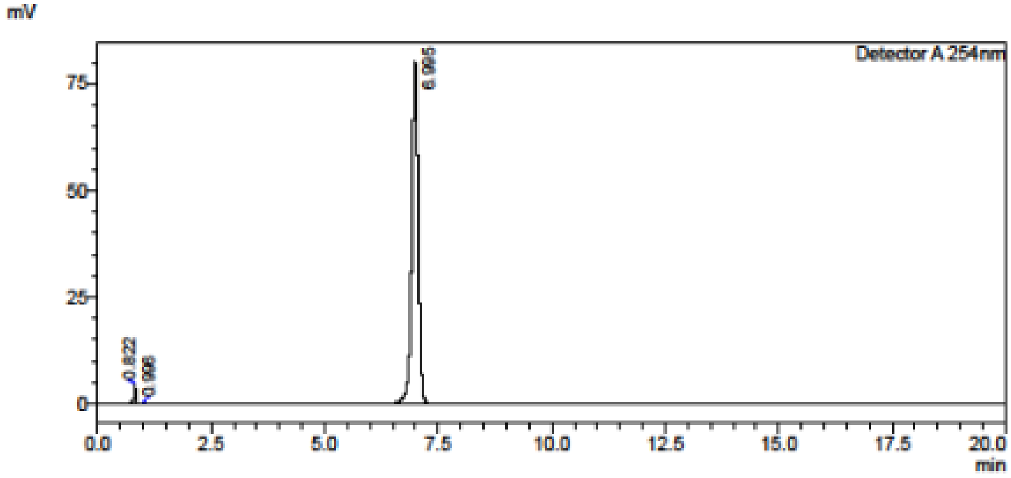

<Peak Table>

Detector A 254nm

Peak#	Ret. Time	Area	Height	Area%	Area/Height	Width at 5% Height
1	0.822	13726	4636	1.691	2.961	0.124
2	0.996	1529	521	0.188	2.936	--
3	6.995	796578	79994	98.121	9.958	0.348
Total		811833	85151	100.000		

Figure **S25.** HPLC analysis report for **TPA-ace-CN**.

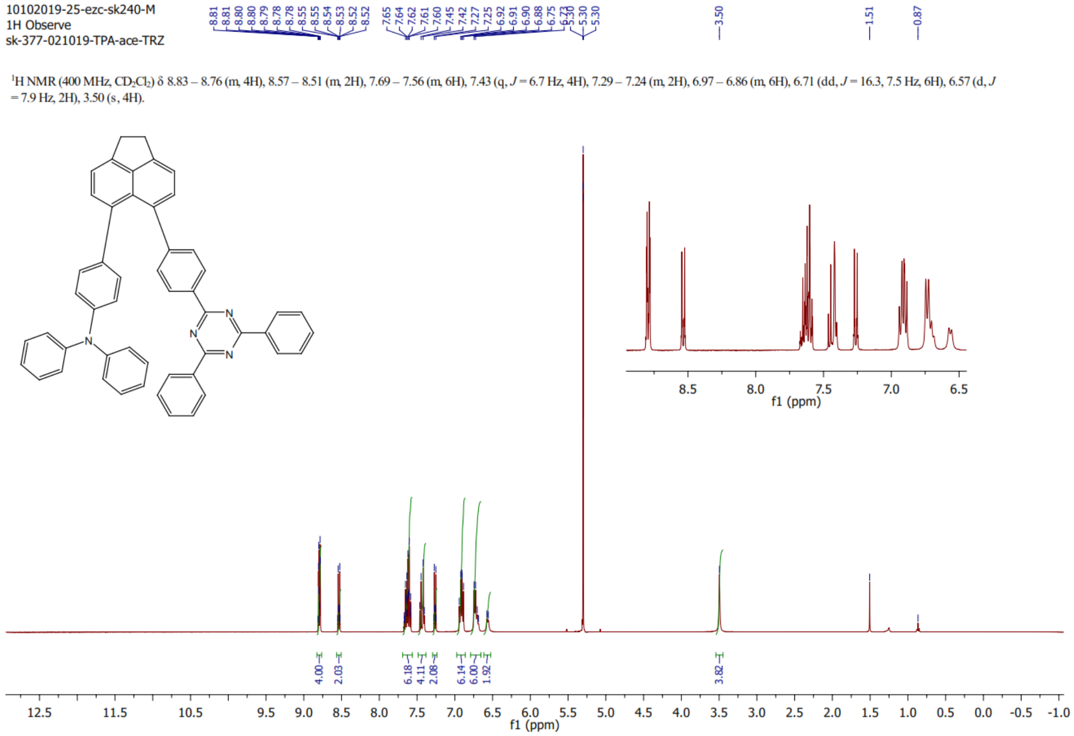

Figure **S26**. ^{1}H NMR spectra of **TPA-ace-TRZ.**

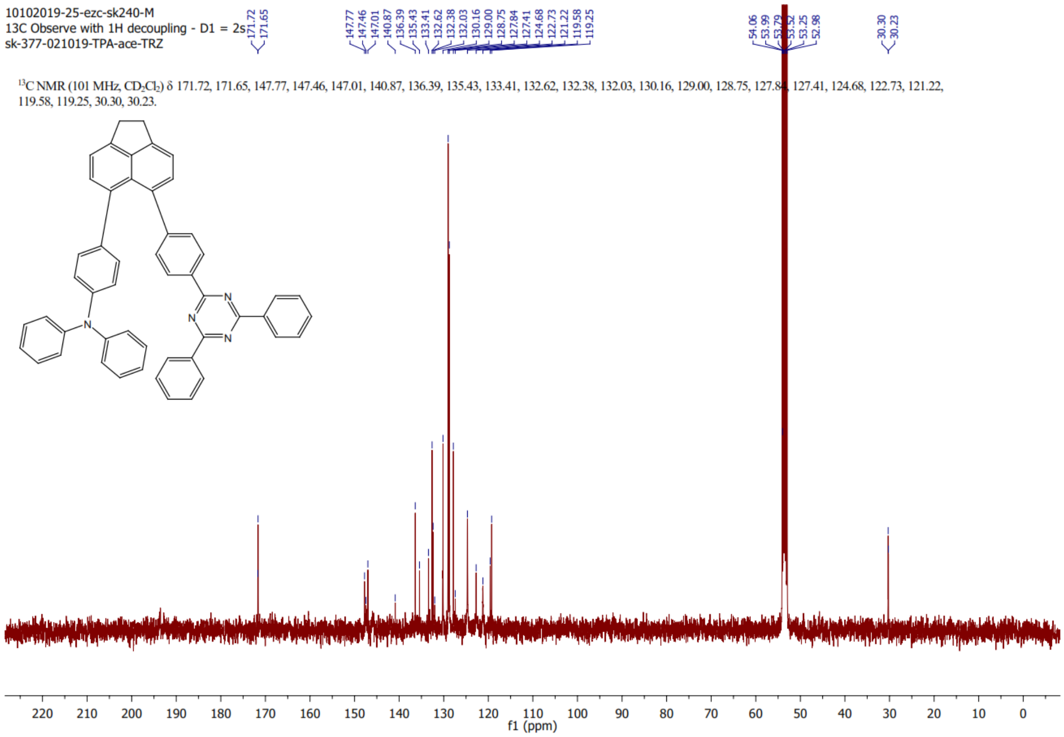

Figure **S27**.^{13}C NMR spectra of **TPA-ace-TRZ.**

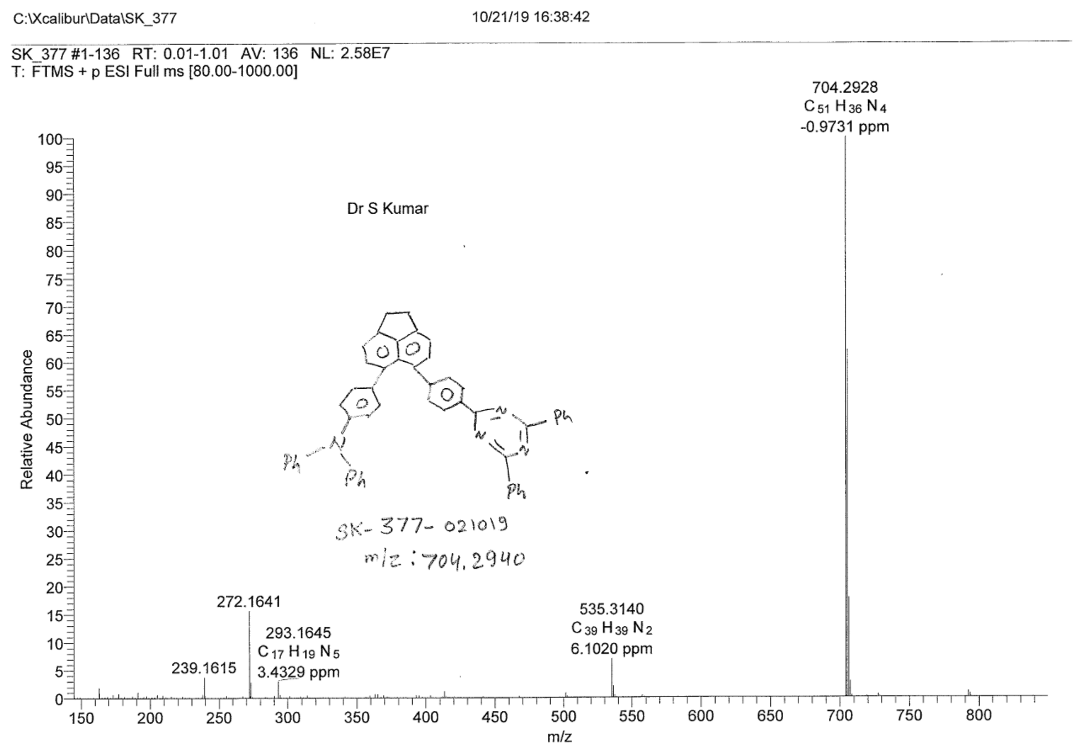

Figure **S28**. HRMS of **TPA-ace-TRZ**.

LONDON metropolitan university

Elemental Analysis Service

Please send completed form and samples to:

Stephen Boyer
School of Human Sciences
Science Centre
London Metropolitan University
29 Hornsey Road
London N7 7DD

Telephone: 020 7133 3605
Fax: 020 7133 2577
Email: s.boyer@londonmet.ac.uk

Sample submitted by: **Dr Shiv Kumar**	
Address: **EZC Group, North Haugh, School of Chemistry, University of St Andrews, KY16 9ST**	
Telephone: **01334 463848**	Email: **sk240@st-andrews.ac.uk**
Date Submitted: **04/10/2019**	

Please submit ca. 5 mg of sample.

Sample Reference No.: **SK-377-021019**
Name of Compound: **TPA-ace-TRZ**
Molecular Formula: **C51H36N4**
Stability: **Stable at room temperature and pressure**
Hazards: **Unknown**
Other Remarks:

Element	Expected %	Found (1)	Found (2)	
Carbon	**86.90**	86.71	86.81	
Hydrogen	**5.15**	5.02	5.07	
Nitrogen	**7.95**	8.06	8.13	

Authorising Signature:

Date Completed: 271019	Signature:
Comments:	

Figure **S29**. Elemental analysis report for **TPA-Ace-TRZ.**

HPLC Trace Report24Nov2020

<Sample Information>

Sample Name	: TPA-ace-TRZ		
Sample ID	:		
Method Filename	: 98% Acetonitrile 2 Water 20 mins.lcm		
Batch Filename	: Shiv_TPA-ace-TRZ.lcb		
Vial #	: 2-49	Sample Type	: Unknown
Injection Volume	: 2 uL		
Date Acquired	: 24/11/2020 09:26:45	Acquired by	: System Administrator
Date Processed	: 24/11/2020 09:51:47	Processed by	: System Administrator

<Chromatogram>

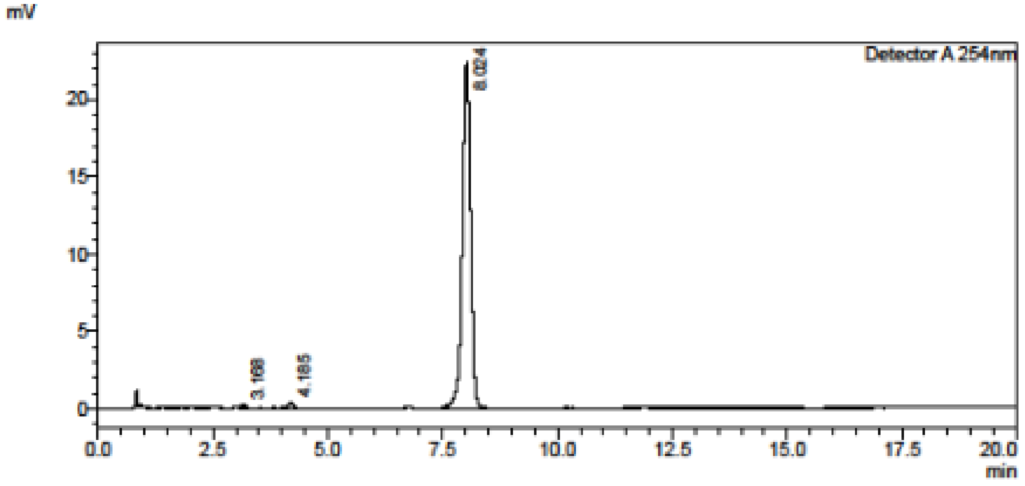

<Peak Table>

Detector A 254nm

Peak#	Ret. Time	Area	Height	Area%	Area/Height	Width at 5% Height
1	3.168	1369	222	0.491	6.161	--
2	4.185	2907	399	1.043	7.289	0.239
3	8.024	274351	22287	98.466	12.310	0.429
Total		278626	22908	100.000		

Figure **S30.** HPLC analysis report for **TPA-ace-TRZ**.

Single crystal X-ray diffraction analysis

X-ray diffraction data for all compounds were collected at 173 K using a Rigaku FR-X Ultrahigh Brilliance Microfocus RA generator/confocal optics with XtaLAB P200 diffractometer [Mo Kα radiation (λ = 0.71075 Å)]. Intensity data were collected using ω steps accumulating area detector images spanning at least a hemisphere of reciprocal space. Data for all compounds

analysed were collected using CrystalClear[8] and processed (including correction for Lorentz, polarization and absorption) using CrysAlisPro.[9] Structures were solved by direct methods (SIR2004[10]) and refined by full-matrix least-squares against F^2 (SHELXL-2018/3[11]). Non-hydrogen atoms were refined anisotropically, and hydrogen atoms were refined using a riding model. The structure of **2TPA-ace** affected by non-merohedral twinning, with a twin law [1 0 0 0.178 0 -1ß] and refined twin fraction of 0.21. All calculations were performed using the CrystalStructure[12] interface. Selected crystallographic data are presented in Table **S1**, and views of the structures and their packing are presented in Figure S31. Deposition numbers 2041945-2041948 contains the supplementary crystallographic data for this paper. These data are provided free of charge by the joint Cambridge Crystallographic Data Centre and Fachinformationszentrum Karlsruhe Access Structures service www.ccdc.cam.ac.uk/structures.

Table S1. Selected crystallographic data.

	**TPA-ace**	**TPA-ace-CN**	**2TPA-ace**	**TPA-ace-TRZ**
empirical formula	$C_{30.5}H_{24}ClN$	$C_{31}H_{22}N_2$	$C_{48}H_{36}N_2$	$C_{51}H_{36}N_4$
fw	439.98	422.53	640.83	704.87
crystal description	Yellow Prism	Orange chip	Orange prism	Orange prism
crystal size [mm^3]	0.13×0.06×0.01	0.09×0.09×0.07	0.24×0.08×0.06	0. 09×0.09×0.02
space group	$C2/c$	Cc	$P2/c$	$P\bar{1}$
a [Å]	25.1888(14)	9.0560(3)	13.9080(7)	10.8393(3)
b [Å]	7.4270(3)	16.5151(5)	9.4513(5)	12.3707(3)
c [Å]	25.1491(14)	15.0808(4)	26.3678(13)	13.7273(3)
α [°]				89.441(2)
β [°]	107.297(6)	102.313(3)	99.727(5)	80.964(2)
γ [°]				88.169(2)
vol [Å]^3	4492.1(4)	2203.61(12)	3416.2(3)	1816.90(8)
Z	8	4	4	2
ρ (calc) [g/cm^3]	1.301	1.273	1.246	1.288
μ [mm^{-1}]	0.189	0.074	0.072	0.076
F(000)	1848	888	1352	740
reflections collected	28249	14251	43282	24016
independent reflections (R_{int})	5210 (0.0546)	4793 (0.0294)	7975 (0.0584)	7964 (0.0380)
parameters, restraints	294, 0	298, 2	452, 0	496, 0
GOF on F^2	1.058	1.046	1.061	1.031
R_1 [$I > 2\sigma(I)$]	0.0665	0.0360	0.0603	0.0482
wR_2 (all data)	0.1945	0.0929	0.1732	0.1118
largest diff. peak/hole [e/Å^3]	0.40, -0.81	0.17, -0.18	0.24, -0.28	0.21, -0.21

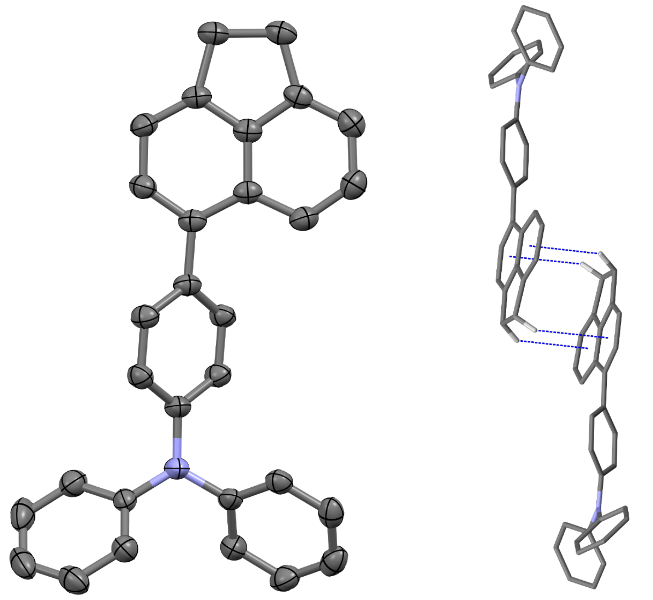

Figure S31 X-ray structure of **TPA-ace** (left) Thermal-ellipsoid plot (ellipsoids drawn at the 50% probability level, hydrogens omitted) and (right) view of the CH···π interactions, leading to formation of dimers (non-interacting hydrogens omitted).

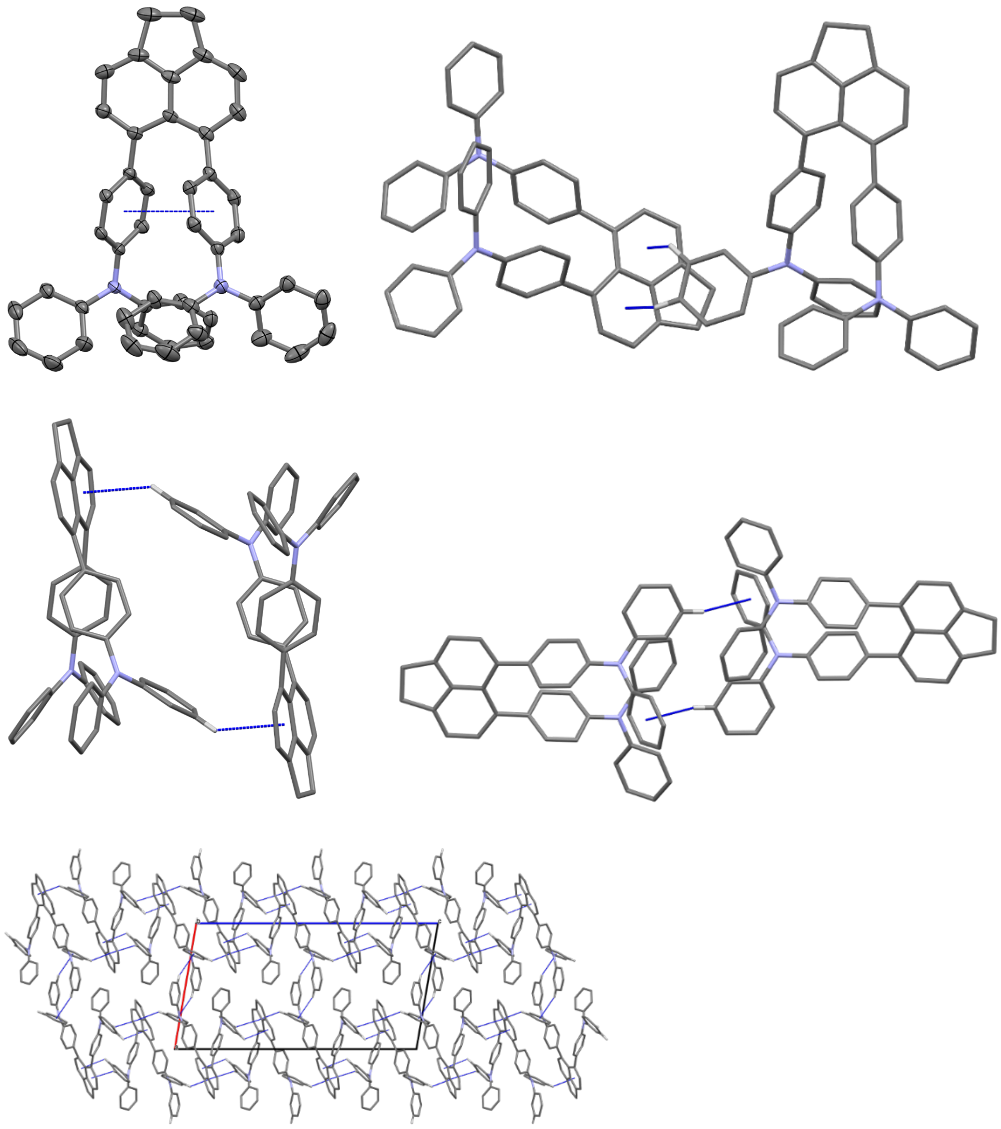

Figure S32. X-ray structure of **2TPA-ace** (a) Thermal-ellipsoid plot, showing the intramolecular π···π interaction (ellipsoids drawn at the 50% probability level, hydrogens omitted), (b-e) views of the intermolecular interactions and packing (non-interacting hydrogens omitted): (b-d) the four different sets of CH···π interactions, (e) view of the packing down the crystallographic *b*-axis.

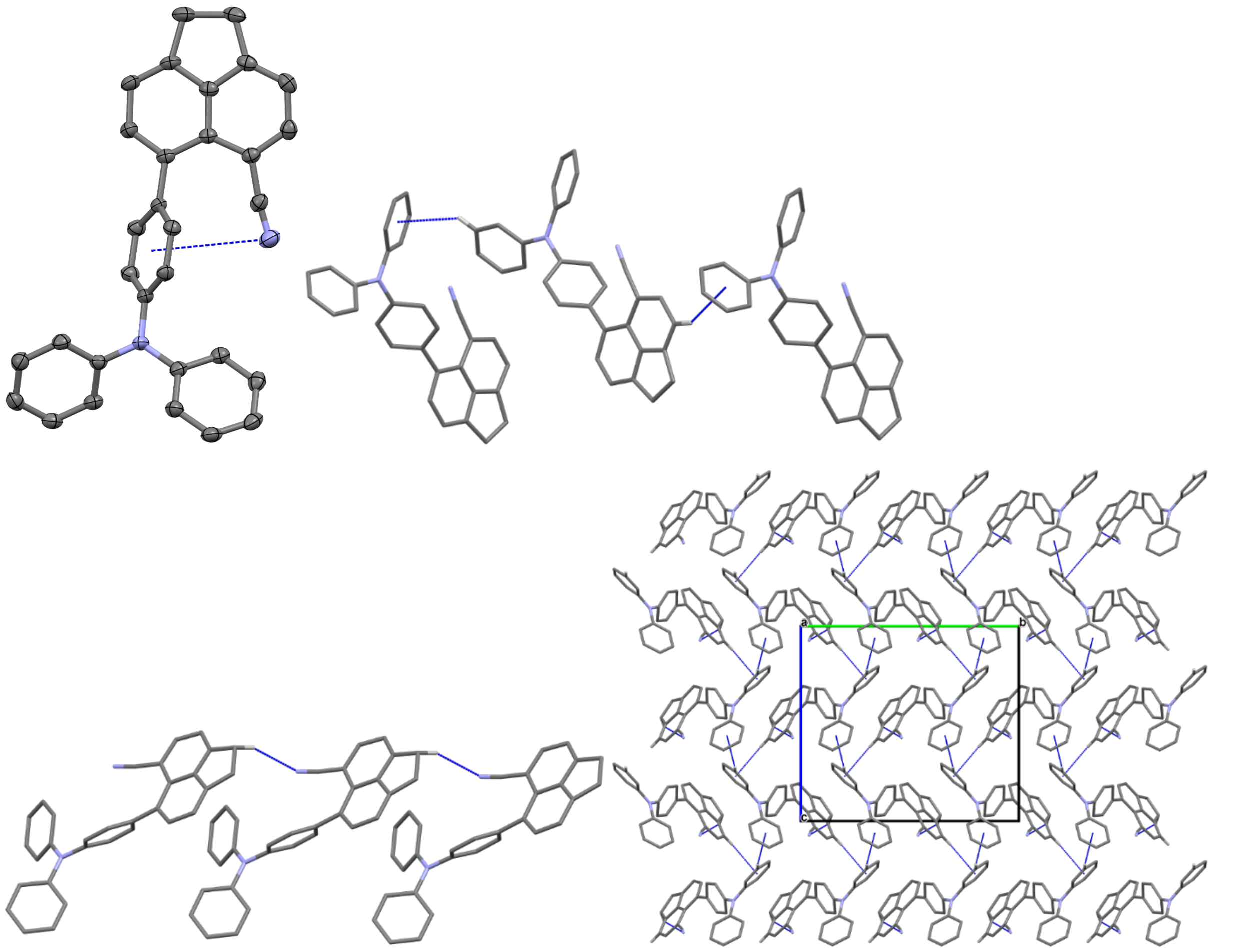

Figure S33. X-ray structure of **TPA-ace-CN** (a) Thermal-ellipsoid plot, showing the intramolecular CN···π interaction (ellipsoids drawn at the 50% probability level, hydrogens omitted), (b-d) views of the intermolecular interactions and packing (non-interacting hydrogens omitted): (b) CH···π interactions, (c) CH···N hydrogen bonds (e) view of the packing down the crystallographic *a*-axis.

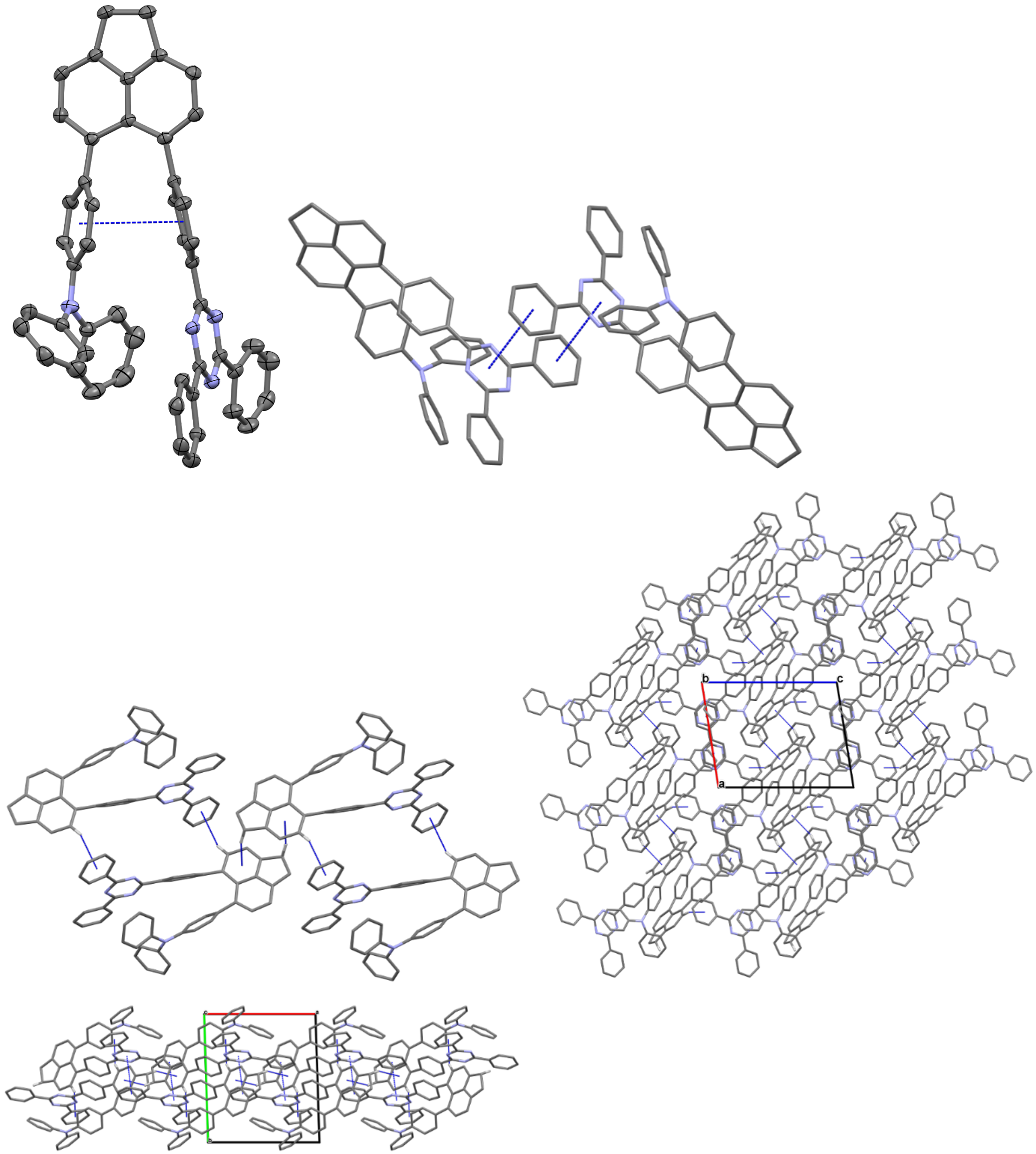

Figure S34. X-ray structure of **TPA-ace-TRZ** (a) Thermal-ellipsoid plot, showing the intramolecular π···π interaction (ellipsoids drawn at the 50% probability level, hydrogens omitted), (b-e) views of the intermolecular interactions and packing (non-interacting hydrogens omitted): (b) π···π interactions, (c) the two different sets of CH···π interactions, (d) view of the packing down the crystallographic *b*-axis (e) view of one two-dimensional sheet in the *ac*-plane.

Cartesian coordinates of the ground-state optimized geometries

TPA-ace

C	-4.30193200	2.58788900	0.38458400
C	-3.27562100	1.67155800	0.28418800
C	-3.55987000	0.29898600	0.06278900
C	-4.92647800	-0.04209000	-0.00893000
C	-5.97444900	0.89469900	0.09146900
C	-5.66612900	2.21910900	0.28187300
H	-4.05676100	3.63217900	0.55773300
H	-2.24724300	1.99974400	0.39125600
C	-2.62529300	-0.77863200	-0.05415800
C	-5.40606000	-1.35649700	-0.16683400
H	-6.43881300	2.97821600	0.36720500
C	-4.49487100	-2.38009400	-0.25021600
C	-3.11632200	-2.06898700	-0.19514800
H	-4.80548400	-3.41434100	-0.37020300
H	-2.39752900	-2.87782800	-0.29677600
C	-7.29425000	0.16971600	-0.02992600
H	-7.91746100	0.32970700	0.85660900
H	-7.87095300	0.53743500	-0.88572100
C	-6.91515100	-1.33076000	-0.19929600
H	-7.34010900	-1.94570900	0.60163200
H	-7.29903700	-1.73893600	-1.14077200
C	-1.16623900	-0.55251000	-0.02910600
C	-0.33550300	-1.33713500	0.78053900
C	-0.55915700	0.42068900	-0.83414300
C	1.03871800	-1.15349000	0.80023600
H	-0.78481400	-2.08203300	1.43126500
C	0.81569000	0.60123000	-0.83480800
H	-1.17430400	1.01824300	-1.50026200
C	1.63341900	-0.18059100	-0.01089400
H	1.66330300	-1.75798400	1.45003500
H	1.26920500	1.34534400	-1.48156500
N	3.02990600	0.00592200	0.00016700
C	3.88762700	-1.11203200	0.06222200
C	5.04594900	-1.07470000	0.84699900
C	3.58995400	-2.27292800	-0.66057400
C	5.89156000	-2.17566800	0.89577700
H	5.27549500	-0.17718100	1.41221200
C	4.43228600	-3.37509500	-0.58936200
H	2.69485400	-2.30135800	-1.27345700
C	5.58994300	-3.33377200	0.18357100
H	6.78699200	-2.13114000	1.50895300
H	4.18743200	-4.26870600	-1.15615900
H	6.24962500	-4.19444800	0.23072900
C	3.56862300	1.30736800	-0.06643800
C	4.71158600	1.56396100	-0.83243900
C	2.96552100	2.35947000	0.63254300
C	5.24197900	2.84668200	-0.88708300

H	5.17780100	0.75073600	-1.37918100
C	3.49291300	3.64214200	0.55514800
H	2.08272500	2.16123900	1.23177500
C	4.63561300	3.89496000	-0.19965200
H	6.12956800	3.02931800	-1.48590400
H	3.01302600	4.44786700	1.10316000
H	5.04881700	4.89731600	-0.25133100

2TPA-ace

C	-4.05834900	-2.48999400	-1.42778000
C	-3.40279700	-1.53243100	-0.66625600
C	-4.18856200	-0.56166900	0.03596800
C	-5.58307600	-0.77315100	0.03651400
C	-6.23008500	-1.77081500	-0.71910000
C	-5.46562100	-2.60699500	-1.49462300
H	-3.44949400	-3.22961400	-1.94109700
C	-3.73212900	0.60066700	0.73595500
C	-6.49743600	-0.00853200	0.78868100
H	-5.91105600	-3.38583700	-2.10732700
C	-6.01862400	1.02232300	1.55803500
C	-4.63894300	1.32760600	1.49161500
H	-6.67610900	1.63659600	2.16685500
H	-4.27479400	2.21397000	2.00455300
C	-7.71964900	-1.67280800	-0.49409100
H	-8.23859900	-1.42294200	-1.42647200
H	-8.14042300	-2.62335100	-0.14944900
C	-7.89051900	-0.54564400	0.56496600
H	-8.57564500	0.23663800	0.22166400
H	-8.30952500	-0.93861700	1.49824400
C	-2.38194000	1.17210500	0.50934100
C	-1.46449200	1.39053600	1.53840300
C	-2.04961200	1.58536100	-0.78669000
C	-0.23683400	1.99107100	1.27723800
H	-1.70927400	1.07085800	2.54712200
C	-0.84043700	2.21032000	-1.04637300
H	-2.76133100	1.42032400	-1.59008000
C	0.07937100	2.40652800	-0.01547800
H	0.48330400	2.15440500	2.07350200
H	-0.59306300	2.55546000	-2.04579200
N	1.33054300	3.03216400	-0.27937400
C	2.45664400	2.20120400	-0.46679100
C	3.68933500	2.54819700	0.09930900
C	2.34884600	1.01194200	-1.19484800
C	4.79562200	1.72919000	-0.08207800
H	3.76574800	3.46442900	0.67648800
C	3.45995700	0.19316500	-1.36214600
H	1.38936500	0.72331000	-1.61153600
C	4.68828400	0.54920400	-0.81510500
H	5.74504200	2.00773800	0.36567800
H	3.36356700	-0.73216100	-1.92184000

H	5.55529600	-0.08937500	-0.95385600
C	1.35695700	4.40291300	-0.56947700
C	2.36652600	4.96571800	-1.36408200
C	0.34640600	5.24015900	-0.07361200
C	2.36814700	6.32832800	-1.63277400
H	3.14384500	4.33057500	-1.77433700
C	0.35534400	6.59851800	-0.36159300
H	-0.44377300	4.81736000	0.53764100
C	1.36639800	7.15844800	-1.13738000
H	3.16002800	6.74106500	-2.25167200
H	-0.43786400	7.22628300	0.03475000
H	1.37179800	8.22163300	-1.35504400
C	-1.94697900	-1.67657300	-0.44549500
C	-1.46998700	-1.79956000	0.86525500
C	-1.02614000	-1.81138500	-1.48798800
C	-0.13853600	-2.06723000	1.12900600
H	-2.17173000	-1.71841800	1.69019200
C	0.31836600	-2.05259700	-1.23420700
H	-1.36515100	-1.70341300	-2.51477100
C	0.77919100	-2.19841500	0.08056700
H	0.20185500	-2.18589100	2.15212700
H	1.01925200	-2.14377600	-2.05793900
N	2.13145700	-2.47988800	0.35438200
C	2.73867900	-1.92954300	1.50778800
C	3.57021600	-2.71243500	2.31458700
C	2.51145400	-0.59367400	1.85173400
C	4.18202000	-2.15608100	3.43192700
H	3.73846800	-3.75275100	2.05512700
C	3.11656600	-0.05087300	2.97662300
H	1.87147000	0.01440700	1.22356200
C	3.95969700	-0.82419900	3.77121400
H	4.82831300	-2.77490700	4.04772600
H	2.94397100	0.99424000	3.21743500
H	4.43931200	-0.39364800	4.64478900
C	2.85933700	-3.36308000	-0.46645500
C	4.22541900	-3.15076800	-0.69521200
C	2.23787600	-4.46875600	-1.06009200
C	4.94498600	-4.02237900	-1.50216800
H	4.71203700	-2.30093700	-0.22905200
C	2.96398500	-5.32441700	-1.87903100
H	1.18407200	-4.64925300	-0.87580900
C	4.32100000	-5.11098300	-2.10625500
H	6.00296400	-3.84003200	-1.66801600
H	2.46362900	-6.17595500	-2.33120300
H	4.88518800	-5.78545000	-2.74248200

TPA-ace-Br

C	2.93006500	2.38671900	0.88404400
C	2.39371500	1.20034200	0.41317500
C	3.27647500	0.15701500	-0.01372400

C	4.66150900	0.41841800	0.14213700
C	5.18352600	1.63195300	0.63741300
C	4.31679100	2.63054200	0.99757300
H	2.23404300	3.15902900	1.19934700
C	2.98621500	-1.11561100	-0.58955600
C	5.69151600	-0.48721000	-0.18292300
H	4.66821300	3.58565400	1.37747300
C	5.35746700	-1.71238000	-0.70023900
C	3.99249000	-2.00593000	-0.90684500
H	6.10829900	-2.45051800	-0.96648100
H	3.71509600	-2.95969200	-1.34256100
C	7.02820500	0.13559200	0.13304500
H	7.67327600	0.16653400	-0.75148900
H	7.56386400	-0.44966400	0.88862200
C	6.69071000	1.56092800	0.65086200
H	7.12472600	2.33456900	0.00800700
H	7.08864700	1.73273000	1.65667300
C	0.91811300	1.04082900	0.41983400
C	0.29731900	0.32822000	1.44919200
C	0.12673000	1.56466800	-0.60282600
C	-1.06929600	0.10351100	1.43372200
H	0.90132300	-0.06928600	2.25947800
C	-1.23872100	1.32095800	-0.64207300
H	0.59802300	2.11964700	-1.40855000
C	-1.84598600	0.56508000	0.36541700
H	-1.54427400	-0.46210400	2.22880000
H	-1.83974200	1.69247100	-1.46566800
Br	1.22752200	-1.67656600	-1.03471200
N	-3.21805900	0.24402900	0.30073300
C	-3.63230500	-1.07877700	0.56020500
C	-4.87622100	-1.32414200	1.15436900
C	-2.80767800	-2.16312800	0.23435500
C	-5.28892700	-2.62712500	1.40103800
H	-5.51346600	-0.48599700	1.41705300
C	-3.22383800	-3.46074400	0.50335800
H	-1.83404800	-1.98181400	-0.20941200
C	-4.46699700	-3.70471400	1.08088200
H	-6.25739500	-2.79856600	1.86211000
H	-2.57011200	-4.28937900	0.24686000
H	-4.78960000	-4.72123600	1.28271000
C	-4.13701500	1.20366700	-0.17108200
C	-4.03366500	2.53459300	0.24988700
C	-5.15126600	0.84918300	-1.06784900
C	-4.92335800	3.48937300	-0.22529600
H	-3.24888400	2.80929200	0.94747800
C	-6.04792400	1.80837500	-1.52202800
H	-5.22922300	-0.18005300	-1.40242000
C	-5.93922600	3.13375500	-1.10917300
H	-4.82898000	4.51800900	0.11057100
H	-6.82939800	1.51719600	-2.21796700
H	-6.63778300	3.88103900	-1.47217800

TPA-ace-CN

C	-4.05021200	2.24365000	0.97652800
C	-3.05782900	1.32559900	0.64519000
C	-3.41277900	0.05019600	0.09576800
C	-4.79925300	-0.18474000	-0.02190600
C	-5.79668800	0.75175400	0.31462700
C	-5.42324500	1.97935300	0.80721200
H	-3.74176400	3.19680600	1.39428000
C	-2.55025300	-1.00891700	-0.31440400
C	-5.35616300	-1.39719100	-0.47559600
H	-6.15292800	2.73487400	1.08182800
C	-4.50986900	-2.42024700	-0.82229700
C	-3.11611300	-2.20091900	-0.73925400
H	-4.88355500	-3.37934100	-1.16938700
H	-2.44097500	-2.99601000	-1.04324900
C	-7.15305900	0.15059000	0.04587600
H	-7.77058100	0.14830900	0.95036000
H	-7.69830300	0.73818500	-0.70088000
C	-6.86122900	-1.29021300	-0.45966800
H	-7.30282600	-2.04384900	0.20127500
H	-7.28382500	-1.46293900	-1.45516800
C	-1.71406100	1.71720900	0.94208800
N	-0.66713300	2.12359400	1.24225500
C	-1.07842500	-0.84443600	-0.31653800
C	-0.46358300	-0.01533700	-1.26001900
C	-0.27720100	-1.48753400	0.62950100
C	0.90446000	0.19518400	-1.23780300
H	-1.07162900	0.47495900	-2.01507900
C	1.09074900	-1.26392900	0.67426300
H	-0.74189700	-2.13183200	1.37037000
C	1.69371100	-0.40352300	-0.24942200
H	1.37274800	0.84925000	-1.96568200
H	1.69897900	-1.73380700	1.43999000
N	3.07022100	-0.11894100	-0.17876500
C	3.50884200	1.21441600	-0.34724800
C	4.70185700	1.48058600	-1.02794900
C	2.75168300	2.27723100	0.15935800
C	5.13661200	2.79062700	-1.18622200
H	5.28260800	0.65465900	-1.42628100
C	3.18831000	3.58360100	-0.02408000
H	1.82003500	2.08302200	0.68341000
C	4.38205100	3.85061800	-0.68990600
H	6.06580000	2.98263500	-1.71514700
H	2.58918100	4.39817900	0.37227700
H	4.72056000	4.87366600	-0.82200900
C	3.98629700	-1.13402000	0.15867300
C	5.06244600	-0.87224300	1.01500800
C	3.82971600	-2.42416800	-0.36263100
C	5.96358600	-1.88083100	1.33186700

H	5.18438900	0.12491000	1.42494700
C	4.72582100	-3.42966800	-0.02397300
H	3.00012200	-2.62766800	-1.03209100
C	5.80136700	-3.16585600	0.82045300
H	6.79303500	-1.66092200	1.99787800
H	4.58918700	-4.42478500	-0.43755500
H	6.50474100	-3.95192300	1.07650700

TPA-ace-TRZ

C	-4.51840400	-3.18805900	-1.39426200
C	-4.04112800	-2.04976100	-0.75804500
C	-4.97819300	-1.11666800	-0.19912200
C	-6.33636800	-1.51794300	-0.25594200
C	-6.79581000	-2.69354900	-0.88573100
C	-5.88475800	-3.52470300	-1.48449500
H	-3.78417200	-3.87641200	-1.80389000
C	-4.73619200	0.16653400	0.40132100
C	-7.40721700	-0.79281400	0.30599200
H	-6.18454300	-4.43785600	-1.99064000
C	-7.14017200	0.39242200	0.94126700
C	-5.80839800	0.85430700	0.95767900
H	-7.92513200	0.99262800	1.39252300
H	-5.60670100	1.83146200	1.38752700
C	-8.29663000	-2.77952700	-0.76393800
H	-8.76963300	-2.77155400	-1.75228300
H	-8.60607400	-3.71158300	-0.27912400
C	-8.69994100	-1.53260200	0.06889500
H	-9.42834500	-0.90763600	-0.45871300
H	-9.16585600	-1.81849400	1.01857000
C	-3.43125400	0.86669300	0.39929800
C	-2.96469200	1.49017000	1.56317500
C	-2.67749300	1.00590000	-0.77199800
C	-1.79393200	2.23637800	1.55699800
H	-3.52776100	1.37817400	2.48546800
C	-1.48966500	1.72201000	-0.77644000
H	-3.03881500	0.56035400	-1.69224500
C	-1.04614400	2.35528800	0.38501800
H	-1.44972400	2.73846000	2.45627300
H	-0.90725600	1.82621800	-1.68635300
N	0.13275200	3.14757300	0.35362700
C	1.38929600	2.49520600	0.35244000
C	1.67593000	1.51479500	1.30412900
C	2.36146300	2.84709000	-0.58906100
C	2.93339900	0.92215100	1.33599700
H	0.91278400	1.23484800	2.02352500
C	3.61304800	2.24487800	-0.55751900
H	2.12356100	3.60247200	-1.33170400
C	3.90813700	1.28837700	0.41197300
H	3.15352100	0.17718200	2.09437300
H	4.35902800	2.51269300	-1.29983100

H	4.88476500	0.81579900	0.43446100
C	0.03499400	4.53217000	0.53505200
C	1.11799100	5.28276100	1.01705600
C	-1.16476800	5.19744900	0.23818500
C	0.99955800	6.65679100	1.18177600
H	2.04810500	4.78391900	1.26562700
C	-1.27011900	6.56983100	0.42041700
H	-2.00981600	4.63069500	-0.13773000
C	-0.19150200	7.31506800	0.88890200
H	1.85183500	7.21609700	1.55781500
H	-2.20916000	7.06186000	0.18233100
H	-0.27798600	8.38836500	1.02451400
C	-2.56912700	-1.93170600	-0.64387800
C	-1.94387000	-1.85058000	0.60586100
C	-1.77239600	-1.96523500	-1.79423200
C	-0.56247700	-1.81512800	0.70233200
H	-2.55460100	-1.82198900	1.50281300
C	-0.39185900	-1.88705300	-1.70460900
H	-2.25337500	-2.02450600	-2.76664900
C	0.22536500	-1.82625000	-0.45293900
H	-0.07181300	-1.77675400	1.66891700
H	0.22902100	-1.88670500	-2.59426100
C	1.69354300	-1.81242500	-0.36023800
C	3.70781600	-1.41936700	-1.30173900
C	3.58423400	-2.24725700	0.79704900
N	2.24866100	-2.22104700	0.78489000
N	2.37959100	-1.40321000	-1.43111600
N	4.35805000	-1.86851400	-0.22307200
C	4.24865000	-2.69638400	2.03645700
C	5.64372400	-2.68269000	2.13395500
C	3.48395500	-3.12357200	3.12700600
C	6.26411600	-3.09134000	3.30702600
H	6.22115400	-2.34681500	1.27968200
C	4.10842200	-3.53110100	4.29846100
H	2.40360500	-3.13043400	3.03191200
C	5.49849200	-3.51551300	4.39114600
H	7.34756300	-3.07816300	3.37841500
H	3.51072600	-3.86311300	5.14205300
H	5.98538300	-3.83408700	5.30832000
C	4.51190900	-0.87415100	-2.41160000
C	5.90652300	-0.96860500	-2.38712100
C	3.88083600	-0.22294300	-3.47614100
C	6.65973800	-0.42227800	-3.41811600
H	6.37943600	-1.47311200	-1.55169100
C	4.63773400	0.32624100	-4.50259600
H	2.79887400	-0.14978400	-3.47010000
C	6.02747500	0.22693900	-4.47647400
H	7.74259900	-0.50080200	-3.39716100
H	4.14439500	0.83630500	-5.32458100
H	6.61801100	0.65717800	-5.28021700

Electrochemical properties

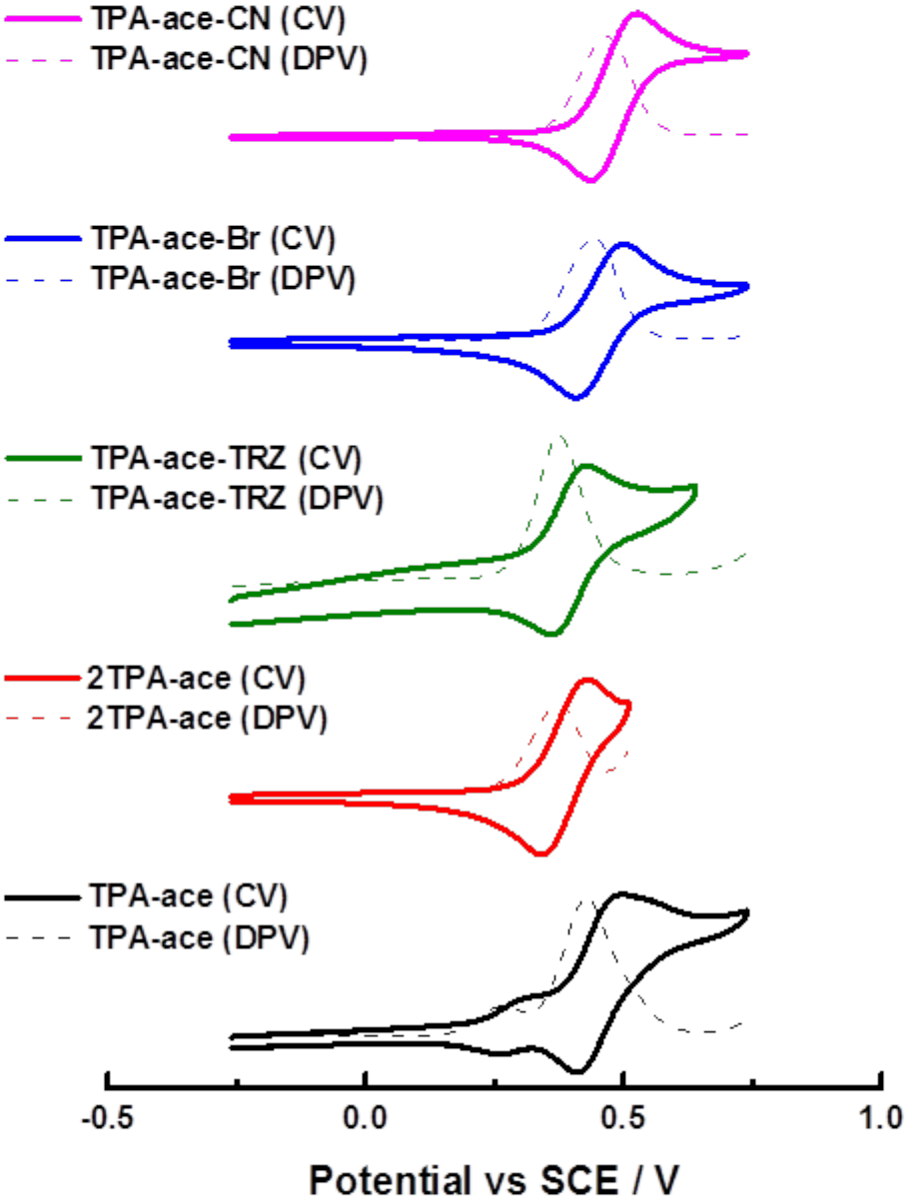

Figure **S35**. Anodic half wave cyclic voltammograms and DPV scans of the emitters (scans were carried out in degassed DCM at a scan rate of 100 mV s^{-1}).

Photophysical properties

Table **S2**. PLQY of the TPA-ace series in zeonex (1 wt% doping concentration, drop casted).

Emitter	PLQY Air; N_2 (%)[a]
**TPA-ace**	20.1; 20.8
**TPA-ace-Br**	1.8; 2.8
**TPA-ace-CN**	18.3; 18.8
**TPA-ace-TRZ**	12.2; 12.2
**2TPA-ace**	16.6; 17.0

a: λ_{exc}: 340 nm

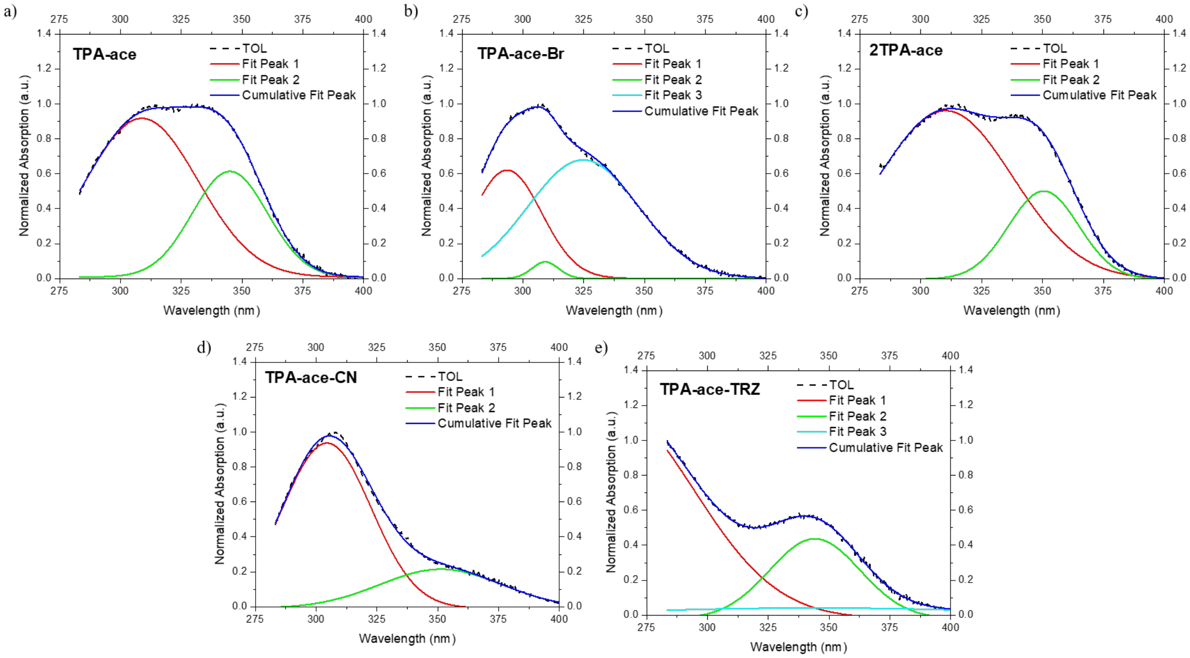

Figure **S36**. Deconvoluted absorption spectra of a) **TPA-ace**, b) **TPA-ace-Br**, c) **2TPA-ace**, d) **TPA-ace-CN** and e) **TPA-ace-TRZ** in toluene solution.

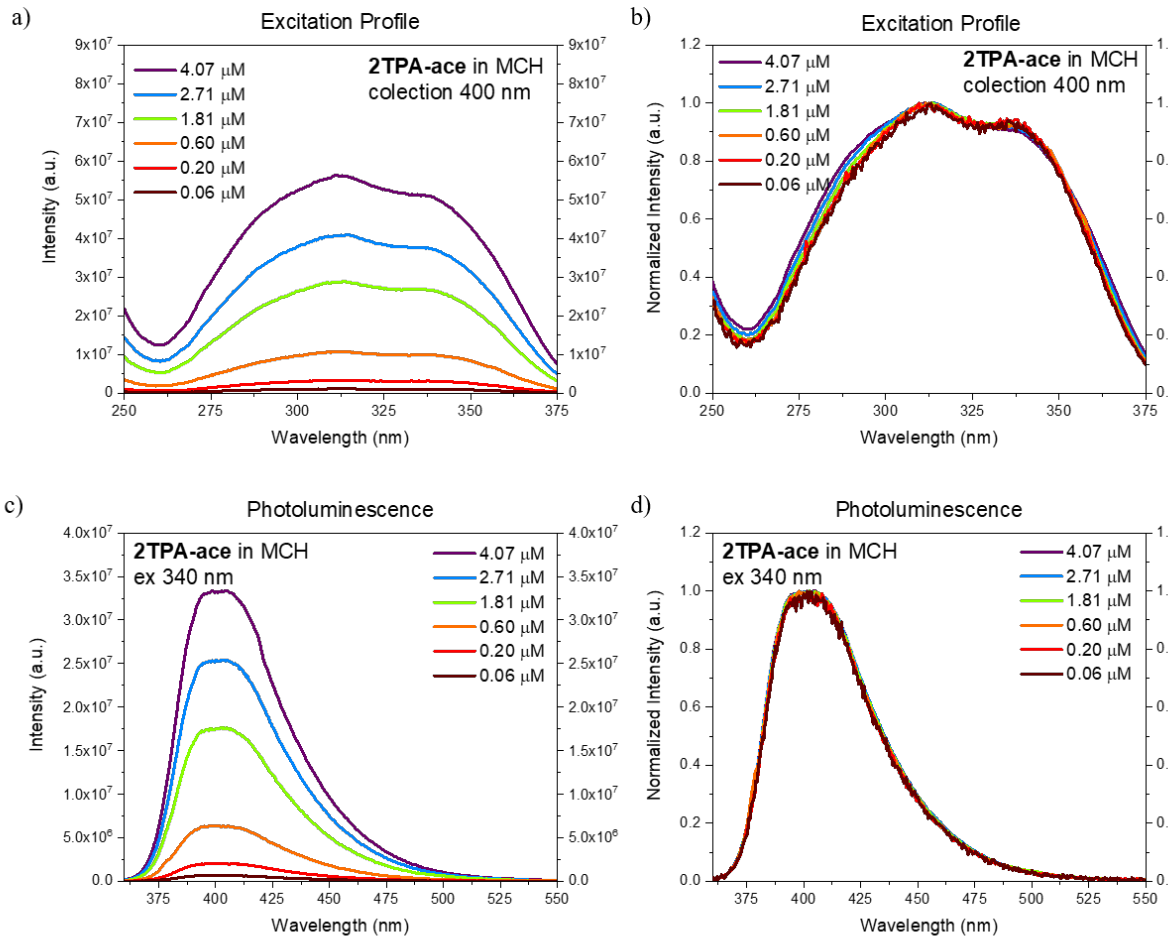

Figure **S37.** Concentration dependence of **2TPA-ace** in MCH solution

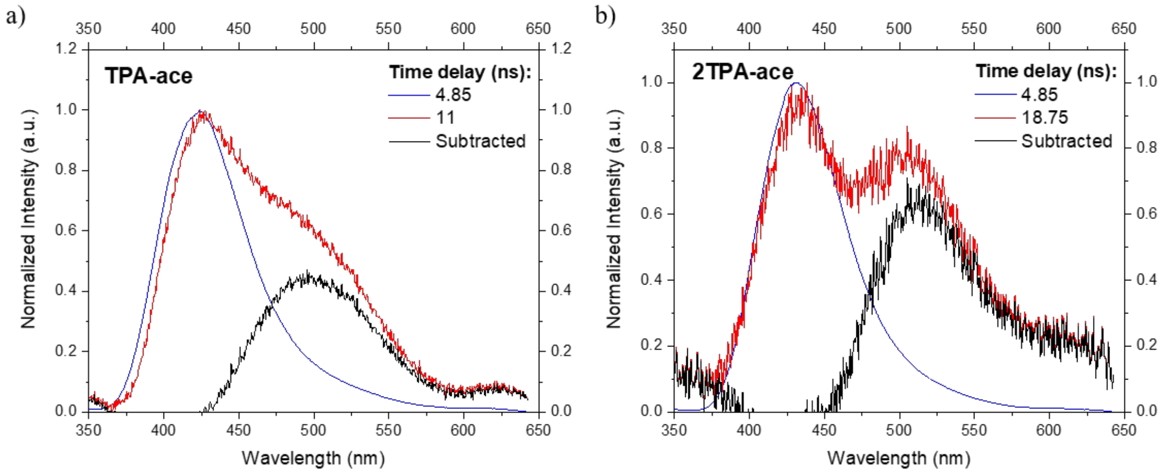

Figure **S38**. Peak of time-resolved spectra of a) **TPA-ace** and b) **2TPA-ace** in toluene solution, subtracting their respectively pure LE spectra.

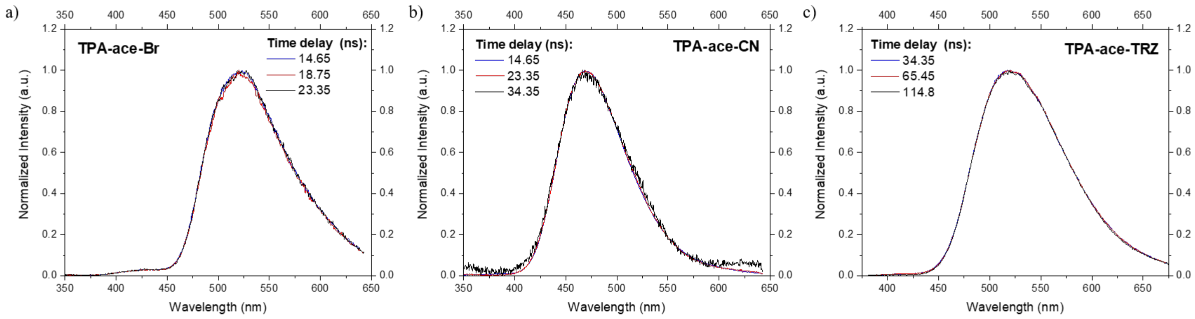

Figure **S39.** Peak of time-resolved spectra after stabilization of CT state for a) **TPA-ace-Br**, b) **TPA-ace-CN** and c) **TPA-ace-TRZ** in toluene solution.

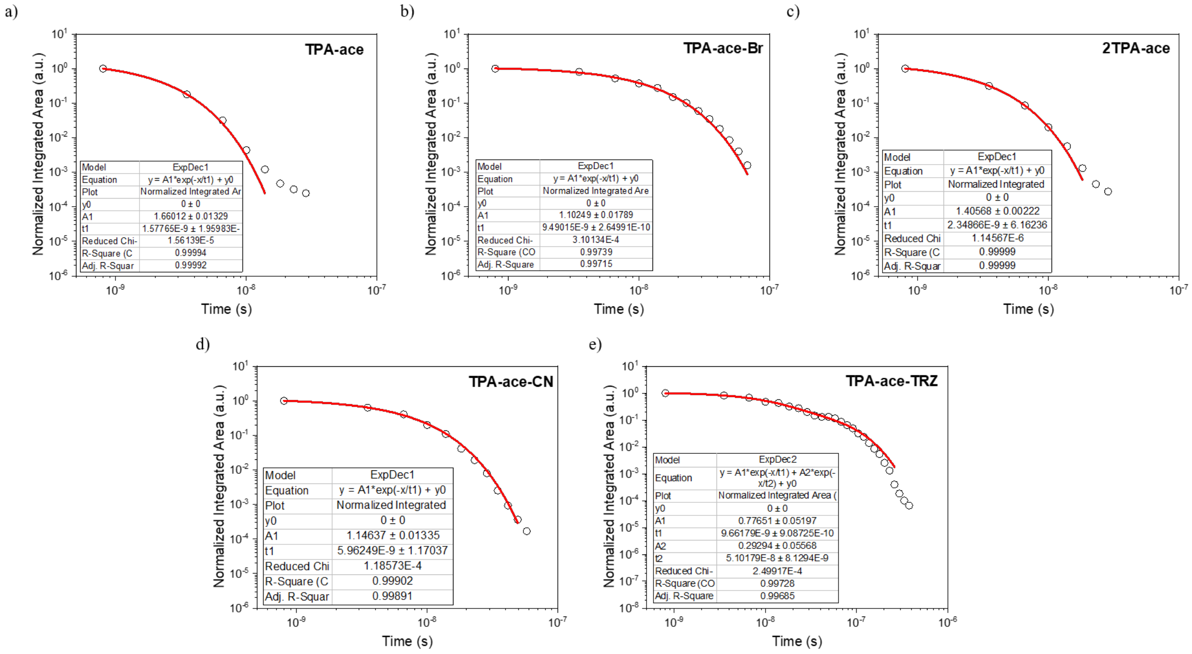

Figure **S40**. Fitting of kinetic decay results for a) **TPA-ace**, b) **TPA-ace-Br**, c) **2TPA-ace**, d) **TPA-ace-CN** and e) **TPA-ace-TRZ** in toluene solution.

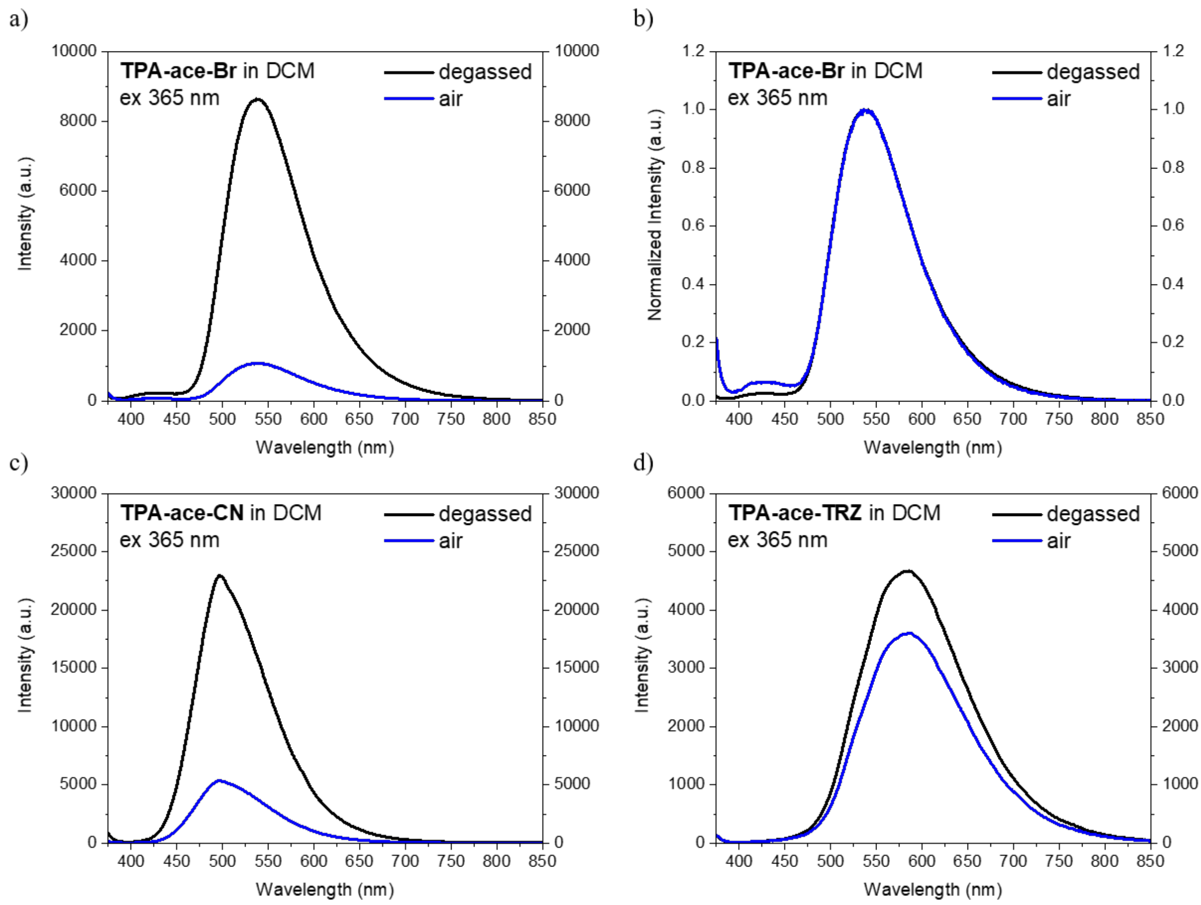

Figure **S41**. Oxygen quenching of total luminescence signal from for a) **TPA-ace-Br**, b) **TPA-ace-**Br (normalised), c) **TPA-ace-CN** and d) **TPA-ace-TRZ** in DCM solution.

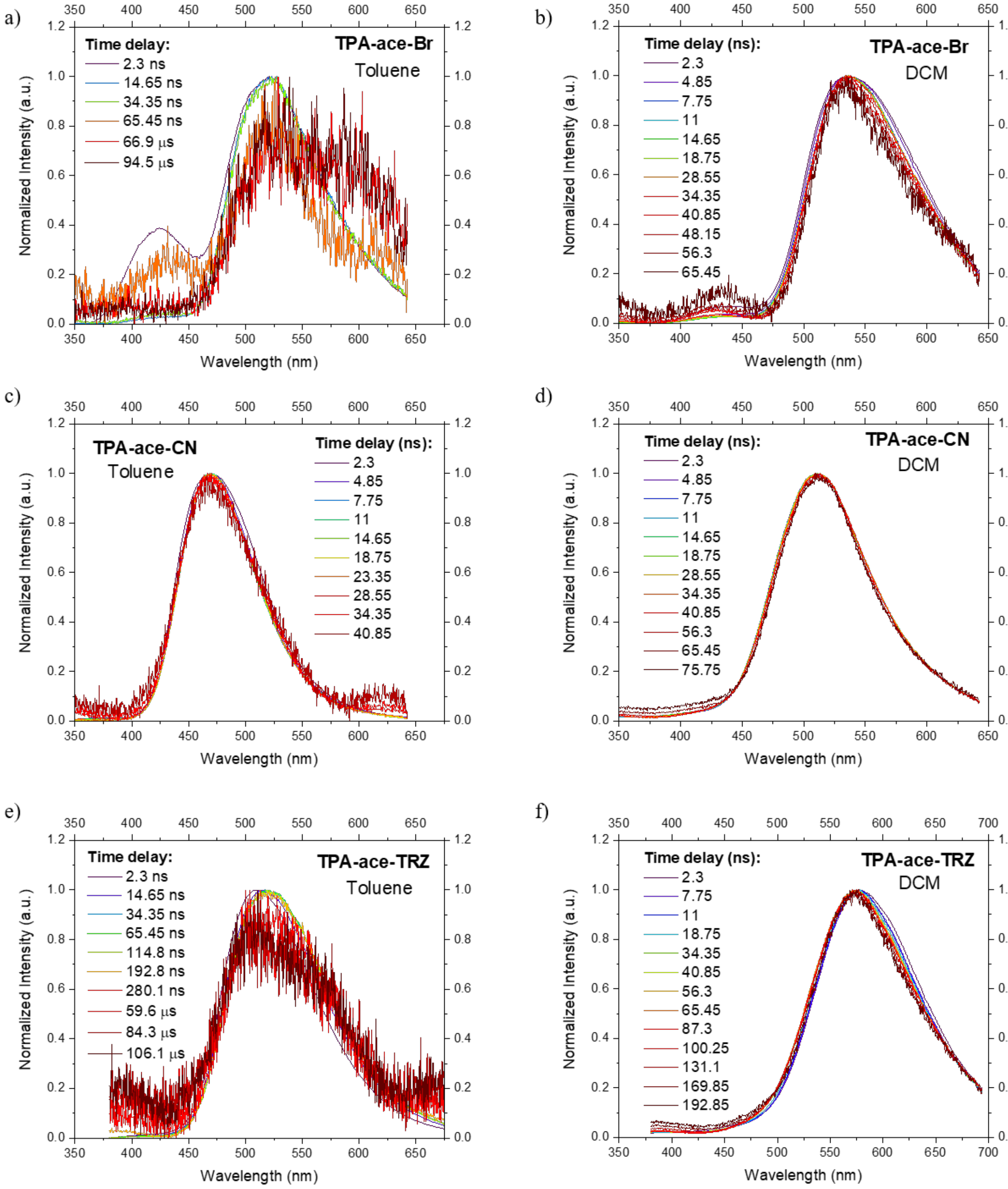

Figure **S42**. Comparison of the time resolved luminescence spectra decay of a) **TPA-ace-Br** in toluene **b) TPA-ace-Br** in DCM **c) TPA-ace-CN** in toluene d) **TPA-ace-CN** in DCM e) **TPA-ace-TRZ** in toluene and f) **TPA-ace-TRZ** in DCM solution.

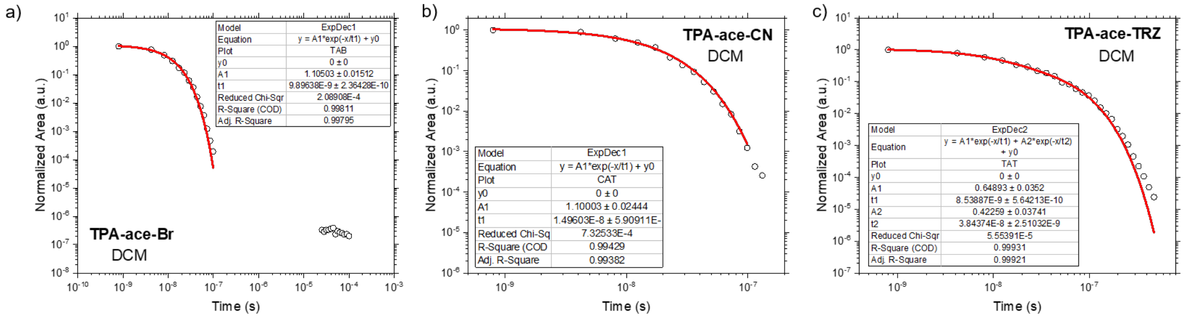

Figure **S43**. Fitted decay times from a) **TPA-ace-Br**, b) **TPA-ace-CN** and c) **TPA-ace-TRZ** dissolved in DCM, 20 μM excited at 355 nm.

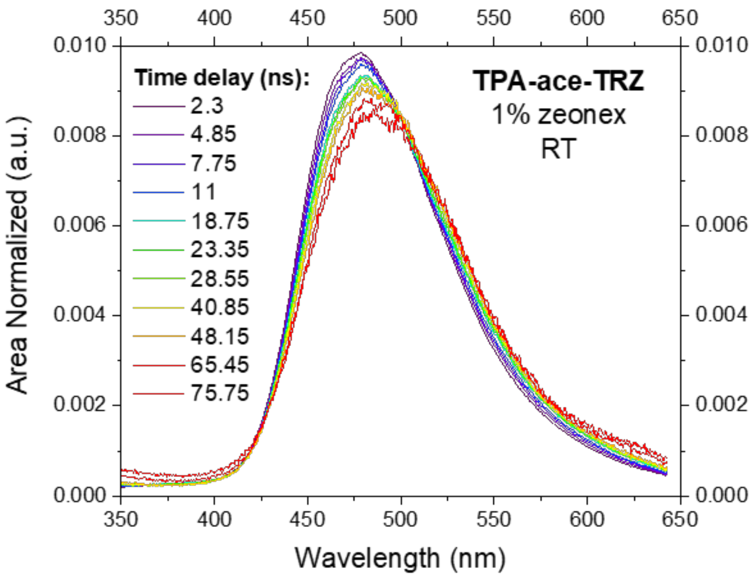

Figure **S44**. Area normalized emission spectra of **TPA-ace-TRZ** in 1% ZEONEX films at room temperature.

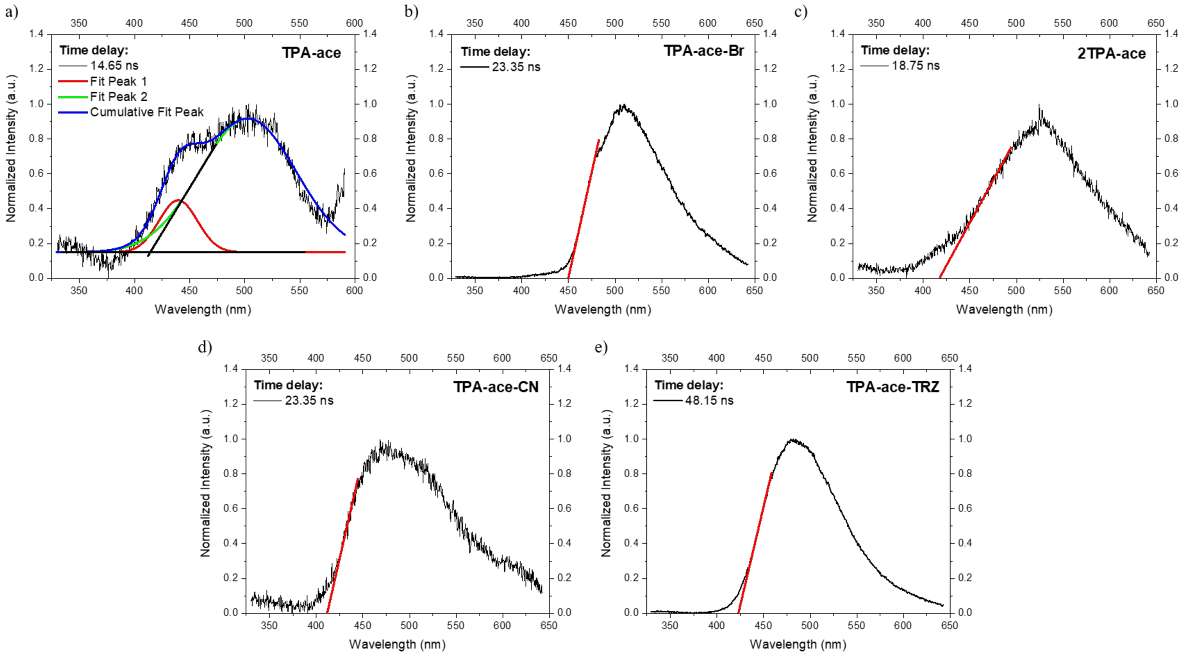

Figure **S45**. Onset of time resolved emission spectra for 1 wt% ZEONEX film of a) **TPA-ace**, which band was deconvoluted in order to obtain only ^{1}CT onset, b) **TPA-ace-Br**, c) **2TPA-ace**, d) **TPA-ace-CN,** e) **TPA-ace-TRZ**, obtained after stabilization of ^{1}CT.

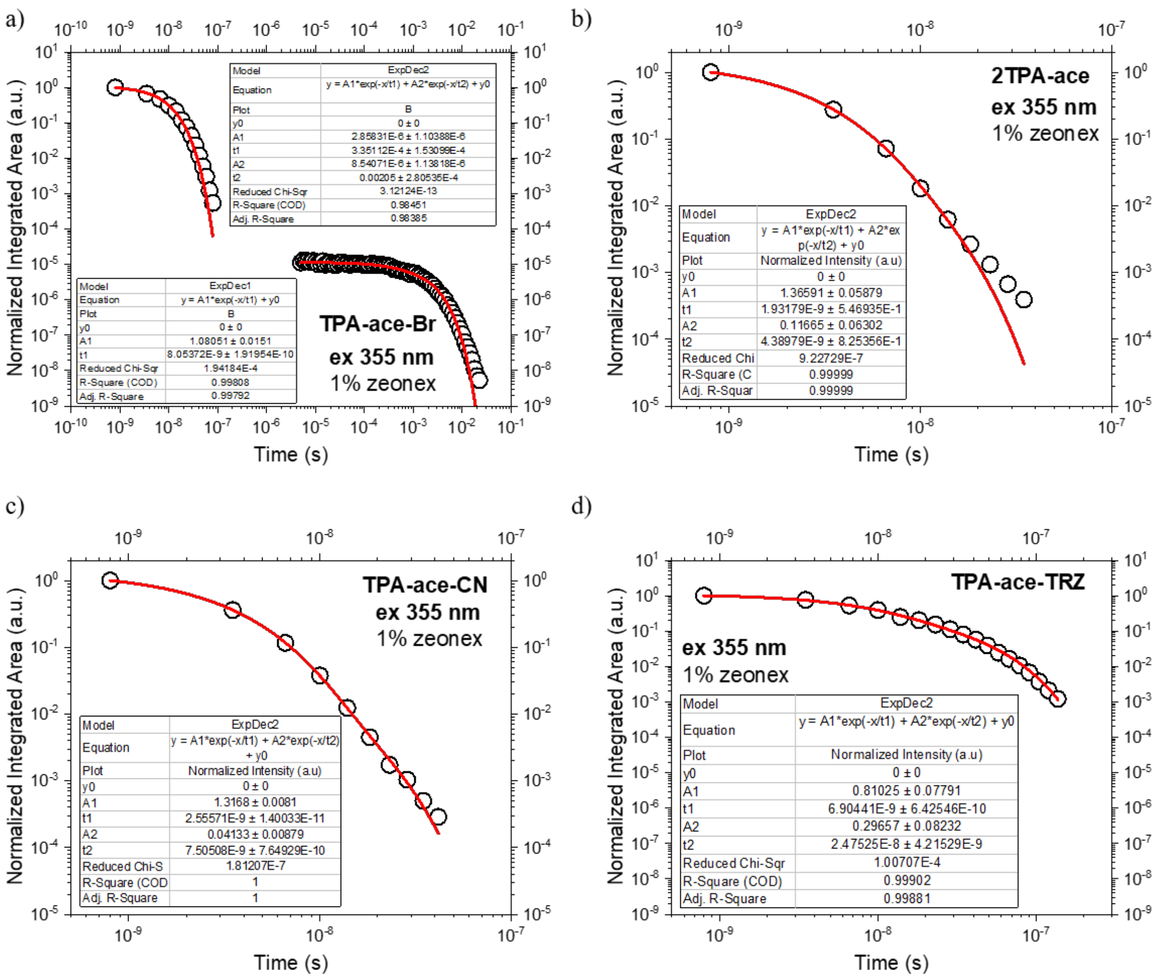

Figure **S46**. Fitting of kinetic decay results for a) **TPA-ace-Br**, b) **2TPA-ace**, c) **TPA-ace-CN** and d) **TPA-ace-TRZ** in 1% ZEONEX film.

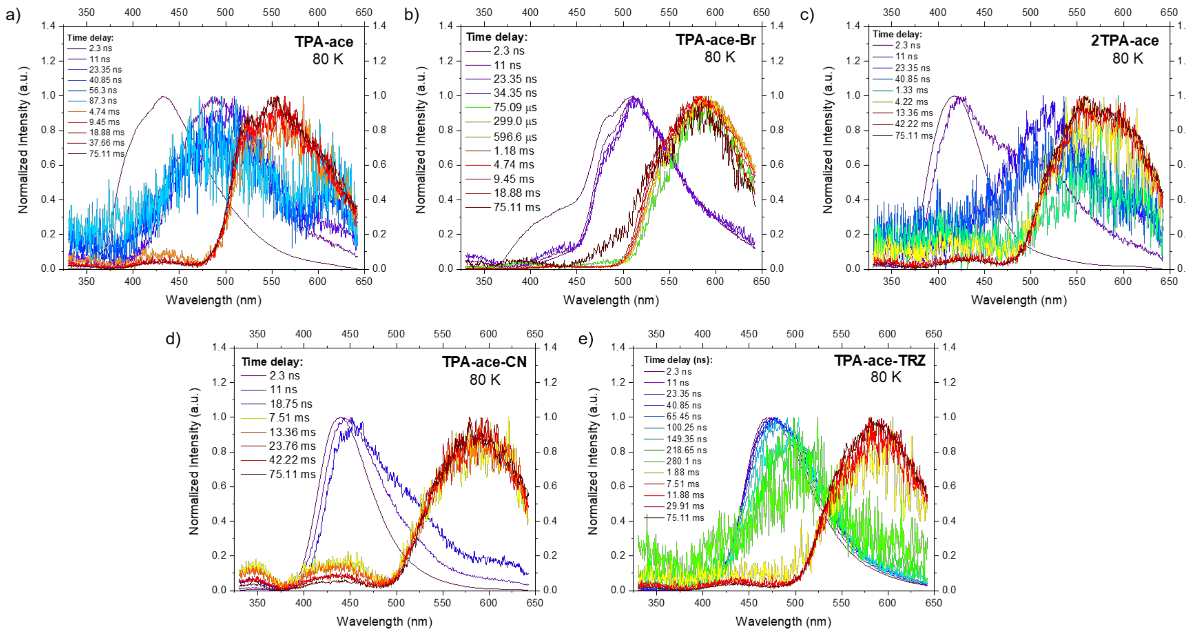

Figure **S47**. Time resolved normalized emission spectra of a) **TPA-ace**, b) **TPA-ace-Br,** c) **2TPA-ace**, d) **TPA-ace-CN** and b) **TPA-ace-TRZ** in 1% ZEONEX films. d) Time resolved fluorescence decay curves in the entire region of analyses. The curves were obtained with 355 nm excitation.

References

[1] Frisch, M. J.; Trucks, G. W.; Schlegel, H. B.; Scuseria, G. E.; Robb, M. A.; Cheeseman, J. R.; Scalmani, G.; Barone, V.; Petersson, G. A.; Nakatsuji, H.; Li, X.; Caricato, M.; Marenich, A. V.; Bloino, J.; Janesko, B. G.; Gomperts, R.; Mennucci, B.; Hratchian, H. P.; Ortiz, J. V.; Izmaylov, A. F.; Sonnenberg, J. L.; Williams; Ding, F.; Lipparini, F.; Egidi, F.; Goings, J.; Peng, B.; Petrone, A.; Henderson, T.; Ranasinghe, D.; Zakrzewski, V. G.; Gao, J.; Rega, N.; Zheng, G.; Liang, W.; Hada, M.; Ehara, M.; Toyota, K.; Fukuda, R.; Hasegawa, J.; Ishida, M.; Nakajima, T.; Honda, Y.; Kitao, O.; Nakai, H.; Vreven, T.; Throssell, K.; Montgomery Jr., J. A.; Peralta, J. E.; Ogliaro, F.; Bearpark, M. J.; Heyd, J. J.; Brothers, E. N.; Kudin, K. N.; Staroverov, V. N.; Keith, T. A.;

Kobayashi, R.; Normand, J.; Raghavachari, K.; Rendell, A. P.; Burant, J. C.; Iyengar, S. S.; Tomasi, J.; Cossi, M.; Millam, J. M.; Klene, M.; Adamo, C.; Cammi, R.; Ochterski, J. W.; Martin, R. L.; Morokuma, K.; Farkas, O.; Foresman, J. B.; Fox, D. J. *Gaussian 09 Rev. D.01*, Wallingford, CT, 2009.

[2] Adamo, C.; Barone, V., *J. Chem. Phys.* **1999,** *110*, 6158-6170.

[3] Hehre, W. J.; Ditchfield, R.; Pople, J. A., *J. Chem. Phys.* **1972,** *56*, 2257-2261.

[4] Hirata, S.; Head-Gordon, M., *Chem. Phys. Lett.* **1999,** *314*, 291-299.

[5] Cardona, C. M.; Li, W.; Kaifer, A. E.; Stockdale, D.; Bazan, G. C., *Adv. Mater.* **2011,** *23*, 2367-2371.

[6] Panchenko, P. A.; Arkhipova, A. N.; Zakharko, M. A.; Jonusauskas, G.; Fedorov, Y. V.; Fedorova, O. A., *Russ. Chem. Bull.* **2016,** *65*, 2444-2451.

[7] Nietfeld, J. P.; Schwiderski, R. L.; Gonnella, T. P.; Rasmussen, S. C., *J. Org. Chem.* **2011,** *76*, 6383-6388.

[8] *CrystalClear-SM Expert* v2.1; Rigaku Americas, The Woodlands, Texas, USA, and Rigaku Corporation, Tokyo, Japan, **2015**.

[9] *CrysAlisPro* v1.171.38.46; Rigaku Oxford Diffraction, Rigaku Corporation, Oxford, U.K. , **2015**.

[10] Burla, M. C.; Caliandro, R.; Camalli, M.; Carrozzini, B.; Cascarano, G. L.; De Caro, L.; Giacovazzo, C.; Polidori, G.; Spagna, R., *J. Appl. Crystallogr.* **2005,** *38*, 381-388.

[11] Sheldrick, G., *Acta Crystallogr. Sect. C* **2015,** *71*, 3-8.

[12] *CrystalStructure* v4.3.0; Rigaku Americas, The Woodlands, Texas, USA, and Rigaku Corporation, Tokyo, Japan, **2018**.
